# Reversible assembly of enantiomeric helical polymers: from fibers to gels†
†Electronic supplementary information (ESI) available. See DOI: 10.1039/c4sc02401j


**DOI:** 10.1039/c4sc02401j

**Published:** 2014-09-02

**Authors:** Seila Leiras, Félix Freire, Emilio Quiñoá, Ricardo Riguera

**Affiliations:** a Department of Organic Chemistry and Center for Research in Biological Chemistry and Molecular Materials (CIQUS) , University of Santiago de Compostela , E-15782 Santiago de Compostela , Spain . Email: felix.freire@usc.es ; Email: ricardo.riguera@usc.es

## Abstract

A novel class of stereocomplexes is described by the interaction of helically complementary poly(phenylacetylene)s (PPAs) carrying an α-methoxy-α-trifluoromethylphenylacetamide pendant group. The formation of the stereocomplex requires the presence of *cis* amide bonds on the external crest of the polymer to provide efficient cooperative supramolecular hydrogen bonding between matching enantiomeric helical structures. The interlocking of the chains gives rise to supramolecular fiber-like aggregates that, at higher concentrations, result in gels. The modification of the *cis*–*trans* amide conformation at the pendant groups allows the controlled formation and cleavage of the stereocomplex due to a dramatic change between the intermolecular and intramolecular hydrogen bond interactions.

## Abstract 2

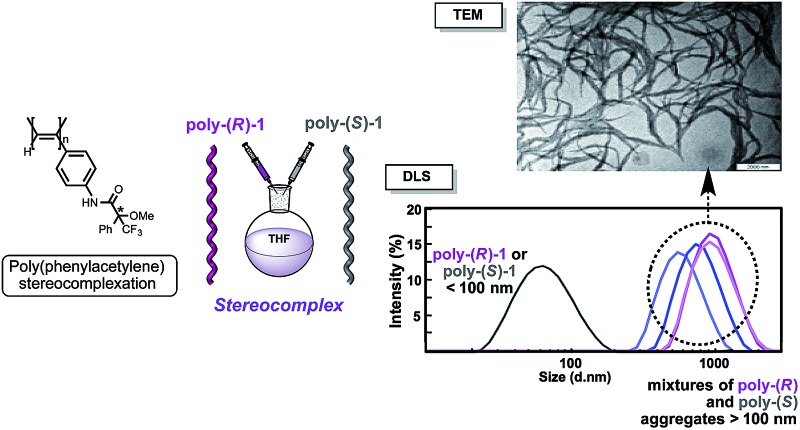
A novel class of stereocomplexes is described by the interaction of helically complementary poly(phenylacetylene)s (PPAs) carrying an α-methoxy-α-trifluoromethylphenylacetamide pendant group.

## Introduction

Stereocomplexes are supramolecular entities formed by the interaction of complementary stereoregular polymers, generating a new material with different properties in comparison with the parent polymers.^1,2^

Well known examples of homo-stereocomplexes are those formed by the association of an isotactic with a syndiotactic polymer (for instance, poly(methyl methacrylate)s (*it*- and *st*-PMMAs)),^3^ those formed by the interaction between two enantiomeric polymers (*e.g.* poly-d- and poly-l-(lactic acid) (PLA)),^4^ or those made from various polypeptides (d- and l-amino acids)^5^ and polyamides (*e.g.* d- and l-poly(hexamethylene di-*O*-methyltartaramide)s).^6^

The formation of stereocomplexes requires two complementary scaffolds, which are linked together through supramolecular interactions. Those made from PMMA and PLA have been amply studied during the last few decades, showing that the existence of stereoselective van der Waals forces is crucial for the stabilization of PMMA stereocomplexes, while those made from PLA seem to be stabilized by weak hydrogen bond interactions.^7^

In this paper we describe a new class of homo-stereocomplexes (fiber-like aggregates and gels), made by the interaction of helically complementary poly(phenylacetylene)s (PPAs)^8^ stabilized by cooperative supramolecular hydrogen bonding among *cis* amide groups located on the polymer crest. We will show also that the formation of the stereocomplex is highly specific for the *cis* conformer and its cleavage can be conveniently tuned by interactions with solvents that modify the *cis*–*trans* amide equilibria at the pendant groups—and as a result, the inter- and intramolecular hydrogen bond interactions. This is, to our knowledge, the first example of a stereocomplex that can be easily switched on/off by the use of solvents.

## Results and discussion

**Fig. 1:**
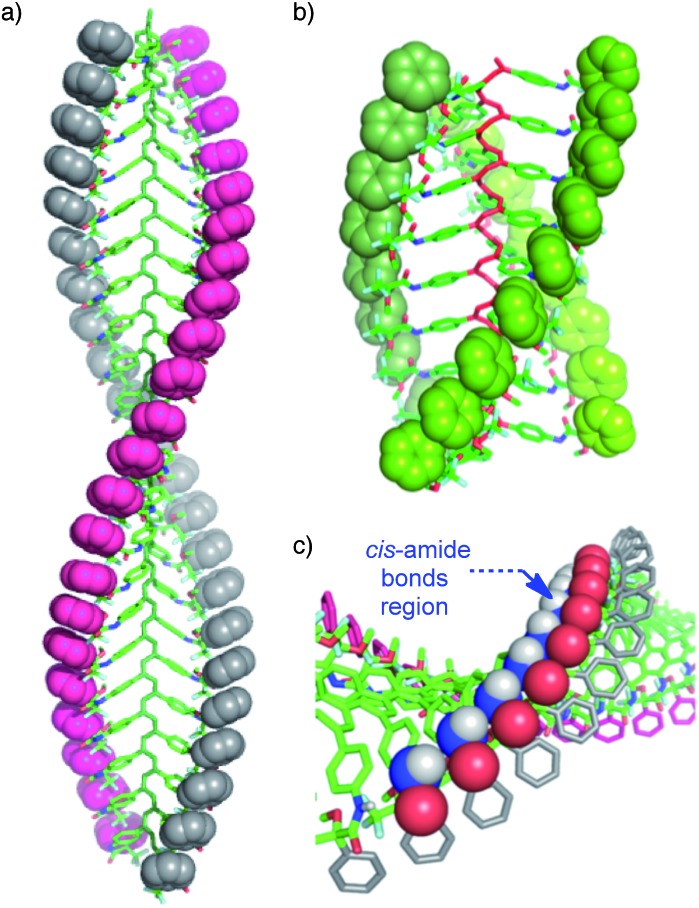
Different helical structures adopted by poly-(*R*)-**1**: (a) 2/1 helix (in donor solvents; *cis* pendant groups) and (b) 3/1 helix (in non-donor solvents; *trans* pendant groups). (c) Fragment of the 2/1 helical structure showing the *cis* amide bonds on the crest of the helix.

**Fig. 2:**
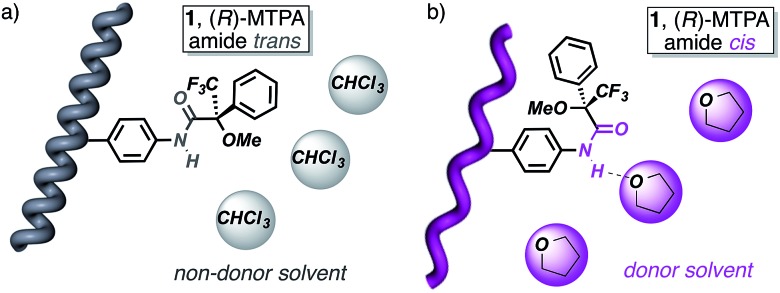
Different helical structures adopted by poly-(*R*)-**1** and the (*R*)-MTPA pendant groups: (a) 3/1 helix (in non-donor solvents; *trans* pendant groups) and (b) 2/1 helix (in donor solvents; *cis* pendant groups).

The starting polymers [poly-(*R*)-**1** and poly-(*S*)-**1**] are prepared by the polymerization of (*R*)- and (*S*)-4-*N*-α-methoxy-α-trifluoromethylphenylacetamides (MTPAs) of 4-ethynylaniline [M-(*R*)-**1** and M-(*S*)-**1**, respectively]. Poly-(*R*)-**1** and poly-(*S*)-**1** present 3/1 helical structures in non-donor solvents (*e.g.* CH_2_Cl_2_, CHCl_3_) with preponderant right-handed [poly-(*R*)-**1**] and left-handed [poly-(*S*)-**1**] helical senses for the backbone frameworks stabilized by intramolecular hydrogen bonds among the *trans* amides of the pendant groups (Fig. 1 and 2).^9^

When a donor solvent (*e.g.* THF) is used to dissolve these polymers, the stereochemistry of the amide bonds changes to *cis*, the intramolecular associations disappear by competition with the donor solvent, and as a result the backbone shifts to a more extended 2/1 helix with opposite helical sense, now determined by steric hindrance among the pendant groups (Fig. 1 and 2).^9^

The existence of intermolecular interactions between poly-(*R*)-**1** and poly-(*S*)-**1** was evaluated by circular dichroism (CD), dynamic light scattering (DLS) and scanning electron microscopy (SEM) by using mixtures with different ratios of the starting polymers in THF and CHCl_3_.

**Fig. 3:**
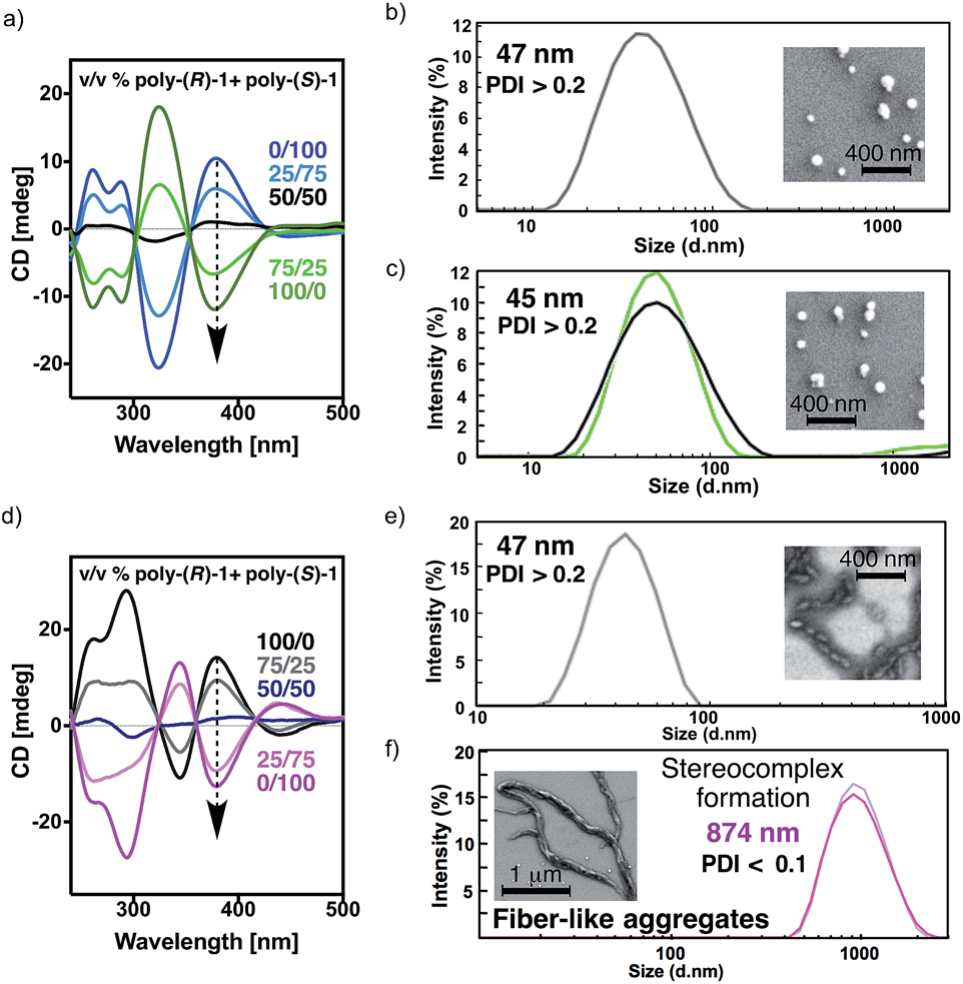
Studies of the stereocomplex formation by CD and DLS: (a) CD spectra of different poly-(*R*)-**1**/poly-(*S*)-**1** mixtures in CHCl_3_ (0.1 mg polymer per mL). DLS traces and SEM images of a solution of (b) 100% poly-(*R*)-**1** in CHCl_3_ (0.1 mg mL^–1^) and (c) mixtures of poly-(*R*)-**1**/poly-(*S*)-**1** at 50/50 (v/v) ratio in CHCl_3_. (d) CD spectra of different poly-(*R*)-**1**/poly-(*S*)-**1** mixtures in THF (0.1 mg polymer per mL). DLS traces and SEM images of a solution of (e) 100% poly-(*R*)-**1** in THF (0.1 mg mL^–1^) and (f) mixtures of poly-(*R*)-**1**/poly-(*S*)-**1** at a ratio of 50/50 (v/v) in THF.

The CD spectra for all mixtures and in both solvents show signatures corresponding to the contribution of the individual components in the given ratio, indicating that the helical structures of poly-(*R*)-**1** and poly-(*S*)-**1** remain unaltered (Fig. 3a and d).

The DLS traces and SEM images of poly-(*R*)-**1** in both CHCl_3_ (Fig. 3b) and THF (Fig. 3e) indicated the formation of polydisperse particles with diameters around 45 nm. The same types of particles were obtained from the mixture of poly-(*R*)-**1**/poly-(*S*)-**1** at a 50/50 (v/v) ratio (Fig. 3c) in CHCl_3_. In contrast, when this 50/50 (v/v) poly-(*R*)-**1**/poly-(*S*)-**1** mixture was formed in THF, large fiber-like aggregates were observed (Fig. 3f).

Similar experiments were carried out with several poly-(*R*)-**1**/poly-(*S*)-**1** mixtures at different ratios. It was found that the presence of just 2% (v/v) of one component [*e.g.* 20 μL of poly-(*S*)-**1** (0.1 mg mL^–1^)/980 μL of poly-(*R*)-**1** (0.1 mg mL^–1^)] was enough to induce the formation of supramolecular entities larger than 400 nm (see ESI†). Moreover, the size of the aggregates can be controlled by the concentration of the starting solution at any poly-(*R*)-**1**/poly-(*S*)-**1** ratio, becoming larger as the concentration increases (*e.g.* 50/50 (v/v) mixtures at 0.05 mg mL^–1^ afford particles of 635 nm diameter, while those at 0.5 mg mL^–1^ give particles of 1338 nm diameter; see ESI†).

In summary, these results indicate not only the high specificity but also the effectiveness of the aggregation process that can be triggered by the presence of just 2% of the complementary polymer.

Additional information about the aggregates, the mechanism of aggregation and the requirements of the starting polymers are described below.

### Reversibility of the stereocomplex formation

The structures of the starting polymers poly-(*R*)-**1** and poly-(*S*)-**1** in THF suggest that the helically complementary structures combine by intermolecular association involving hydrogen bonds among the amide groups. If this is so, temperature changes or the addition of solvents (*e.g.* MeOH) able to disturb hydrogen bond formation, without interfering with the secondary structures of the polymers (*i.e.* neither *cis*–*trans* isomerization nor helical inversion, see pages S28–S31, ESI†), should produce the cleavage of the stereocomplex through intermolecular hydrogen bonding disruption.

**Fig. 4:**
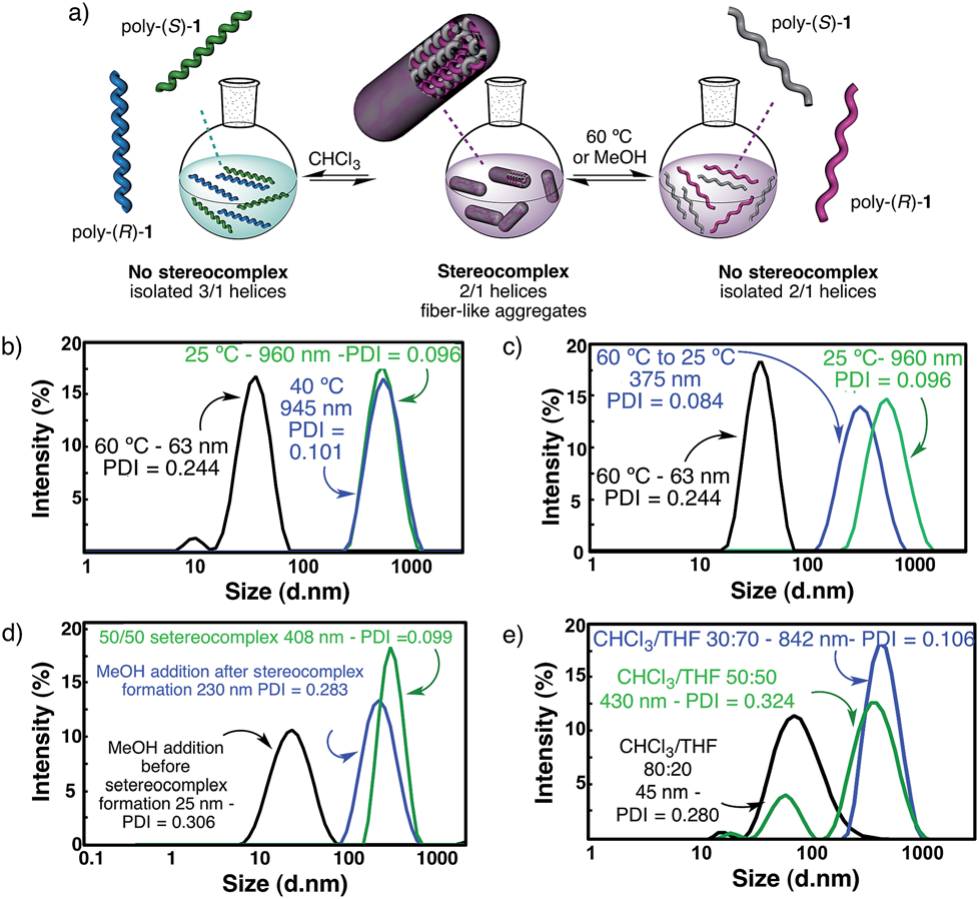
Stereocomplex disruption studies of a poly-(*R*)-**1**/poly-(*S*)-**1** THF solution in a 50/50 (v/v) ratio by DLS. (a) Graphical scheme showing the stereocomplex disruption by different external stimuli. (b) DLS traces of solutions of the stereocomplex in THF at different temperatures: the stereocomplex is stable at 25 °C and 40 °C (in green and blue) and is disrupted at 60 °C (in black). (c) DLS traces corresponding to the 50/50 (v/v) poly-(*R*)-**1**/poly-(*S*)-**1** stereocomplex in THF at 25 °C and heated to 60 °C (in black), and the recovery of the aggregation after cooling down from 60 °C to 25 °C and staying at that temperature for 1 h (in blue, the stereocomplex is formed again). The original trace of the stereocomplex at 25 °C (prior to the heating) is shown in green. (d) DLS traces showing: the effect of the addition of MeOH to the initial solution of poly-(*R*)-**1** in THF, before the addition of poly-(*S*)-**1** (in black, formation of the stereocomplex does not take place) and the effect of the addition of MeOH (in blue, fiber-like aggregates remain in solution) to the preformed 50/50 poly-(*R*)-**1**/poly-(*S*)-**1** stereocomplex (in green). (e) DLS measurements showing the disruption of the stereocomplex at different CHCl_3_/THF ratios (30/70, 50/50 and 80/20).

As a matter of fact, the DLS analysis of the 50/50 (v/v) mixture in THF at 60 °C showed only the presence of isolated polymers, indicating that no stereocomplex is present at that temperature (Fig. 4b). Furthermore, when the temperature is decreased to room temperature, the stereocomplex is recovered, showing the reversibility of its formation in solution (Fig. 4c). The size of the recovered stereocomplex after heating at 60 °C is not completely identical to that of the pristine stereocomplex, *i.e.* a change in size from 960 nm (pristine stereocomplex) to 375 nm (recovered stereocomplex) is observed.^10^ The reassembly among polymer chains after the heating/cooling cycle may not be strictly identical to the previous association since there are assembly points all over the polymer chain that allow a more compact aggregation.

Furthermore, if a hydrogen donor solvent such as methanol is added to the initial solution of one of the starting polymers in THF prior to the addition of the other component, the formation of the stereocomplex does not take place because the MeOH is capping the *cis* amide hydrogens through hydrogen bond interactions. On the contrary, if the methanol is added to the preformed stereocomplex, the fiber-like aggregates are only partially cleaved and remain in solution as smaller sized suprastructures (Fig. 4d). Finally, if this solution is heated up to 60 °C, the sterecomplexation is disrupted, and when the THF/MeOH solution is cooled down, the stereocomplex formation does not take place (see a more detailed description of the phenomenon on pages S29 and S30, ESI,† including Fig. S31 and S32†).

The cleavage of the stereocomplex can be performed not only by heat or hydrogen bond competition with MeOH, but also by the addition of solvents that decrease the donor ability of the media. Thus, the addition of increasing amounts of CHCl_3_ to the solution of the stereocomplex in THF results in its effective disruption due to the promotion of the *cis* amide bonds towards the *trans* conformation—the latter being involved in intramolecular hydrogen bond interactions—and thus, from a *cis*–*transoid* 2/1 to a *cis*–*cisoid* 3/1 helix. Therefore, the intermolecular hydrogen bonds are disrupted (Fig. 4e). See a more detailed description of the phenomenon on page S31, ESI,† including Fig. S33.†

All these data indicate that the aggregate is stabilized by hydrogen bonds among the *cis* amide groups located at the external parts of the polymer chains. Interestingly, the IR spectrum of the poly-(*R*)-**1**/poly-(*S*)-**1** mixture in THF shows the characteristic band for *cis* amides (see ESI†), identical to the one found for the individual polymers in THF and different from the *trans* band found for the polymers in CHCl_3_.^9^ Therefore, we conclude that the formation of the stereocomplex depends on two factors: *cis* amide functions at the crests of the polymer chains and complementary helices.

### Thermal studies on the solid phase

Differential scanning calorimetry (DSC) experiments were carried out in order to obtain further information on the thermal properties of the stereocomplex and its secondary structure. This technique has been shown to be useful for obtaining information on the thermal isomerizations taking place in these kinds of macromolecules [*e.g. cis*–*transoidal* to *cis*–*cisoidal* (*c*–*t* to *c*–*c*); *cis*–*cisoidal* to *trans*–*transoidal* (*c*–*c* to *t*–*t*)].^11^

Hence, films were prepared from the 50/50 (v/v) poly-(*R*)-**1**/poly-(*S*)-**1** mixture in THF and in CHCl_3_ (see ESI† for the experimental procedure), and the DSC traces obtained were compared with those from the parent polymers in the same solvents.

**Fig. 5:**
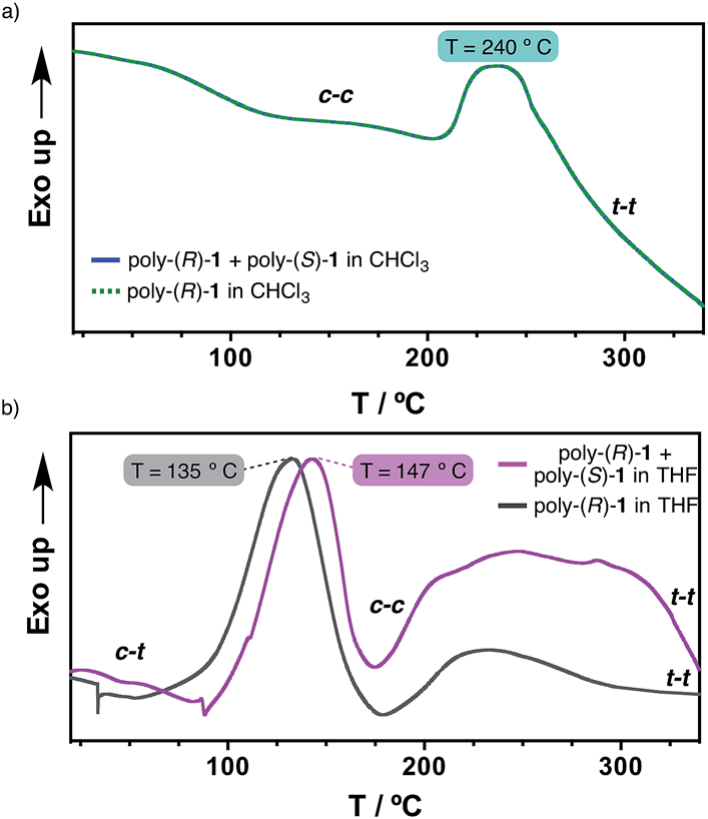
Studies of the 50/50 poly-(*R*)-**1**/poly-(*S*)-**1** stereocomplex disruption by DSC. (a) DSC thermograms of poly-(*R*)-**1** and 50/50 poly-(*R*)-**1**/poly-(*S*)-**1** (CHCl_3_ films). (b) DSC thermograms of poly-(*R*)-**1** and the 50/50 poly-(*R*)-**1**/poly-(*S*)-**1** stereocomplex (THF films).

The results revealed identical DSC signatures for the films from poly-(*R*)-**1** and from the poly-(*R*)-**1**/poly-(*S*)-**1** mixture when they were prepared in CHCl_3_ (Fig. 5a). This fact confirms that no new species are being formed when mixing poly-(*R*)-**1**/poly-(*S*)-**1** in that solvent. However, when the same films are prepared in THF, the poly-(*R*)-**1**/poly-(*S*)-**1** mixture shows the *c*–*t* to *c*–*c* transition at 147 °C while the parent poly-(*R*)-**1** exhibits the maximum at 135 °C. This delay clearly indicates the presence of a new entity, the stereocomplex, different from the parent polymer, with higher thermal stability than the starting components (Fig. 5b).

Additionally, the first cooling and second heating processes for the poly-(*R*)-**1**/poly-(*S*)-**1** film prepared in THF showed no transition peaks, indicating that both the isomerization of the backbone and the disruption of the stereocomplex are not reversible under thermal treatment in the solid state (see ESI†).

### Electron microscopy

**Fig. 6:**
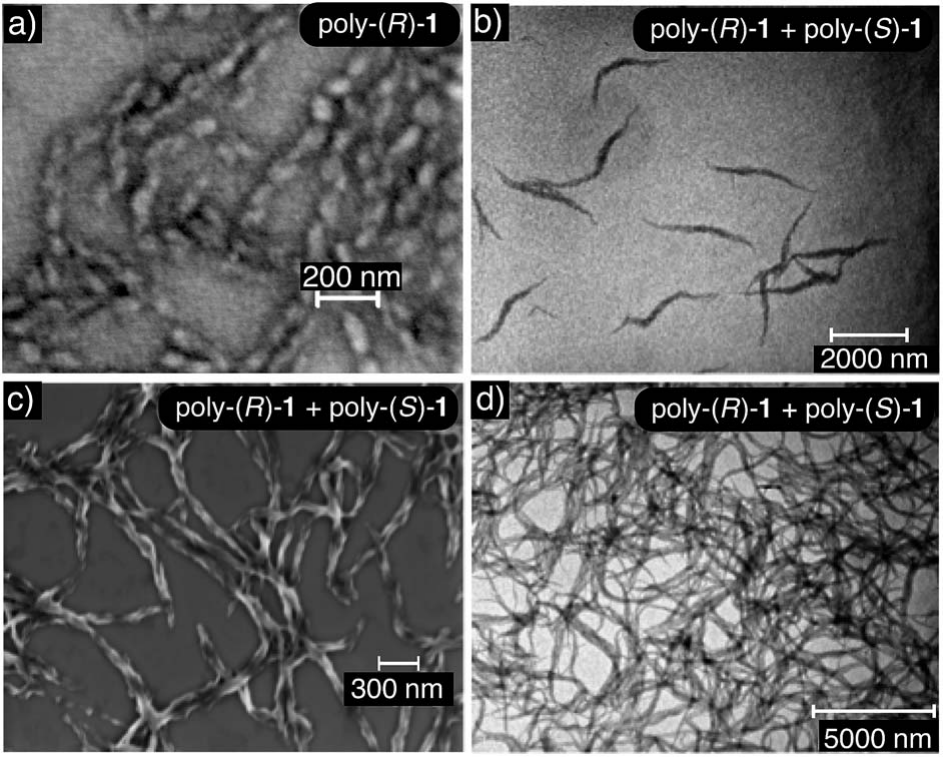
(a) SEM image of poly-(*R*)-**1** (concentration = 0.1 mg mL^–1^ THF) showing nanoparticles (scale bar = 200 nm). (b) TEM and (c) SEM images showing the nanofibers obtained for 50/50 poly-(*R*)-**1**/poly-(*S*)-**1** (concentration = 0.1 mg mL^–1^ THF) (TEM scale bar = 2000 nm, SEM scale bar = 300 nm). (d) TEM image showing a gel obtained for 50/50 poly-(*R*)-**1**/poly-(*S*)-**1** (concentration = 0.5 mg mL^–1^ THF) (scale bar = 5000 nm).

Transmission electron microscopy (TEM) and scanning electron microscopy (SEM) of poly-(*R*)-**1** and the poly-(*R*)-**1**/poly-(*S*)-**1** stereocomplex confirmed the different natures of the aggregates from both systems: the SEM image of poly-(*R*)-**1** (0.1 mg mL^–1^) in THF showed polydisperse particles around 45 nm in size, in accordance with the DLS results (Fig. 6a), whereas the TEM and SEM images of the poly-(*R*)-**1**/poly-(*S*)-**1** mixture (0.1 mg mL^–1^) showed fiber-like aggregates (Fig. 6b and c). At higher concentrations (0.5 mg mL^–1^), the poly-(*R*)-**1**/poly-(*S*)-**1** stereocomplex evolved from fiber-like aggregates to a gel structure (Fig. 6d).

### Rheology studies

In order to characterize the gel-like supramolecular structure (Fig. 6d), comparative rheological studies were carried out and the results are shown below.

**Fig. 7:**
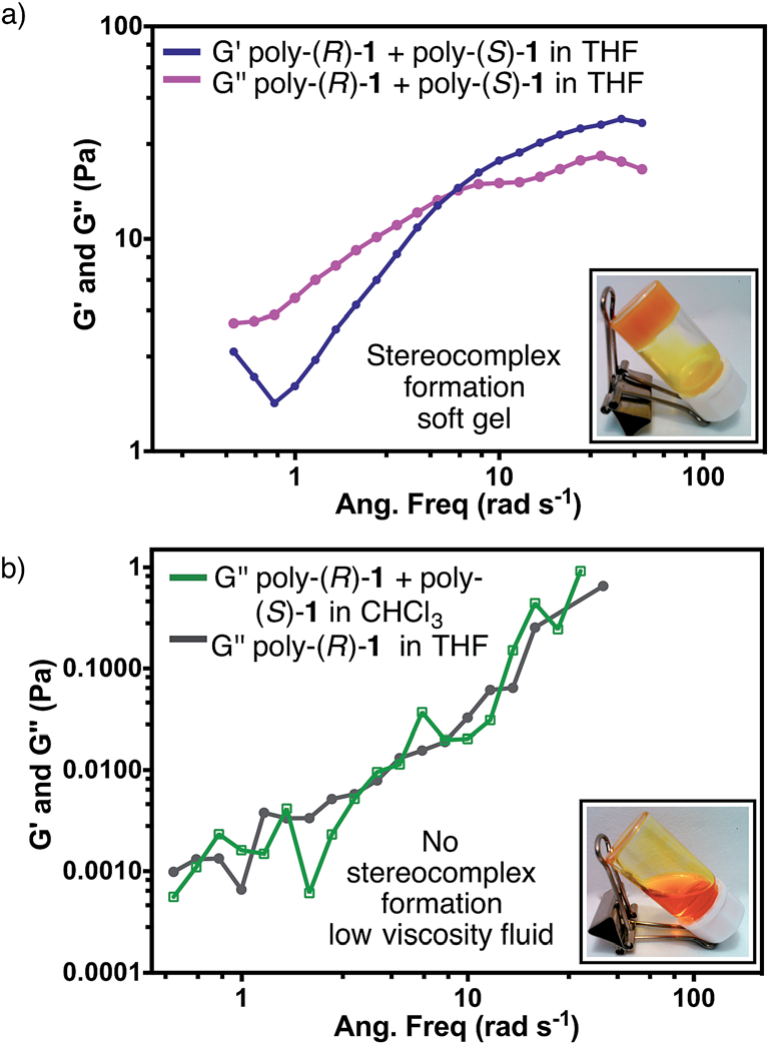
(a) Rheological studies for the stereocomplex in THF (concentration = 5 mg mL^–1^). (b) Comparison between the rheological parameter *G*′′ of a mixture of poly-(*R*)-**1** and poly-(*S*)-**1** in CHCl_3_ and of a solution of poly-(*R*)-**1** in THF (concentration = 5 mg mL^–1^).

Solutions of the individual polymers [poly-(*R*)-**1** or poly-(*S*)-**1**] in either THF or CHCl_3_ exhibit viscous behaviour even at 5 mg mL^–1^, with low *G*′′ and negligible *G*′ values (see ESI†). On the other hand, the rheological parameters of solutions of the 50/50 (v/v) poly-(*R*)-**1**/poly-(*S*)-**1** mixture in THF revealed a strong dependence on the total concentration. At 0.5 mg mL^–1^, only low *G*′′ values were detected in the stereocomplex solution (see ESI†). At 2.5 mg mL^–1^ the solution presented the typical behaviour of a Maxwell fluid: *G*′′ was larger than *G*′ at low frequencies and increased in a linear fashion with the frequency, with a slope close to 2 (see ESI†). Raising the concentration up to 5 mg mL^–1^, the *G*′′ values became independent of the frequency and the curves of the two moduli showed an intersection at 6.28 rad s^–1^; beyond that frequency, *G*′ values were greater than *G*′′ (Fig. 7a). These results confirm that at high concentrations the stereocomplex behaves as a soft gel. Similar to what happens in dilute solutions, the on/off switching of the stereocomplex formation can be attained in the gel state by changing the temperature and solvent (see Fig. S60 and S61, ESI†).^12^

Finally, as could be expected from the previous CD, SEM and TEM results, similar solutions of the mixture [poly-(*R*)-**1**/poly-(*S*)-**1** at a 50/50 (v/v) ratio] in CHCl_3_ instead of THF behave as a low viscosity fluid in the whole range of concentrations tested (Fig. 7b).

### Stereocomplex formation mechanism: modeling

According to our hypothesis, the formation of the fibers and gels originates with the aggregation among complementary helices through hydrogen bonds between the *cis* amide groups at the external crests of the polymer chains. In this sense, it was interesting to analyse the geometrical possibilities of that process among polymer chains with the same or opposite helicity, and with *trans* and *cis* amide conformation at the pendant groups.

**Fig. 8:**
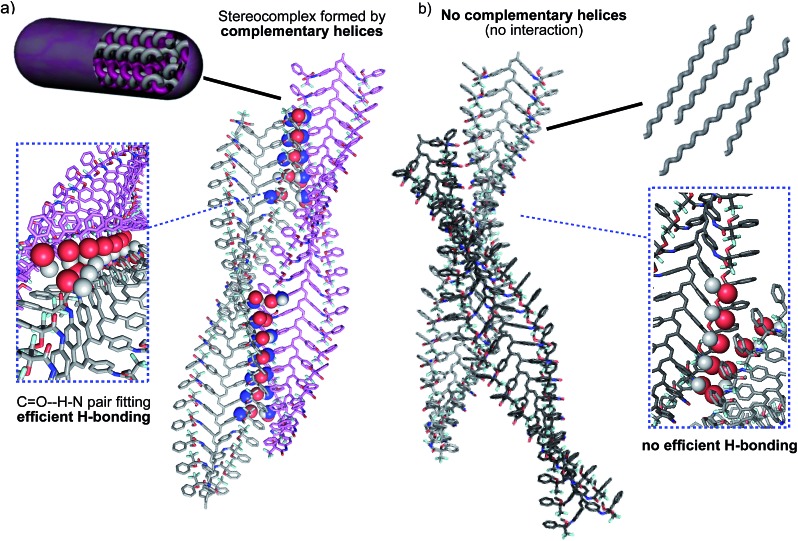
(a) Computer modeling of the complementary helical structures of poly-(*R*)-**1** and poly-(*S*)-**1** forming the stereocomplex. (b) Computer modelling of the non-complementary helices poly-(*R*)-**1** and poly-(*R*)-**1** (hydrogen bonding interactions are not favoured).

The geometrical matching of the complementary helices of poly-(*R*)-**1** and poly-(*S*)-**1**, with *cis* amide bonds on the outside (*e.g.* in THF), was studied by computer modeling. The results indicated that interaction between the *cis* amides of those helices is geometrically feasible (Fig. 8a), and that the formation of a supramolecular aggregate bound together *via* multiple hydrogen bonds would be reasonable. On the contrary, if the amides were placed in a *trans* conformation (*e.g.* in CHCl_3_) no matching between the helices is possible, explaining the absence of aggregate. In fact, we tested experimentally other helical poly(phenylacetylene)s with similar geometrical requirements but containing *trans* amides in the external crests, and found no stereocomplex formation.^8d,e^

Moreover, the modeling also showed that no efficient matching could be produced among helices with the same helical sense, even if they presented well-placed *cis* amide bonds [*e.g.* right-handed helices of poly-(*R*)-**1** in THF, Fig. 8b].

### Monitoring stereocomplex formation by AFM

In order to monitor the formation of the stereocomplexes in the solid state, we decided to carefully examine the AFM images obtained during the formation of the aggregate with mixtures of poly-(*R*)-**1**/poly-(*S*)-**1** in THF, and to compare them with those of the individual polymers.

Thus, AFM studies of poly-(*R*)-**1** in THF (0.01 mg mL^–1^) present images of 2D crystals with single right-handed helix packing, and no evidence of large aggregates.^9^ However, AFM images of a 50/50 (v/v) poly-(*R*)-**1**/poly-(*S*)-**1** mixture in THF showed, in addition to some isolated 2D crystals corresponding to the left-handed [poly-(*S*)-**1**] and right-handed [poly-(*R*)-**1**] helices of the individual polymers, the presence of abundant fiber-like aggregates. More precisely, fibers with diameters around 3.6 and 5.0 nm are clearly distinguished, in addition to higher order fiber aggregates with diameters around 80–100 nm.

**Fig. 9:**
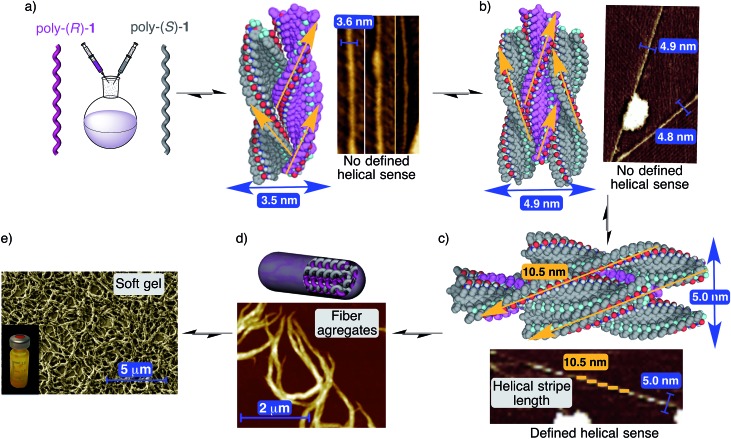
Structure of the stereocomplex. (a) Side view of the stereocomplex dimer model (3.5 nm width) and the corresponding AFM image showing fibers (≈3.7 nm width). (b) Side view of the stereocomplex trimer model (4.9 nm width) and the corresponding AFM image showing fibers (≈4.8 nm width). (c) Side view of the stereocomplex pentamer model (5.0 nm diameter) and the corresponding AFM image showing fibers (≈5.0 nm diameter). (d) Conceptual representation of the stereocomplex fiber formation between complementary helices and the AFM image showing stereocomplex fiber aggregates. (e) Images showing the soft gel structure at microscopic (SEM image) and macroscopic levels (stereocomplex solution in THF, concentration = 25 mg mL^–1^). For more detailed depictions, including frontal views of the different types of fibers, see Fig. S39–S41, ESI.†

The smaller fibers seem to correspond to the initial aggregation steps, and in fact, a width of 3.6 nm fits exactly with the expected one for a dimer (one left-handed helix interacting with one right-handed helix, Fig. 9a). Due to its composition (50/50 mixture of both helices), this dimeric fiber should not present specific helical sense on its surface, in full agreement with the experimentally observed AFM image (Fig. 9a).

As for the fibers with a width of about 4.9–5.0 nm, two different varieties are observed depending on their surface. The first types of these fibers show no specific helical sense on their surface. This characteristic as well as their width (about 4.9–5 nm) fits well with the expected data for a trimer formed by two helices of one sense surrounding one helix with the opposite helical sense (Fig. 9b).

The second types of fibers were found in the AFM images and present quite similar widths to the trimer but with a specific helical sense on their surface (Fig. 9c). These data match well with a pentameric structure in which one helix chain is surrounded by four other helices of the opposite helical sense. In this case, the fiber surface should reflect the combination of the crests of the four external helices (Fig. 9c) and therefore a specific helical sense. Moreover, modeling of this pentameric fiber shows very good fitting among the individual chains, leading to a maximum of about 10.5 nm crest length, in full agreement with the AFM image presented in Fig. 9c, where a single helix of poly-(*R*)-**1** is surrounded by four helices of poly-(*S*)-**1**, producing as a result an external left-handed helical aggregate.

Naturally, due to the presence of *cis* amide bonds on the outer crests, these fiber aggregates with small diameters (dimer, trimer, pentamer…) can keep growing and producing the larger fiber aggregates (with diameters of about 80–100 nm) that at higher concentrations will afford a gel-like structure (Fig. 9d and e).

### The effectivity of the binding mechanism: copolymers

The modeling studies indicated that a good geometrical fitting between complementary polymer chains requires the interaction of only a few *cis* amide groups (no more than 6 in a row per helix crest; Fig. 8a). Besides that, experimentally we know that the addition of just 2% (v/v) of one helix to 98% of the complementary one is enough to initiate the aggregation.

To study the structural requirements for aggregation more deeply, and more precisely, to determine the minimum *cis* amide content in a polymer that is needed for effective aggregation, we decided to prepare copolymers from poly-(*R*)-**1** (or poly-(*S*)-**1**) where some of the units containing *cis* amide at the crests are replaced by similar units containing *trans* amides, and to test their aggregation ability.

**Fig. 10:**
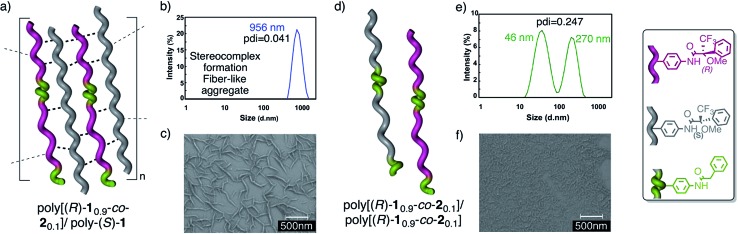
The stereocomplex from the copolymer/polymer mixtures. (a) Conceptual representation of the 50/50 poly[(*R*)-**1**_0.9_-*co*-**2**_0.1_]/poly-(*S*)-**1** stereocomplex. (b) DLS trace of the 50/50 poly[(*R*)-**1**_0.9_-*co*-**2**_0.1_]/poly-(*S*)-**1** stereocomplex. (c) SEM image of the 50/50 poly[(*R*)-**1**_0.9_-*co*-**2**_0.1_]/poly-(*S*)-**1** stereocomplex. (d) Conceptual representation of the low aggregation shown by the 50/50 poly[(*R*)-**1**_0.9_-*co*-**2**_0.1_]/poly[(*S*)-**1**_0.9_-*co*-**2**_0.1_] mixtures. (e) DLS trace of the 50/50 poly[(*R*)-**1**_0.9_-*co*-**2**_0.1_]/poly[(*S*)-**1**_0.9_-*co*-**2**_0.1_] mixture. (f) SEM image of the 50/50 poly[(*R*)-**1**_0.9_-*co*-**2**_0.1_]/poly[(*S*)-**1**_0.9_-*co*-**2**_0.1_] mixture.

Thus, copolymers poly[(*R*)-**1**_0.9_-*co*-**2**_0.1_] and poly[(*R*)-**1**_0.8_-*co*-**2**_0.2_]^13^ were prepared by the copolymerization of M-(*R*)-**1** and M-**2**, [M-**2** = *N*-(4-ethynylphenyl)-2-phenylacetamide], and mixed with poly-(*S*)-**1**. Next, the aggregation was checked by DLS and SEM experiments (Fig. 10).

The results indicated that while poly[(*R*)-**1**_0.9_-*co*-**2**_0.1_] (10% *trans* amide) is able to form a stereocomplex in the presence of poly-(*S*)-**1** (Fig. 10a–c), when the percentage of the *trans* amide monomer is increased to 20%—poly[(*R*)-**1**_0.8_-*co*-**2**_0.2_]—no stereocomplex is formed by the interaction with poly-(*S*)-**1**.

Similarly, the 50/50 mixture of the copolymers poly[(*R*)-**1**_0.9_-*co*-**2**_0.1_]/poly[(*S*)-**1**_0.9_-*co*-**2**_0.1_] (20% *trans* amide content overall) was found not to form the stereocomplex (Fig. 10d–f) (see the full description in the ESI†).

## Conclusions

In this paper we present the first example of a stereocomplex formed by the association of helical poly(phenylacetylene)s. The formation of this fiber-like aggregate, that becomes a gel at high concentrations, requires the helical complementarity of the starting polymers and the presence, at the external part of their skeleton, of *cis* amide groups that provide hydrogen bonding to interconnect the helices. The binding and cleavage mechanism, and the structural and physical properties have been discussed. This is, to our knowledge, the first example of a stereocomplex whose formation can be switched on and off by modulation of the *cis*–*trans* amide conformation of the pendant groups.
