# Supplementary material for: Reversible assembly of enantiomeric helical polymers: from fibers to gels
Source: Chem Sci. 2014 Sep 2;6(1):246–53. doi: 10.1039/c4sc02401j (PMC5433055; doi:10.1039/c4sc02401j)
Supplement: Supplementary file 1 [file SC-006-C4SC02401J-s001.pdf]

Electronic Supplementary Material (ESI) for Chemical Science.
This journal is © The Royal Society of Chemistry 2014

Reversible Assembly of Enantiomeric Helical Polymers: From Fibers to Gels

Seila Leiras, Félix Freire*, Emilio Quiñoá, Ricardo Riguera*

Center for Research in Biological Chemistry and Molecular Materials (CIQUS) and Department of Organic Chemistry, University of Santiago de Compostela, E-15782 Santiago de Compostela, Spain

felix.freire@usc.es, ricardo.riguera@usc.es

http://www.usc.es/gi1608/

Supporting Information

Contents:

1. **Materials and methods** **S3**

2. **Synthesis** **S5**
   - 2.1. **Synthesis of monomers M-(*R*)- and M-(*S*)-1** **S5**
   - 2.2. **Synthesis of monomer 2** **S8**
   - 2.3. **Synthesis of polymers poly-(*R*)-1 and poly-(*S*)-1** **S10**
   - 2.4. **Synthesis of polymers poly[(*R*)-1-co-2] and poly[(*S*)-1-co-2]** **S15**

3. **Determination of the monomer reactivity ratios in copolymerizations** **S19**

4. **Poly-(*R*)-1 + Poly-(*S*)-1 CD and UV-Vis** **S21**

5. **Poly-[(*R*)-1-co-2] and Poly-[(*S*)-1-co-2] CD and UV-Vis** **S23**

6. **Poly-(*R*)-1 aggregation studies by Dynamic Light Scattering (DLS) in different solvents** **S24**

7. **Poly-(*R*)-1 aggregation studies by Scanning Electron Microscopy (SEM) in different solvents.** **S25**

8. **Poly-(*R*)-1 + Poly-(*S*)-1 aggregation studies by Dynamic Light Scattering (DLS) in different solvents** **S25**

9. **Poly-(*R*)-1 + Poly-(*S*)-1 aggregation studies by Dynamic Light Scattering (DLS) in THF** **S26**

10. **Reversibility of the stereocomplex formation** **S28**
    - 10.1. **Increasing temperature** **S28**
    - 10.2. **Addition of MeOH** **S29**
    - 10.3. **Addition of a non-donor solvent** **S31**

11. **Poly-(*R*)-1 + Poly-(*S*)-1 aggregation studies by Transmission Electron Microscopy (TEM) in THF** **S32**

12. **Poly-(*R*)-1 + Poly-(*S*)-1 aggregation studies by Scanning Electron Microscopy (SEM) in THF** **S34**

13. **Poly-(*R*)-1 + Poly-(*S*)-1 aggregation studies by Scanning Electron Microscopy (SEM) in $CHCl_3$ and DMF.** **S35**

14. **Poly-(*R*)-1 + Poly-(*S*)-1 aggregation studies by Atomic Force Microscopy (AFM) in THF** **S36**

15. **Rheology studies** **S43**

16. **Poly-[(*R*)-1-co-2] and Poly-[(*S*)-1-co-2] aggregation studies by Dynamic Light Scattering (DLS) in THF** **S44**

17. **Poly-[(*R*)-1-co-2] and Poly-[(*S*)-1-co-2] aggregation studies by Scanning Electron Microscopy (SEM) in THF** **S45**

18. **Differencial Scaning Calorimetry (DSC) studies of Poly-(*R*)-1 and [Poly-(*R*)-1 + Poly-(*S*)-1] films** **S46**
    - 18.1. **Poly-(*R*)-1 film prepared in $CHCl_3$ and THF:** **S46**
    - 18.2. **[Poly-(*R*)-1 + Poly-(*S*)-1] film prepared in $CHCl_3$ and THF** **S48**

19. **Gel-state on/off switching** **S50**

1. Materials and methods

CD measurements were done in a Jasco-720. The amount of polymers and copolymers used for CD measurements were 0.1 mg/mL.

UV spectra were registered in a Jasco V-630. The amount of polymers and copolymers used for CD measurements were 0.1 mg/mL.

DLS measurements were performed on a Malvern Nano ZS (Malvern Instruments, U.K.), operating at 633 nm with a 173º scattering angle. These studies indicate that the stereocomplex form well defined aggregates.

FT-IR spectra were recorded in a Bruker IFS-66v spectrophotometer. CsI cells were used. A solution of the polymers (5 mg/mL) was prepared in THF and the solvent was removed by evaporation.

Raman spectra were carried out in a Renishaw confocal raman spectrometer (Invia Reflex model), equipped with two laser (diode laser 785 nm and Ar laser 514 nm).

DSC traces were obtained in a DSC Q200 Tzero Technology (TA Instruments, New Castle, UK), equipped with a refrigerated cooling system RCS90 (TA Instruments, New Castle, UK), using a Tzero low-mass aluminum pan.

Viscoelastic behavior of dispersions containing poly-(*R*)-**1**, poly-(*S*)-**1** or a mixture of both [poly-(*R*)-**1**/poly-(*S*)-**1** ratio of 1/1 (v/v)] were evaluated at 20ºC in a Rheolyst AR-1000N rheometer (TA Instruments, New Castle, UK) equipped with an AR2500 data analyzer, and fitted with a Peltier plate. The storage (G') and the loss (G'') moduli were recorded at 0.1 Pa in the 0.5-50 rad/s angular frequency interval using a cone-plate geometry (diameter 4 cm, angle 2º) with solvent trap.

AFM measurements were performed in a MultiMode V Scanning Probe Microscope (Veeco Instruments) in air at RT with standard silicon cantilevers and super-sharp cantilevers in tapping mode using 12 μm and 1 μm scanners. Nanoscope processing software and WSxM 4.0 Beta 1.0 [4] (Nanotec Electronica, S.L.) were used for image analysis. The samples were prepared from stock solutions of poly-(*R*)-**1** and poly-(*S*)-**1** (1 mg/mL) in THF that were diluted until 0.01 mg/mL and mixed until get a solution of poly-(*R*)-**1** + poly-(*S*)-**1** in a 50/50 (v/v) ratio. One drop of this mixture (10 μL) was placed either on silicon wafer or on freshly cleaved HOPG (Telstar Instrumat, ZYH grade) and was spin coated at 900 and/or 1800 rpm at rt.

SEM measurements were performed on a LEO-435VP electron microscope equipped with an energy dispersive X-ray (EDX) spectrometer. A drop of a solution of different polymers (0.1 mg/mL) was settled on a silicon wafer chip (Ted Pella, Inc), and allowed to dry at rt for 12 h.

TEM measurements were carried out on Philips CM-12 electron microscope. In this case a drop of the polymer solution (0.1 mg/mL) was placed onto a carbon grid (Ted Pella, Inc).

To investigate the conformational composition of monomers, DFT calculations (B3LYP-6-31G*) were performed. The computational studies were done in Gaussian09 software package. For molecular modelling we used Spartan 08 (MMFF94). As a molecular visualization system we used PyMOL.

2. Synthesis

2.1. Synthesis of monomers M-(*R*)- and M-(*S*)-1

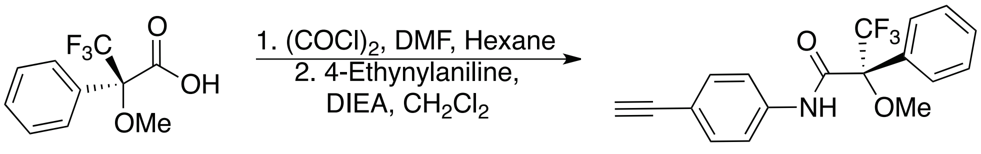

M-(*R*)-**1**

Oxalyl chloride (2.5 mL, 1.4 equiv) was added dropwise to a solution of (*R*)- or (*S*)-α-methoxy-α-(trifluoromethyl)phenylacetic acid (1.00 g, 1.0 equiv) in dry hexane and DMF (300 μL, 1.0 equiv) at 0 ºC under an Ar atm. After 4 h stirring at rt, the reaction mixture was filtered and the solution obtained dried under vacuum to give the acetyl chloride as colorless oil in quantitative yield.

The (*S*)- or (*R*)-α-methoxy-α-(trifluoromethyl)phenylacetyl chloride obtained, (1.07 g, 1.0 equiv) was dissolved in CH_2Cl_2 (40.0 mL) and diisopropyltriethylamine (DIEA, 1.2 mL, 1.6 equiv) and 4-ethynilaniline (500.0 mg, 1.0 equiv) where added to the solution. The reaction mixture was stirred at rt overnight. The residue was diluted with CH_2Cl_2 and the organic solution was washed with HCl 1M, saturated $Na(HCO_3)$ aq solution and saturated NaCl aq solution; then the organic layer was dried over anhyd Na_2SO_4. After filtration, the solution was evaporated and the residue was chromatographed on silica gel with hexane-AcOEt (7/3, v/v) as the eluent [1.30 g M-(*R*)-**1** and 1.28 g M-(*S*)-**1**, 91% and 90 % yield respectively of pure products].

Spectroscopic Data

(R)-*N*-(4-ethynylphenyl)-3,3,3-trifluoromethyl-2-methoxy-2-phenylpropanamide [M-(*R*)-1]:

α_D = +68.2 (c = 10.0 mg/mL, $CHCl_3$). Mp = 87 ºC

^{1}H NMR (300 MHz, $CDCl_3$) δ(ppm): 3.04 (s, 1H), 3.47 (s, 3H), 7.40-7.50 (m, 5H), 7.52-7.57 (m, 4H), 8.61 (broad s, 1H)

^{19}F NMR (282.24 MHz, $CDCl_3$) δ(ppm): - 68.7

^{13}C NMR (75 MHz, $CDCl_3$) δ(ppm): 55.2, 83.1, 118.5, 119.5, 127.7, 128.0, 128.8, 129.0, 129.8, 132.0, 133.0, 137.1, 164.3. HRMS (ESI) m/z calcd for $C_{18}H_{15}F_3N_1O_2$ [M+H]: 334.1010, found: 334.1039.

(S)-*N*-(4-ethynylphenyl)-3,3,3-trifluoro-2-methoxy-2 phenylpropanamide [M-(*S*)-1]:

α_D = -67.7 (c = 10.0 mg/mL, $CHCl_3$). Mp= 88 ºC

^1H NMR (300 MHz, $CDCl_3$) δ(ppm): 3.04 (s, 1H), 3.47 (s, 3H), 7.40-7.50 (m, 5H), 7.52, -7.57 (m, 4H), 8.61 (broad s, 1H).

^{19}F NMR (282.24 MHz, $CDCl_3$) δ(ppm): -68.7.

^{13}C NMR (75 MHz, CDCl3) δ(ppm): 55.2, 83.1, 118.5, 119.5, 127.7, 128.0, 128.8, 129.0, 129.8, 132.0, 133.0, 137.1, 164.3. HRMS (ESI) m/z calcd for $C_{18}H_{15}F_3N_1O_2$ [M^+ H]: 334.1010, found: 334.1049.

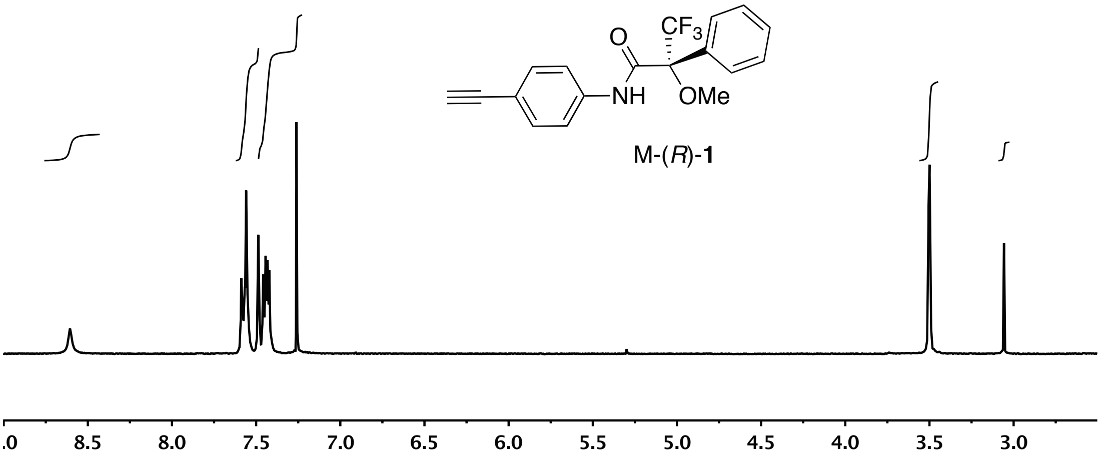

Figure S1: ^1H NMR spectra of M-(*R*)-**1** ($CDCl_3$, 300 MHz).

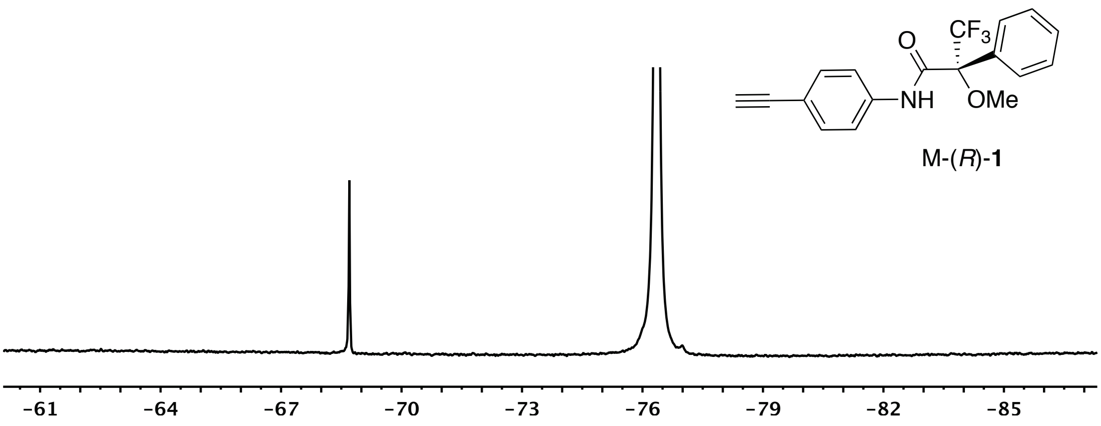

Figure S2: ^{19}F NMR spectra of M-(*R*)-**1** ($CDCl_3$, 282.24 MHz).

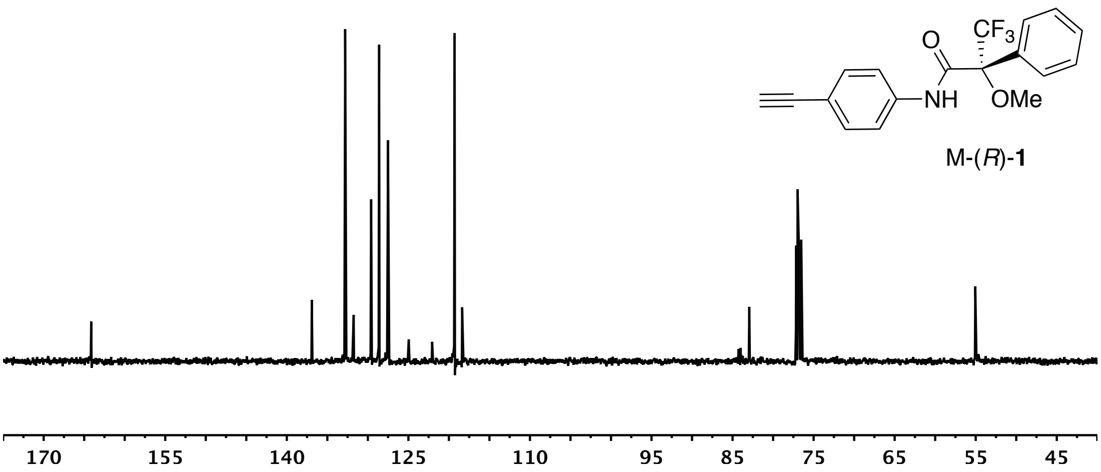

Figure S3: ^{13}C NMR spectra of M-(*R*)-**1** ($CDCl_3$, 75 MHz).

2.2. Synthesis of monomer 2

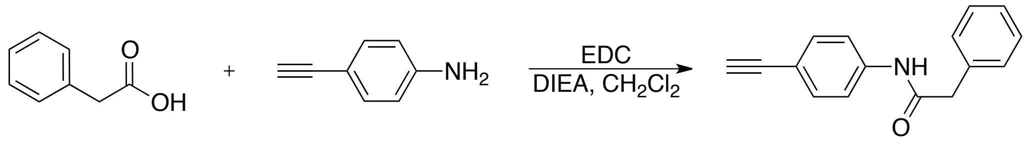

M-**2**

4-Ethynilaniline (0.40 g, 1.0 equiv) was added to a solution of 2-phenylacetic acid (0.50 g, 1.1 equiv) and 1-ethyl-3-(3-dimethylaminopropyl)carbodiimide (EDC) (0.80 g, 1.25 equiv) in CH_2Cl_2 (40.0 mL) and diisopropyltriethylamine (DIEA, 1.2 mL, 2.0 equiv). The reaction mixture was stirred at rt overnight. The residue was diluted with CH_2Cl_2 and the organic solution was washed with HCl 1M, saturated $Na(HCO_3)$ aq solution and saturated NaCl aq solution; then the organic layer was dried over anhyd Na_2SO_4. After filtration, the solution was evaporated and the residue was chromatographed on silicagel with hexane-AcOEt (7/3, v/v) as the eluent yielding 94% (0.74 g) of pure product M-**2**.

Spectroscopic Data

***N*-(4-ethynylphenyl)-2-phenylacetamide (2):**

^{1}H NMR (300 MHz, $CDCl_3$) δ(ppm): 3.03 (s, 1H), 3.72 (s, 2H), 7.3-7.5 (m, 10H).

^{13}C NMR (75 MHz, $CDCl_3$) δ(ppm): 44.8, 76.9, 83.9, 117.8, 119.3, 127.8, 129.3, 129.5, 132.9, 134.1, 138.0, 169.2.

HRMS (ESI) m/z calc. for $C_{16}H_{15}NO$ [M + H]: 236.1075, found: 236.1066.

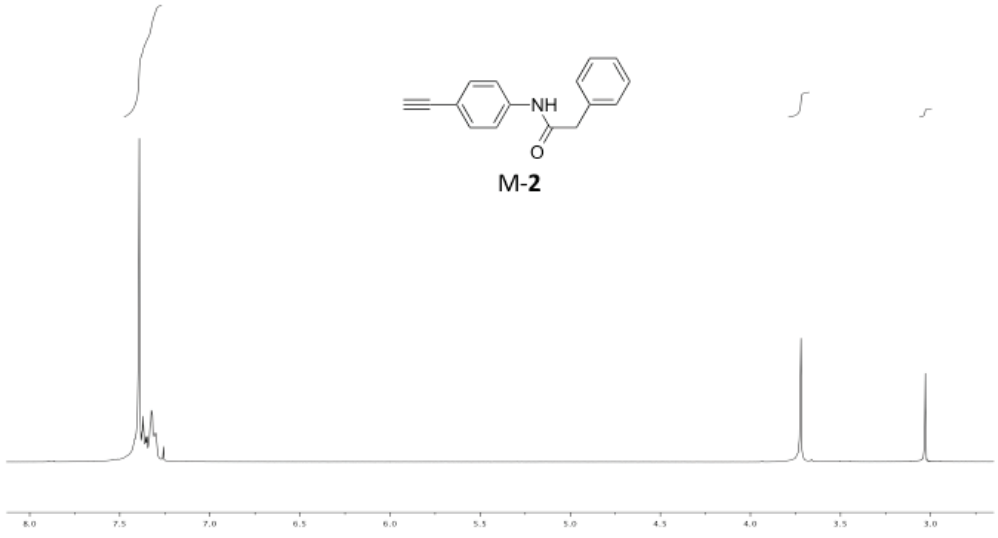

Figure S4: ^{1}H NMR spectra of monomer-**2** ($CDCl_3$, 300 MHz)

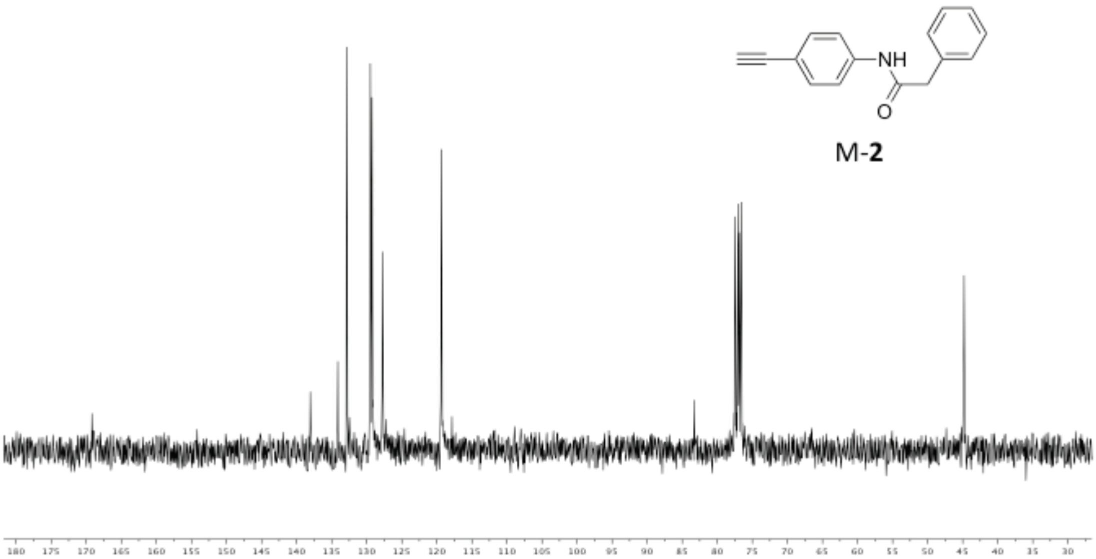

Figure S5: ^{13}C NMR spectra of monomer-**2** ($CDCl_3$, 75 MHz).

2.3. Synthesis of polymers poly-(*R*)-1 and poly-(*S*)-1

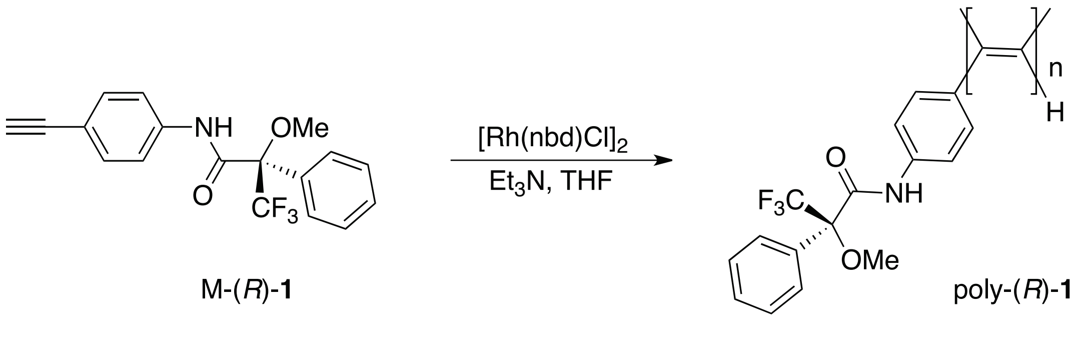

The reaction flask (sealed ampoule) was dried under vacuum and Ar flushed for three times before M-(*R*)-**1** (200.0 mg, 0.5 M) was added as a solid. Then, the flask was evacuated on a vacuum line and flushed with dry Ar (three times). Dry THF (1000 μL) and Et_3N (10 μL, 0.05 M) were added via a syringe. A solution of rhodium norbornadiene chloride dimmer (2.7 mg), $[Rh(nbd)Cl]_2$, in THF (200 μL) was added at 30° C. The reaction mixture was stirred at 30°C for 2 h. Then, the resulting polymer (poly-(*R*)-**1**) was diluted in THF and precipitated in a large amount of MeOH, centrifuged (2 times) and reprecipitated in hexane and centrifuged again (178.2 mg, 89% yield).

Analogous procedure was employed to prepare poly-(*S*)-**1**.

Spectroscopic Data

Poly-(*R*)-1 :

α_D = -379.3 (c = 7.5 mg/mL, CHCl3).

^{1}H NMR (750 MHz, CD_2Cl_2) δ(ppm): 3.40 (s, br, 3H), 5.78 (s, br, 1H), 6.47 (s, broad, 2H), 7.20 – 7.50 (d, br, 7H), 8.60 (broad , 1H).

^{19}F NMR (282.24 MHz, CD_2Cl_2) δ(ppm): - 68.0.

^{13}C NMR (125 MHz, CD_2Cl_2) δ(ppm): 54.9, 89.3, 119.6, 123.0, 125.2, 127.8, 128.6, 132.2, 135.4, 138.4, 162.9.

Molecular weight (Mn): 102000; Mw/Mp = 2.2. [THF as eluent at a flow rate of 1mL/min and a concentration of 2g/L using narrow polystyrene standards (PSS) as calibrants].

Poly-(*S*)-1 :

αD = +353.1 (c = 7.5 mg/mL, $CHCl_3$).

^{1}H NMR (750 MHz, CD_2Cl_2) δ(ppm): 3.40 (s, br, 3H), 5.78 (s, br, 1H), 6.47 (s, broad, 2H), 7.20 – 7.50 (d, br, 7H), 8.60 (broad s, 1H).

^{19}F NMR (282.24 MHz, CD_2Cl_2) δ(ppm): - 68.0.

^{13}C NMR (125 MHz, CD_2Cl_2) δ(ppm): 54.9, 89.3, 119.6, 123.0, 125.2, 127.8, 128.6, 132.2, 135.4, 138.4, 162.9.

Molecular weight (Mn): 60100; Mw/Mp = 1.4. (THF as eluent at a flow rate of 1mL/min and a concentration of 2 g/L using narrow polystyrene standards (PSS) as calibrants).

The *cis-transoidal* stereoregularity of poly-(*R*)-**1** was determined by ^{1}H NMR spectroscopy, where the vinyl proton resonates at 5.78 ppm (Figure S6), and Raman Resonances (Figure S10). The peak at 1567 cm^{-1} is assigned to C=C bond stretching in the *cis* polyacetylene and overlaps with that of the phenyl ring. The peak at 1336 cm^{-1} is assigned to the *cis* C-C bond coupled with the single bond connecting the main chain and the phenyl ring. The peak at 1003 cm^{-1} is assigned to the C-H bond deformation of the *cis* form. Identical NMR and Raman spectra were obtained for the enantiomeric polymer, poly-(*S*)-**1**.

Raman spectra were performed in a confocal Renishaw Raman spectrometer (Invia Reflex model) equipped by 785 nm diode laser, Ion Ar 514 nm. (350 mW, 1.064 nm excitation wavelength). NMR experiments were carried out in a Varian Inova 750 spectrometer, 17.61 T magnetic field.

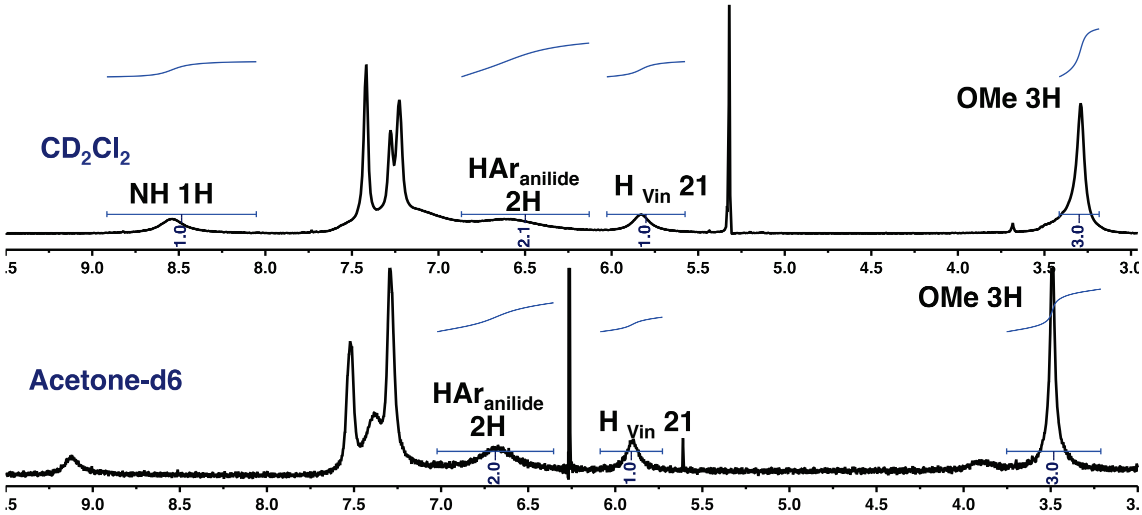

Figure S6: ^{1}H NMR spectra of poly-(*R*)-**1** (CD_2Cl_2 and Acetone-d6, 750 MHz).

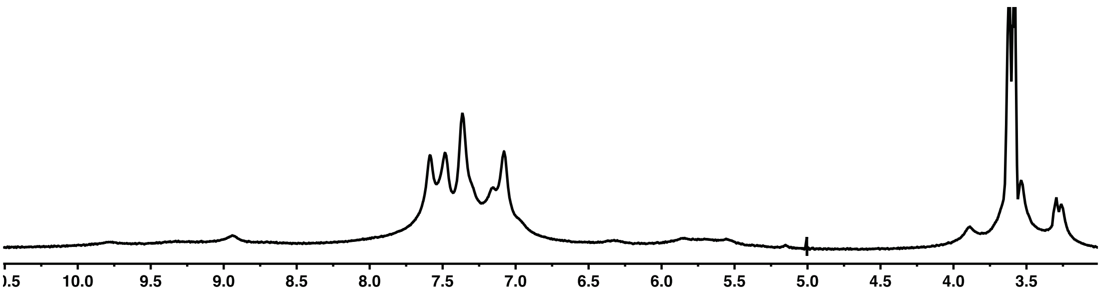

Figure S7: ^{1}H NMR spectra of poly-(*R*)-**1** (THF-d8, 750 MHz).

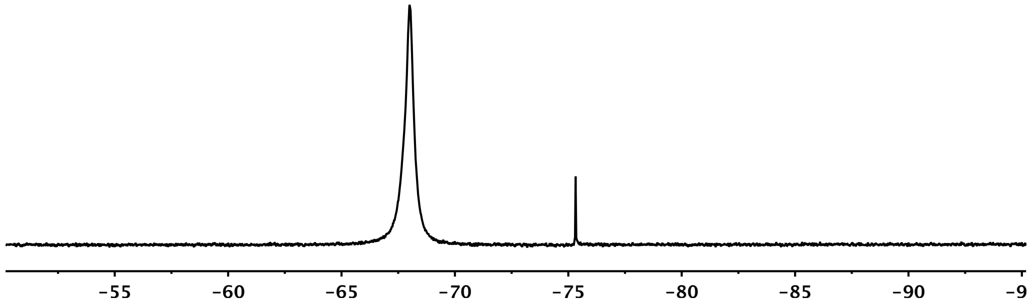

Figure S8: ^{19}F NMR spectra of poly-(*R*)-**1** (CD_2Cl_2, 282.24 MHz).

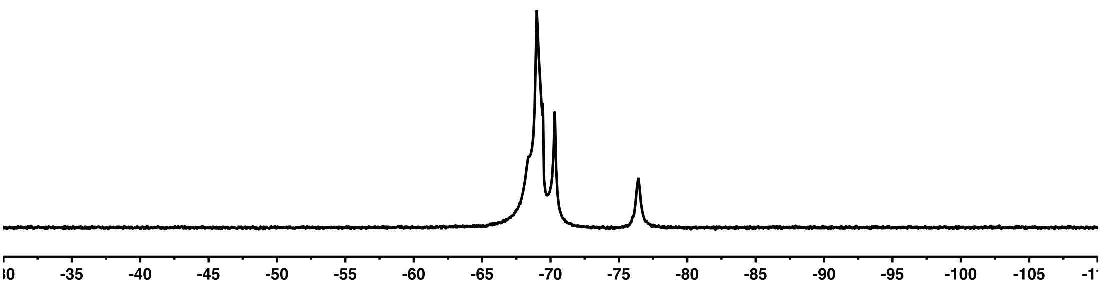

Figure S9: ^{19}F NMR spectra of poly-(*R*)-**1** ((THF-d8, 282.24 MHz).

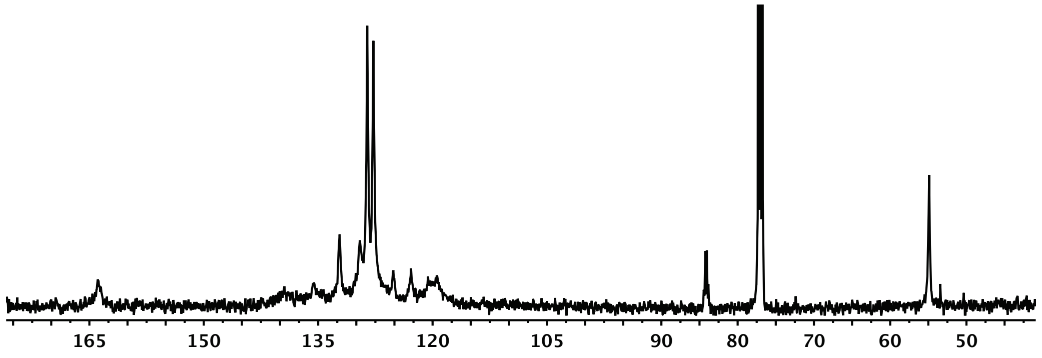

Figure S10: ^{13}C NMR spectra of poly-(*R*)-**1** (CD_2Cl_2, 125 MHz).

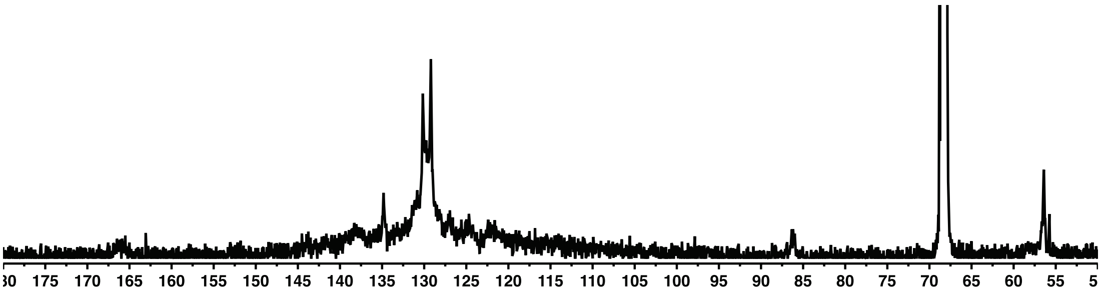

Figure S11: ^{13}C NMR spectra of poly-(*R*)-**1** (THF-d8, 125 MHz).

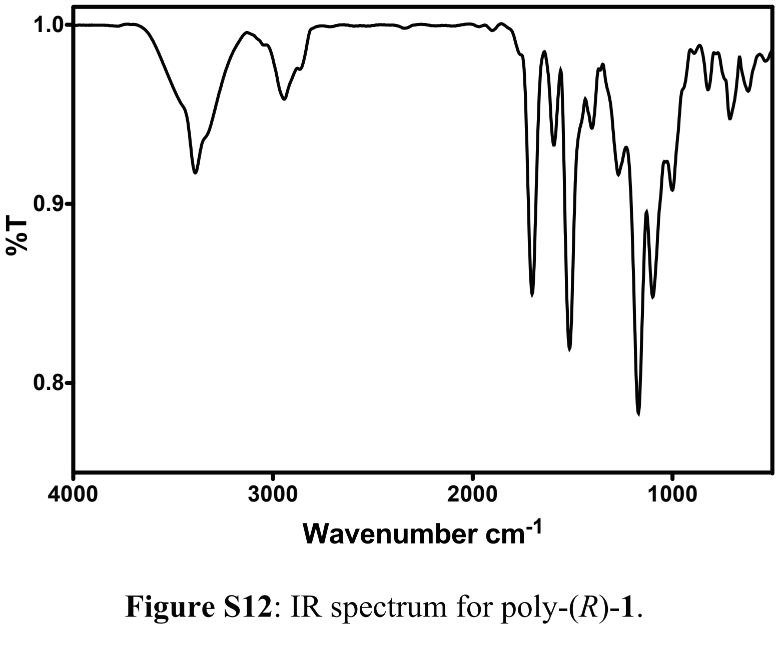

Figure S12: IR spectrum for poly-(*R*)-**1**.

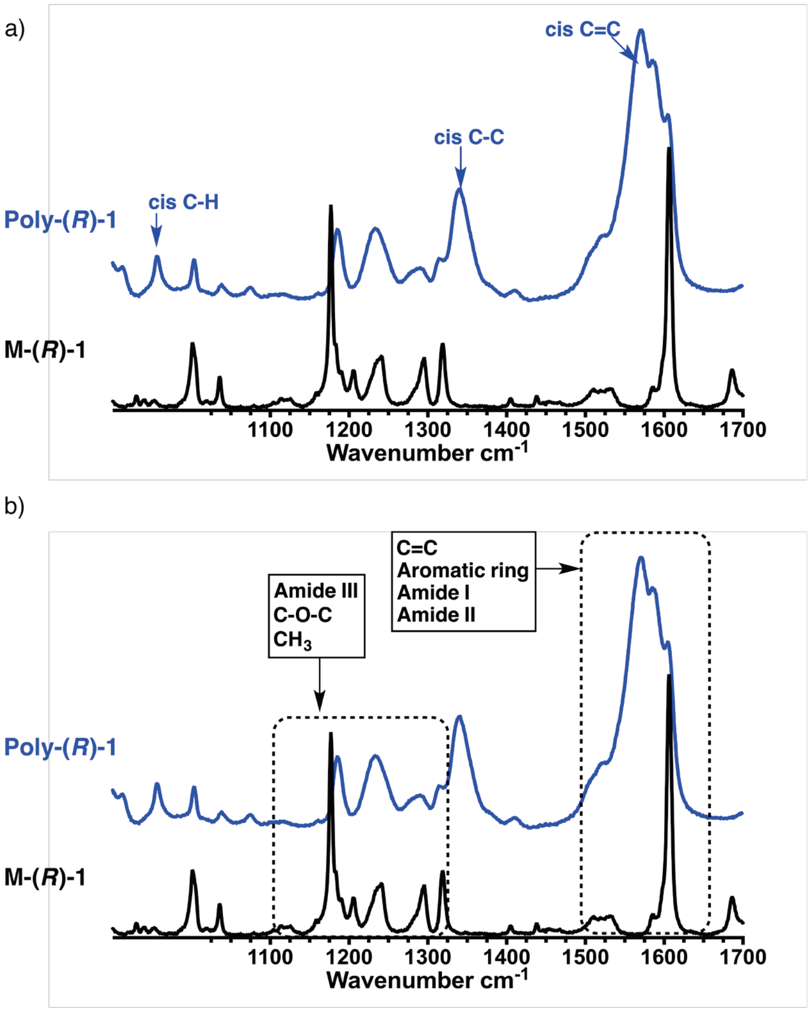

Figure S13: Assigned Raman spectrum for poly-(*R*)-1.[1]

[1] a) C. S. S. R. Kumar (Ed.), *Raman spectroscopy for Nanomaterials Characterization* (Springer), 2012. b) R. Tuma, *J. Raman Spectroc.* 2005, **36**, 307-319.

2.4. Synthesis of polymers poly[(*R*)-1-co-2] and poly[(*S*)-1-co-2]

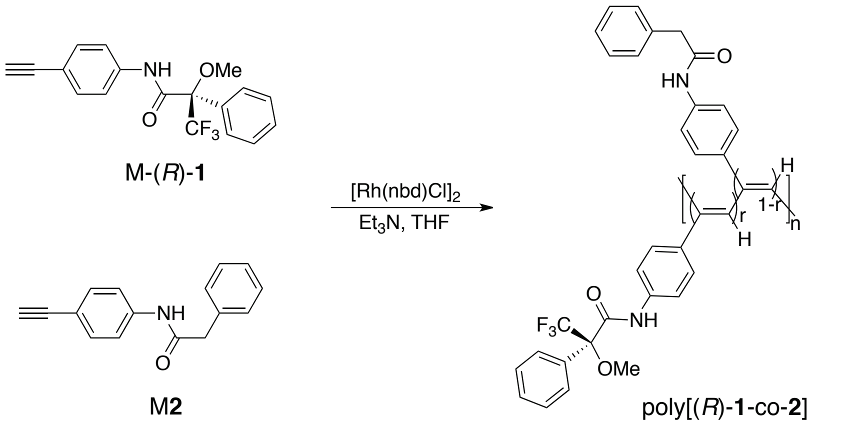

The reaction flask (sealed ampoule) was dried under vacuum and Ar flushed for three times before monomer-**1** and monomer-**2** (see table below) were added as a solid. Then, the flask was evacuated on a vacuum line and flushed with dry Ar (three times). Dry THF (600 µL) and Et_3N (4 µL, 0.05 M) were added via a syringe. A solution of rhodium norbornadiene chloride dimmer (1.4 mg), $[Rh(nbd)Cl]_2$, in THF (100 µL) was added at 30º C. The reaction mixture was stirred at 30ºC for 1 h. Then, the resulting polymer poly[(*R*)-**1**-co-**2**] was diluted in THF and precipitated in a large amount of MeOH, centrifuged (2 times) and reprecipitated in hexane and centrifuged again.
Analogous procedure was employed to prepare poly-[(*S*)-**1**-co-**2**].

Copolymer	M-(*R*)-**1** (mg)	M-(*R*)-**1** (mmol)	M**2** (mg)	M**2** (mmol)	Yield (%)
Poly-[(*R*)-$\mathbf{1}_{90}$-co-$\mathbf{2}_{10}$]	90.0	0.270	7.1	0.030	92
Poly-[(*R*)-$\mathbf{1}_{80}$-co-$\mathbf{2}_{20}$]	80.0	0.240	14.1	0.060	90
Poly-[(*R*)-$\mathbf{1}_{70}$-co-$\mathbf{2}_{30}$]	70.0	0.210	21.2	0.090	91
Poly-[(*R*)-$\mathbf{1}_{60}$-co-$\mathbf{2}_{40}$]	60.0	0.180	28.2	0.120	89
Poly-[(*R*)-$\mathbf{1}_{50}$-co-$\mathbf{2}_{50}$]	50.0	0.150	35.3	0.150	90
Poly-[(*R*)-$\mathbf{1}_{40}$-co-$\mathbf{2}_{60}$]	40.0	0.120	42.4	0.180	87
Poly-[(*R*)-$\mathbf{1}_{20}$-co-$\mathbf{2}_{80}$]	20.0	0.060	56.5	0.240	90

Table S1: monomer quantities used in the copolimerizations and product yields.

Spectroscopic Data

Copolymer	M_n	M_w	M_p	M_z	PDI
Poly-[(*R*)-$\mathbf{1}_{90}$-co-$\mathbf{2}_{10}$]	2378613	3659333	4689406	4915426	1.54
Poly-[(*R*)-$\mathbf{1}_{80}$-co-$\mathbf{2}_{20}$]	1001216	2155480	3564717	3476061	2.15
Poly-[(*R*)-$\mathbf{1}_{70}$-co-$\mathbf{2}_{30}$]	780281	1819557	1872348	3148338	2.33
Poly-[(*R*)-$\mathbf{1}_{60}$-co-$\mathbf{2}_{40}$]	577203	1411851	1304386	2836259	2.68
Poly-[(*R*)-$\mathbf{1}_{50}$-co-$\mathbf{2}_{50}$]	366659	1091332	1097729	2349805	2.98
Poly-[(*R*)-$\mathbf{1}_{40}$-co-$\mathbf{2}_{60}$]	538952	1383728	1666714	2475387	2.57
Poly-[(*R*)-$\mathbf{1}_{20}$-co-$\mathbf{2}_{80}$]	180564	530383	621794	1223633	2.94

Table S2: Copolymer molecular weights and PDI obtained by SEC.

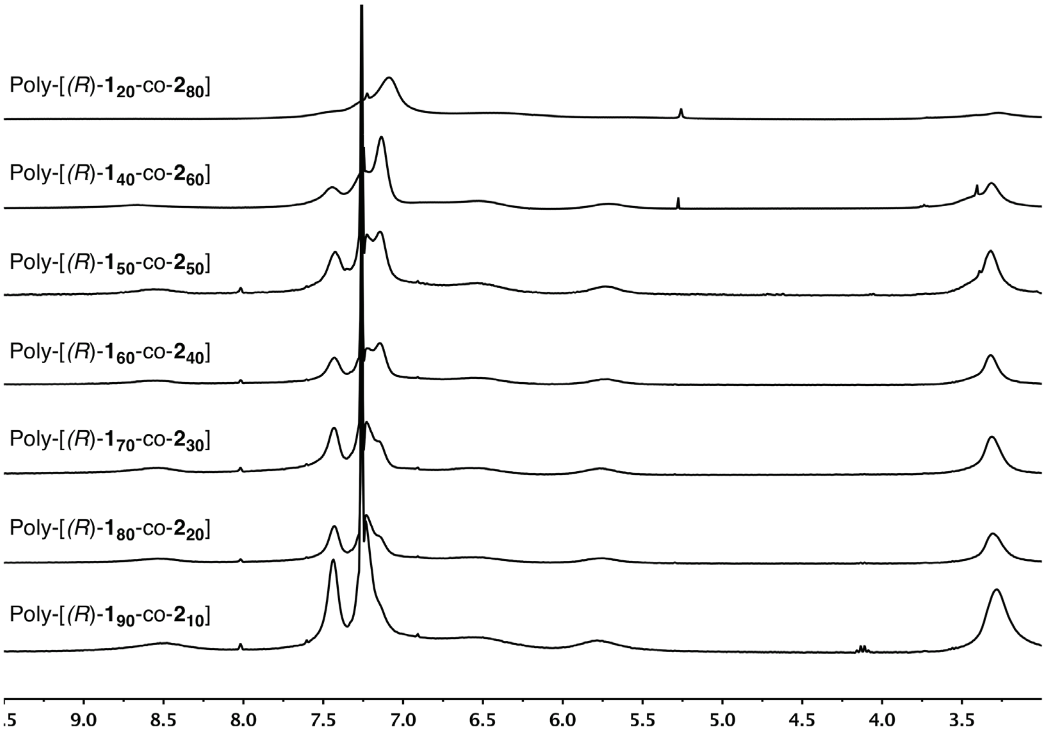

Figure S14: ^{1}H NMR spectra of Poly-[(*R*)-$\mathbf{1}_r$-co-$\mathbf{2}_{1-r}$] ($CDCl_3$, 300 MHz).

a)

b)

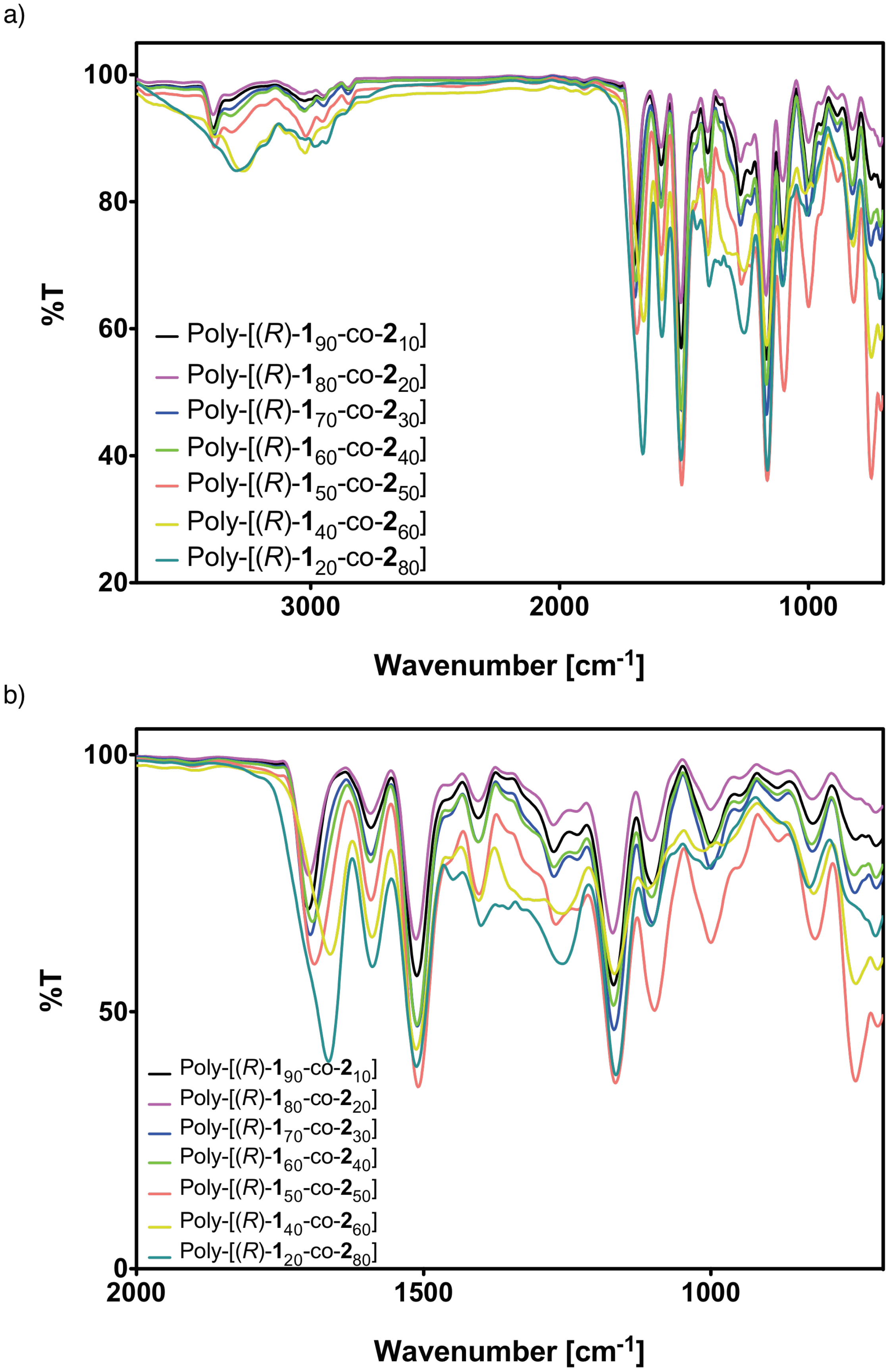

Figure S15: IR spectra of poly-[(*R*)-$\mathbf{1}_{r}$-co-$\mathbf{2}_{1-r}$]

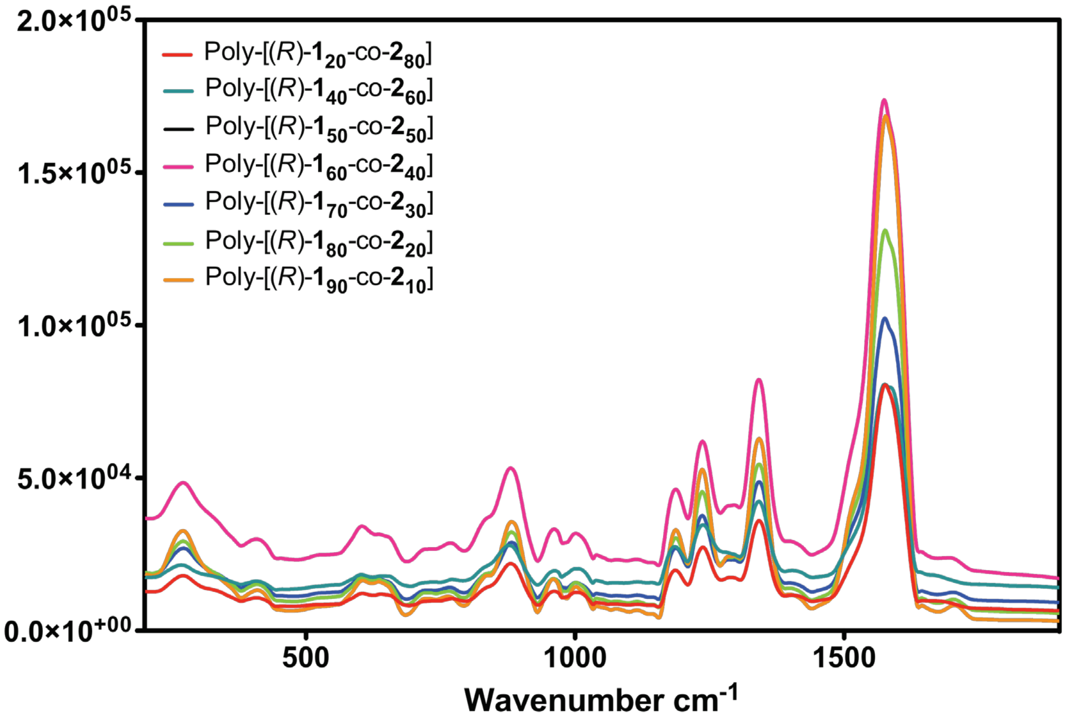

Figure S16: Raman spectra of poly-[(*R*)-$\mathbf{1}_{\mathbf{r}}$-co-$\mathbf{2}_{\mathbf{1\text{-}r}}$]

3. Determination of the monomer reactivity ratios in copolymerizations

To estimate the monomer reactivity ratios in the copolymerization of poly[(*R*)-**1**-*co*-**2**] (r1 and r2), the copolymerization of M-(*R*)-**1** with M-**2** at varying monomer feed ratios was carried out and terminated at low conversions by adding, in both cases, a solution of HCl 0,05 M in MeOH (5 mL) 10 seconds after the catalyst addition. Next, a basic solution of NaOH 0,05 M in MeOH (5 mL) was added followed by MeOH (10 mL) containing a small amount of conc HCl. The resulting poly[(*R*)-**1**-*co*-**2**] copolymers were collected by centrifugation, and dried in vacuo at room temperature overnight. The copolymer compositions were determined from their qualitative ^{13}C NMR spectra (^{13}C inversed gate NMR spectra, Figure S14) and the results are summarized in Table S3. The copolymer compositions are almost similar to the corresponding monomer feed ratios. For the determination of r1 and r2, the Kelen-Tüdös method was used. In the Kelen-Tüdös method, the following equation is empoyed:

$$\eta = \left(r_1 + \frac{r_7(r_8)}{\alpha}\right)\xi - \frac{r_7(r_8)}{\alpha}$$

Where η and ξ are expressed as $G/(a+H)$ and $H/(\alpha + H)$, respectively, by using G and H, which are defined as $G = F(f\text{–}1)/f$ and F^2/f, respectively, with F and f representing themolar ratios of monomers in the feed and the molar ratios in the copolymers. α is a parameter defined as $\alpha = (F_{min} \bullet F_{max})^{0,5}$, where F_{min} and F_{max} correspond to the lowest and highest values of F, respectively. A plot of η versus ξ affords a straight line (Figure S15. The intercept at ξ = 0 and ξ = 1 gives –r2/α and r1, respectively. The estimated monomer reactivity ratios for M-(*R*)-**1**/M-**2** copolymer are r1 = 1.1508 and r2 = 1.16040 The products of the monomer reactivity ratios for the copolymerizations of M-(*R*)-**1** with M-**2** are 1.1508 and 1.1604, respectively, suggesting that the monomer distributions in the copolymers are mostly random.

	Monomer **2**	Polymer	
Run	*F*	Yield (%) [a]	*f* [b]
Poly(1_{80}-*co*-3_{20})	0.2	51	0.18
Poly(1_{60}-*co*-3_{40})	0.4	48	0.40
Poly(1_{40}-*co*-3_{60})	0.6	47	0.55
Poly(1_{20}-*co*-3_{80})	0.8	50	0.80

[a] Insoluble fraction in MeOH. [b] Determined by integration in the ^{13}C NMR spectra of the copolymers.

Table S3: Monomer-**2** feed in the copolymerizations. Copolymers yield and M-**2** ratio in the different copolymers.

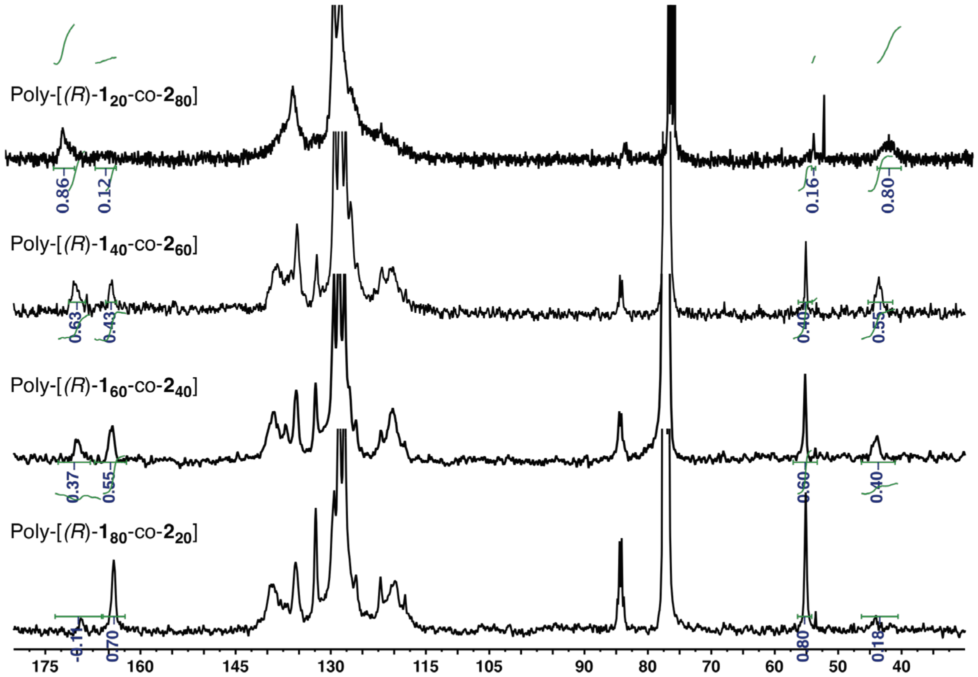

Figure S17: ^{13}C NMR – inversed gate spectra of poly-[(*R*)-**1**$_r$-co-**2**$_{1-r}$] (CDCl$_3$, 125 MHz).

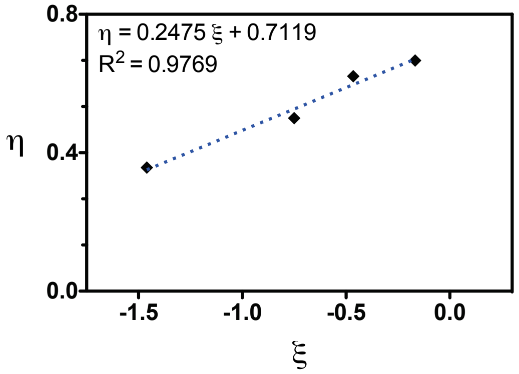

Figure S18: Representation of η *vs.* ξ.

4. Poly-(*R*)-1 + Poly-(*S*)-1 CD and UV-Vis

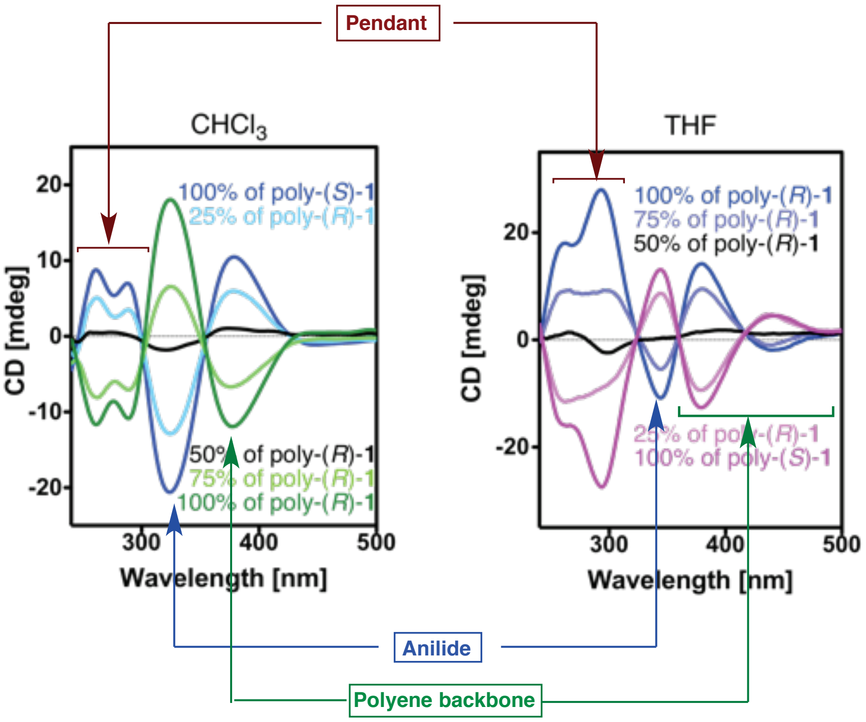

Figure S19: Assigned CD spectra of poly-(*R*)-**1** + poly-(*S*)-**1** at different ratios in $CHCl_3$ and THF.[2]

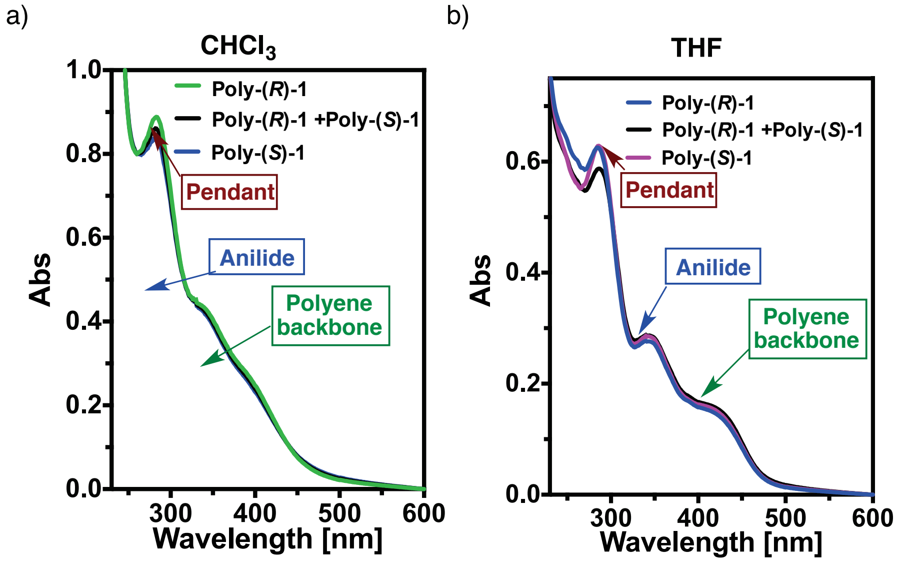

Figure S20: Assigned UV-Vis spectra of poly-(*R*)-**1**, poly-(*S*)-**1** and poly-(*R*)-**1** + poly-(*S*)-**1** in $CHCl_3$ and THF.[3]

[2] N. Berova, K. Nakanishi, R. W. Woody (Eds.), *Circular Dichroism: Principles and Applications, 2nd Ed.*, Wiley-VCH, Weiheim, 2000.
[3] H.H. Perkampus, *UV-VIS Atlas of Organic Compounds* (2nd Ed), Wiley-VCH, Weiheim, 1992.

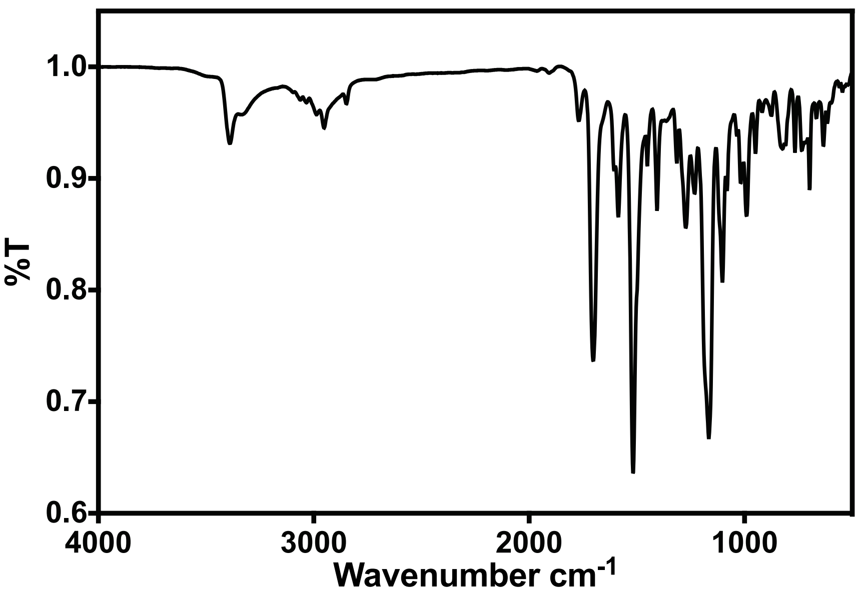

Figure S21: IR of poly-(*R*)-**1** + poly-(*S*)-**1** in THF.

5. Poly-[(*R*)-1-co-2] and Poly-[(*S*)-1-co-2] CD and UV-Vis

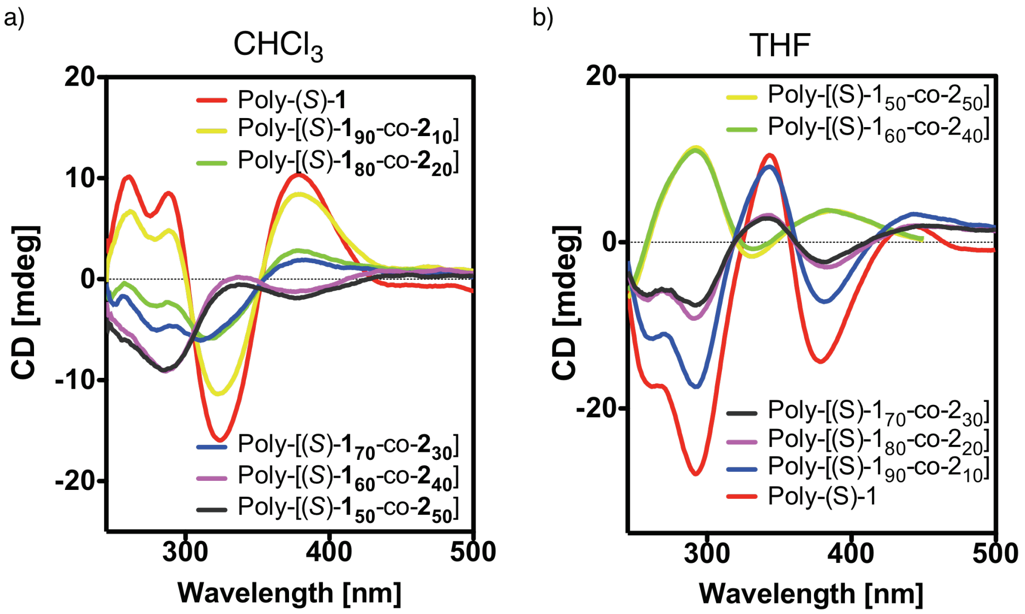

Figure S 22: CD spectra of poly-[(*R*)-**1**$_r$-*co*-**2**$_{1\text{-}r}$] in $CHCl_3$ and THF.

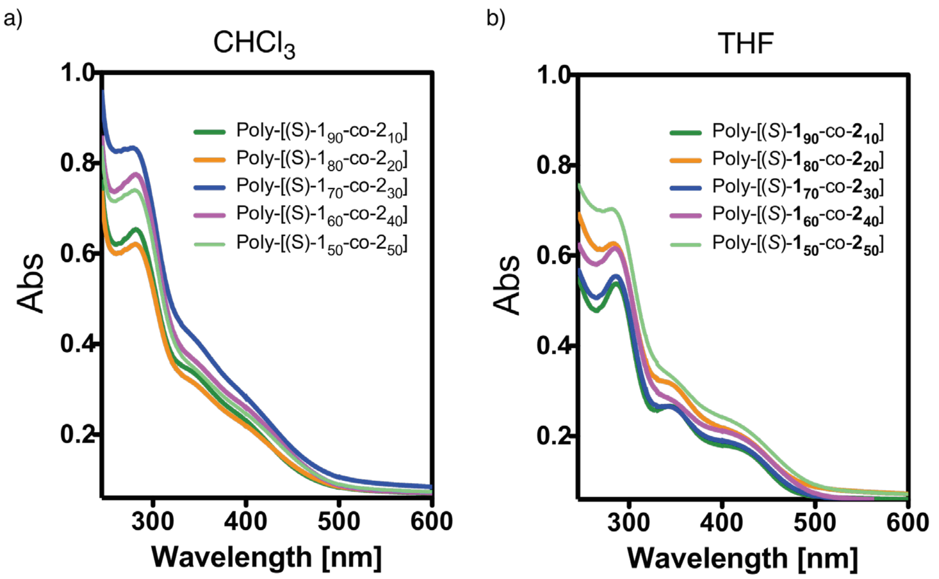

Figure S23: UV-Vis spectra of poly-[(*R*)-**1**$_r$-*co*-**2**$_{1\text{-}r}$] in $CHCl_3$ and THF.

6. Poly-(*R*)-1 aggregation studies by Dynamic Light Scattering (DLS) in different solvents

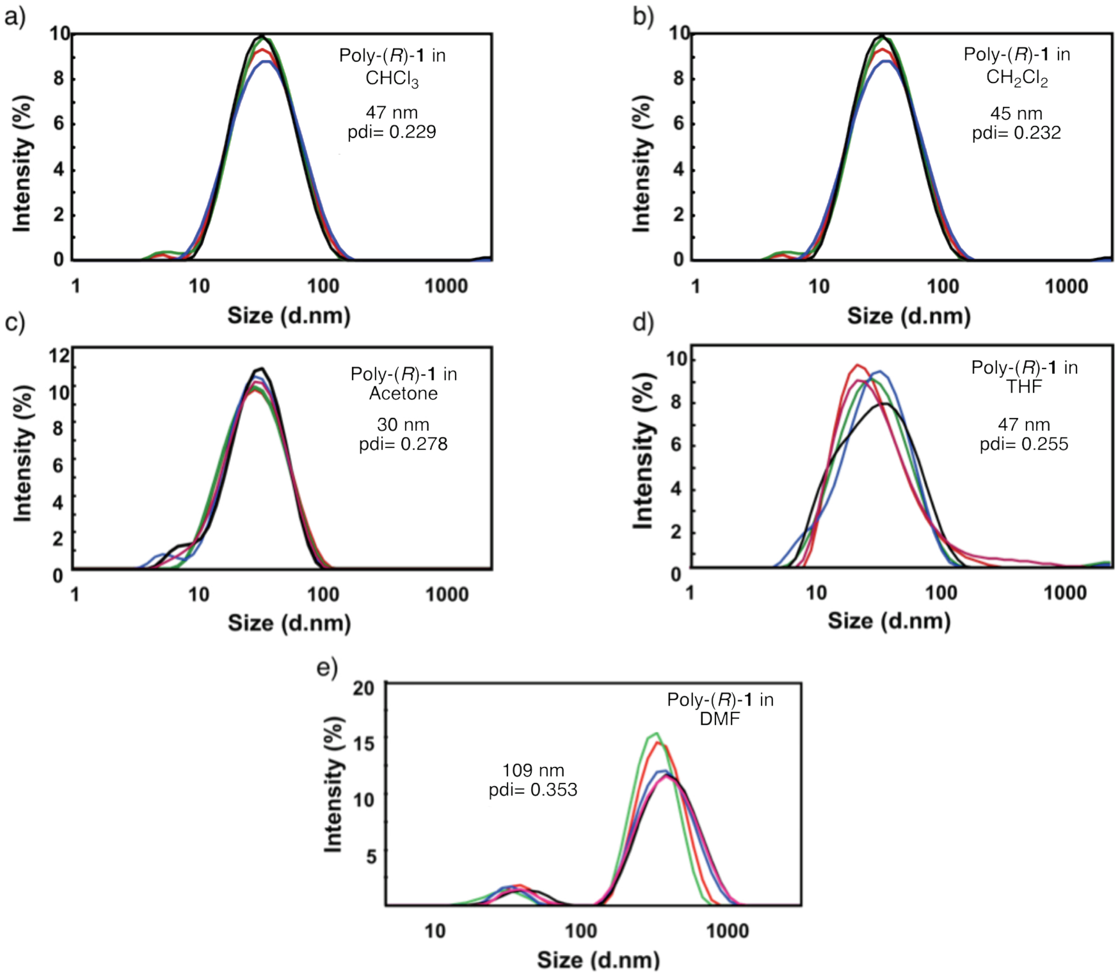

Figure S 24: DLS measurements of poly-(*R*)-**1** in different solvents at 0.2 mg/mL.

7. Poly-(*R*)-1 aggregation studies by Scanning Electron Microscopy (SEM) in different solvents.

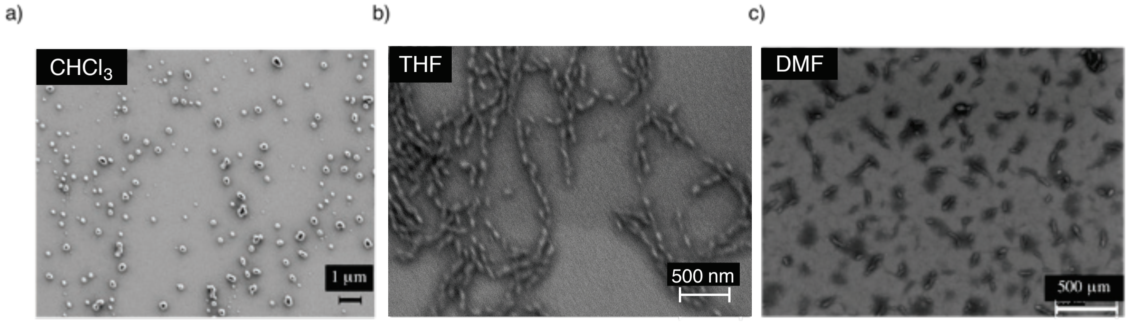

Figure S25: SEM images of poly-(*R*)-**1** in different solvents at 0.1mg/mL.

8. Poly-(*R*)-1 + Poly-(*S*)-1 aggregation studies by Dynamic Light Scattering (DLS) in different solvents

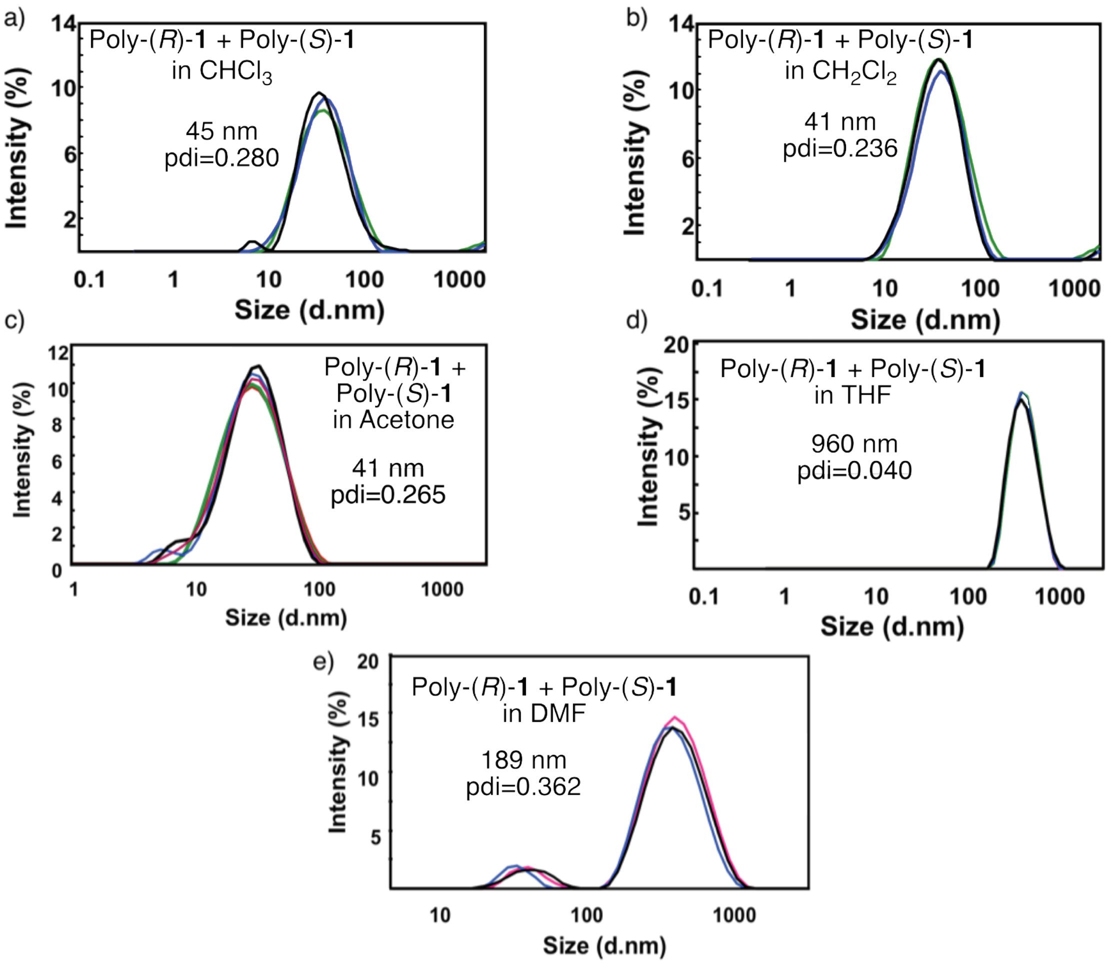

Figure S26: Aggregation studies by DLS of poly-(*R*)-**1** + poly-(*S*)-**1** in different solvents at 0.1mg/mL concentration.

9. Poly-(*R*)-1 + Poly-(*S*)-1 aggregation studies by Dynamic Light Scattering (DLS) in THF

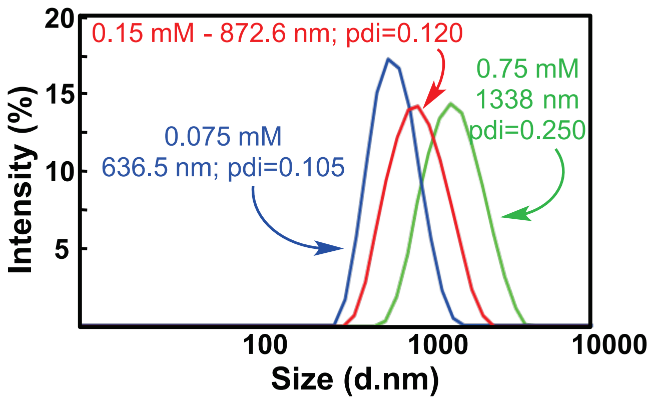

Figure S27: DLS measurements of poly-(*R*)-1 + poly-(*S*)-1 50% / 50% in THF at different final concentrations.

Ratio poly-(*R*)-**1**	Z-size / nm	pdi
100.0	45.65	0.371
99.5	197.70	0.670
99.0	344.12	0.419
98.0	420.90	0.324
95.0	617.40	0.212
90.0	707.70	0.157
80.0	891.30	0.154
70.0	855.50	0.135
60.0	887.20	0.110
50.0	873.70	0.133

Table S4: DLS measurements of poly-(*R*)-**1** + poly-(*S*)-**1** in THF at 0.1mg/mL.

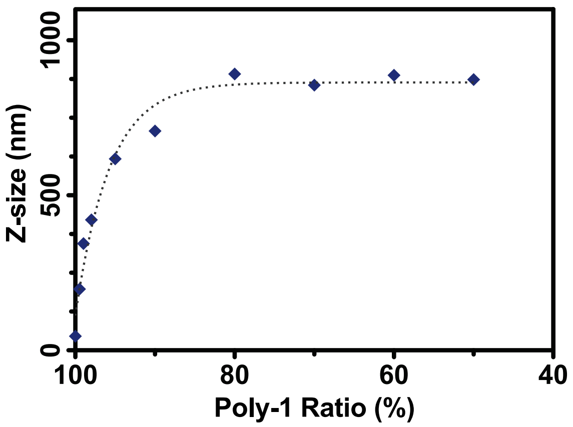

Figure S28: Graphic representation of DLS measurements of poly-(*R*)-**1** + poly-(*S*)-**1** in THF at 0.1mg/mL.

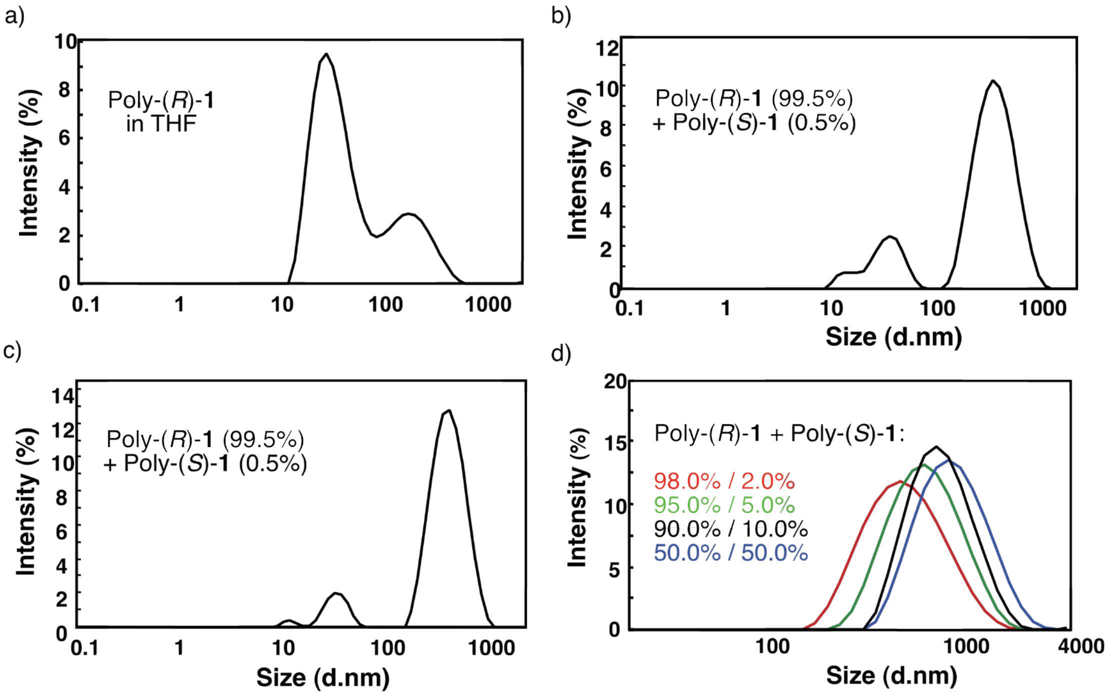

Figure S29: DLS measurements of poly-(*R*)-**1** + poly-(*S*)-**1** in THF at 0.1mg/mL concentration of parent solutions.

10. Reversibility of the stereocomplex formation

The predominant operative factor in the stereocomplex disruption depends on the type of external stimuli employed.
—With increasing temperature (Figure S30) or addition of small amount of a solvent that disrupts hydrogen bonding (*e.g.* MeOH; Figures S31-32), the operative factor is the dissociation of intermolecular hydrogen bonds (no *cis-trans* isomerization takes place).
—With increasing amount of a solvent with poor donor capacity (*e.g.* $CHCl_3$; Figure S33), the operative factor is the tuning of the *cis-trans* amide bond equilibrium that provokes the sifting from a 2/1 to a 3/1 helix where intermolecular hydrogen bonding cannot take place.

10.1. Increasing temperature

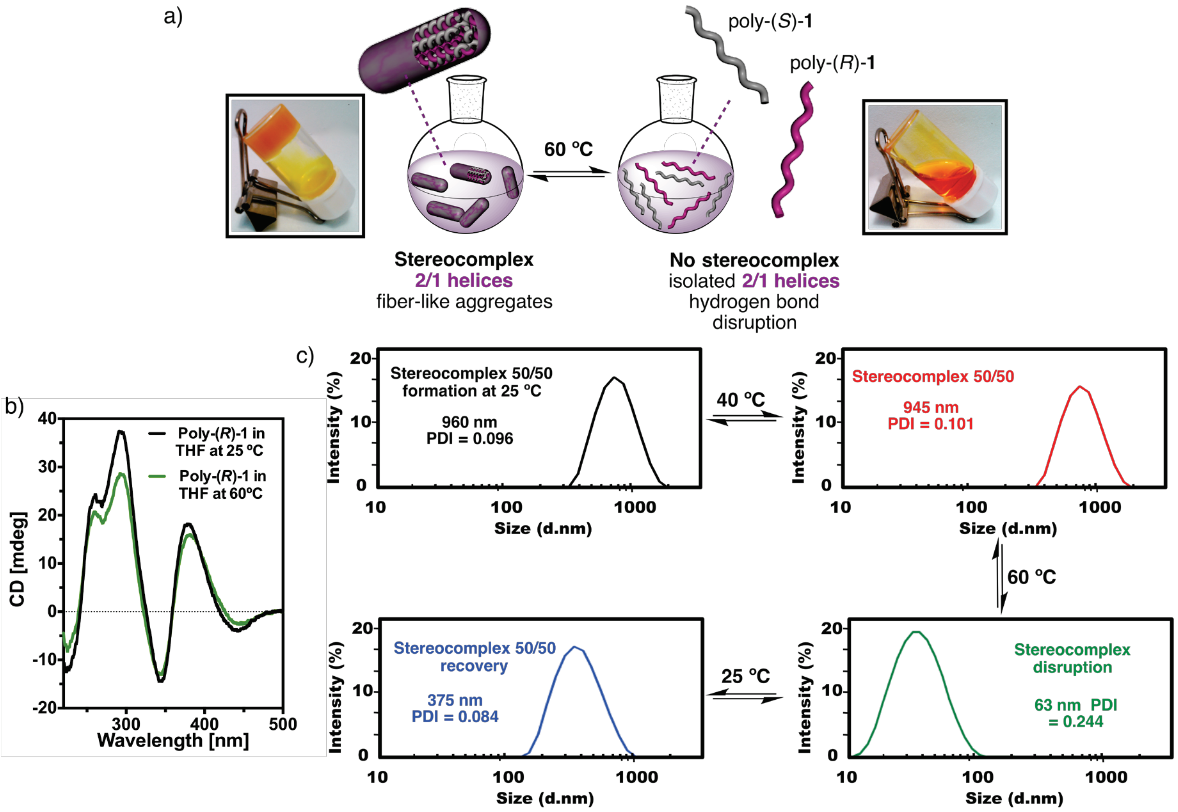

Figure S30: a) Graphical scheme showing the stereocomplex disruption through intermolecular hydrogen bonding disruption by temperature. b) CD spectra of poly-(*R*)-**1** in THF at 25 ºC and 60 ºC showing almost identical CD signatures (2/1 helices, *cis*-amide bonds). c) DLS traces of the sequential heating and cooling of THF solutions of the stereocomplex [50/50 poly-(*R*)-**1**/poly-(*S*)-**1**] show that supramolecular hydrogen bonds among *cis* amides of enantiomeric polymers are disrupted at 60 ºC in a reversible way. The stereocomplex is recovered after cooling down of the sample.

10.2. Addition of MeOH

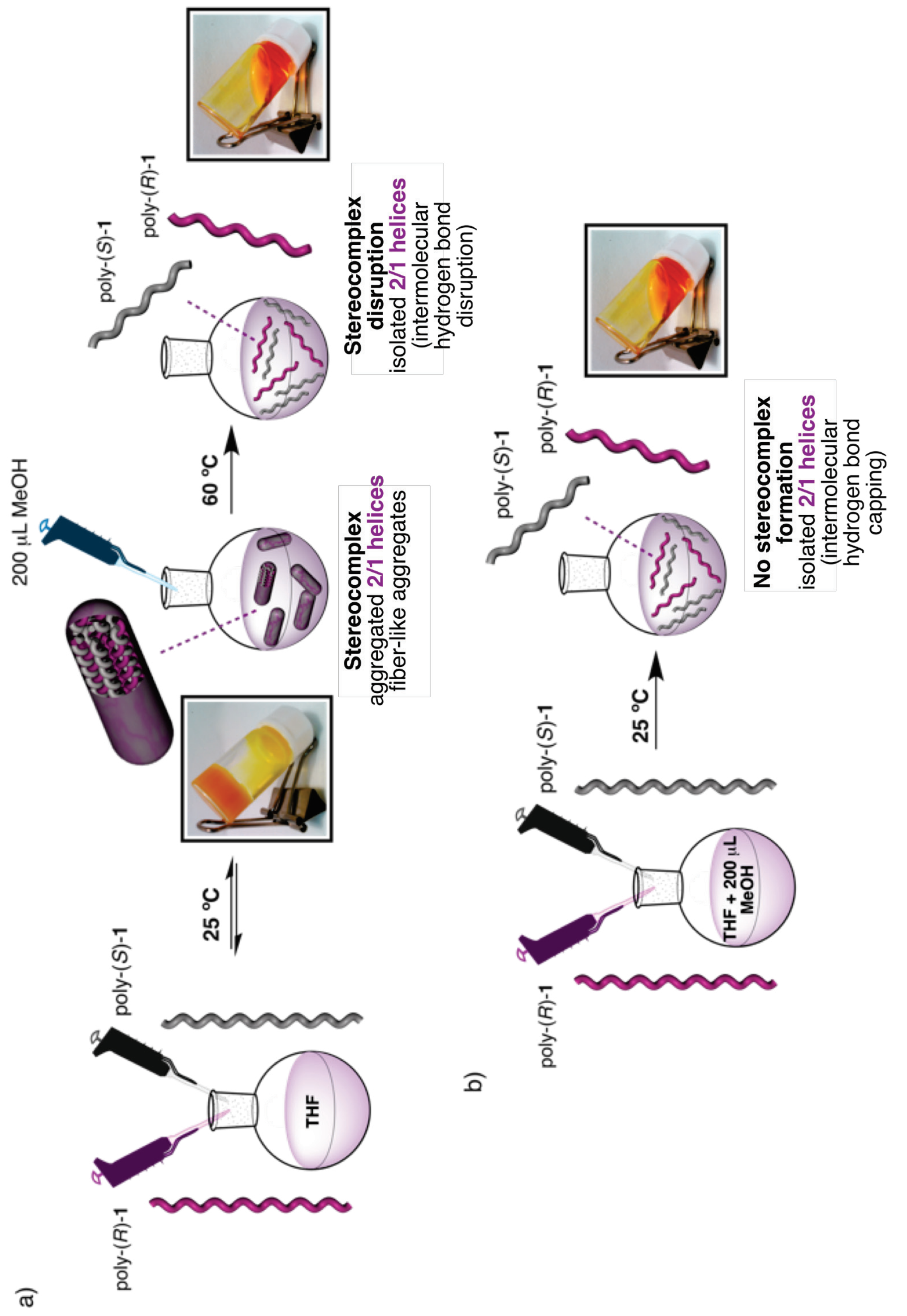

Figure S31: Graphical scheme of 50/50 stereocomplex disruption by the addition of 200 μL of MeOH to an 800 μL solution of 50/50 poly-(*R*)-**1**/poly-(*S*)-**1** in THF (final concn= 0.1 mg/mL) a) after the stereocomplex formation and b) previous to the stereocomplex formation.

The control of the helicity and elongation of poly-(*R*)-**1** based on the manipulation of two adjacent bonds at the pendants by either polarity or donor capacity of the solvent has been described.[4] As a result, the polymer presents different states in different solvents, i.e. poly-(*R*)-**1** presents as external helix: a *M*(3/1, *c*-*c*) helix in MeOH; a *P*(2/1, *c*-*t*) helix in THF; and a *P*(3/1, *c*-*c*) in $CHCl_3$ —opposite helicities for poly-(*S*)-**1** —. The disruption of the stereocomplex [*P*(2/1, *c*-*t*) for poly-(*R*)-**1** and *M*(2/1, *c*-*t*) for poly-(*S*)-**1** in THF] by the addition of certain amounts of MeOH (i.e. 200 μL MeOH to 800 μL of stereocomplex in THF; 0.1 mg/mL) (Figures S31 and S32) does not alter the helical structure of the polymer —the *P*(2/1, *c*-*t*) and *M*(2/1, *c*-*t*) helices are maintained, even at higher amounts of MeOH such as 300 μL MeOH in 700 μL THF—.
Figure S32a shows CD spectra of poly-(*R*)-**1** demonstrating that those MeOH/THF ratios do not affect the initial helical conformation in THF. Thus, MeOH in those amounts only provoke the intermolecular hydrogen bonding rupture (that is the cause of the stereocomplex disruption).

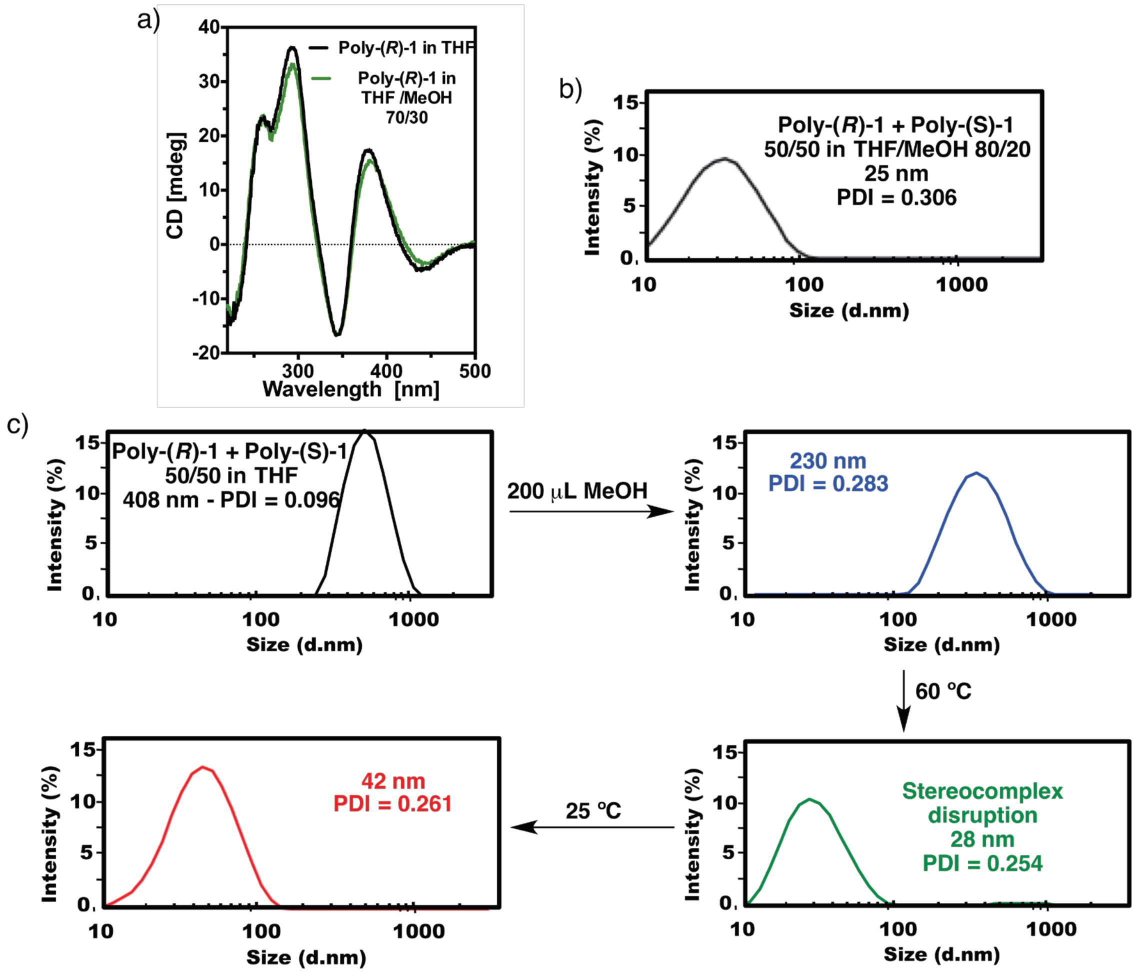

Figure S32: a) CD spectra of poly-(*R*)-**1** (0.1mg/mL) in THF and in THF/MeOH 70/30 (the helical structure is not altered). b) DLS trace of poly-(*R*)-**1**/poly-(*S*)-**1** (0.1 mg/mL) mixed in the presence of 0.2 mL of MeOH and 0.8mL of THF (THF/MeOH 80/20). The stereocomplexation does not take place. c) DLS traces of stereocomplex disruption by the addition of MeOH after the sterecomplexation.

[4] S. Leiras; F. Freire; J. M. Seco, E. Quiñoá and R. Riguera, *Chem. Sci.* 2013, **4**, 2735-2743.

10.3. Addition of a non-donor solvent

When $CHCl_3$ is added to a mixture of 50/50 poly-(*R*)-**1**/poly-(*S*)-**1** in THF solution, the external helices change from *P*(2/1, *c*-*t*, THF) to *P*(3/1, *c*-*c*, $CHCl_3$) — poly-(*R*)-**1**— and from *M*(2/1, *c*-*t*, THF) to *M*(3/1, *c*-*c*, $CHCl_3$) —poly-(*S*)-**1**— (Figure S33a).[4] Therefore, in this case the stereocomplex disruption (Figure S33c) is due to the transformation from a *cis-transoid* 2/1 to a *cis-cisoid* 3/1 helix.

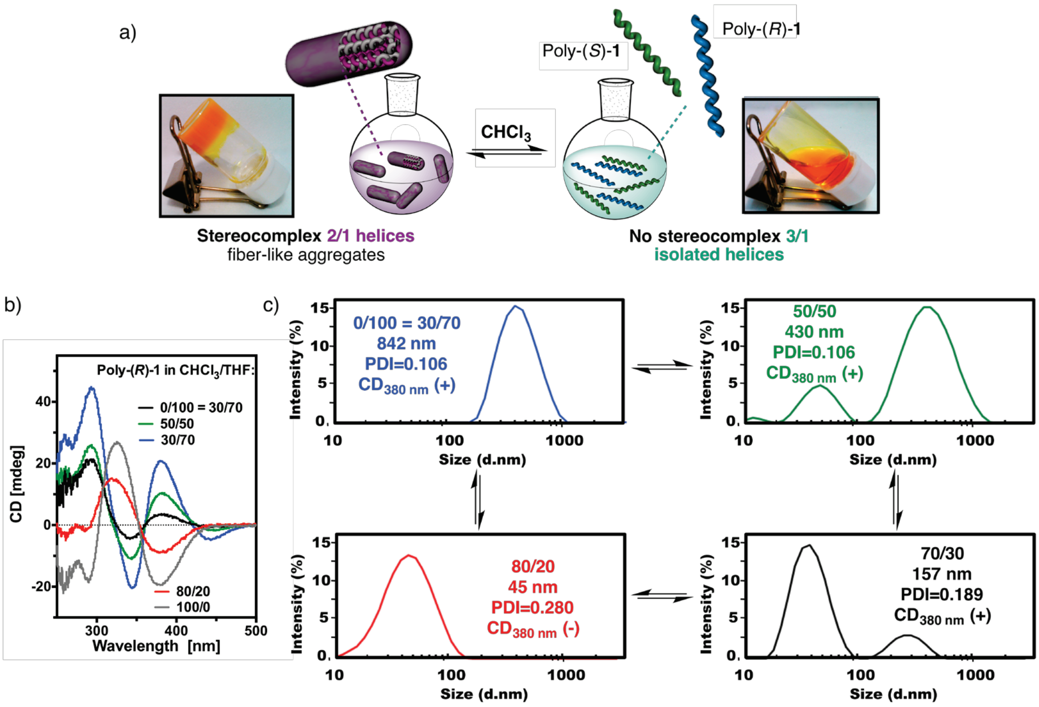

Figure S33: a) Graphical scheme showing the stereocomplex disruption through the sequential addition of $CHCl_3$ to the THF solution of the stereocomplex by tuning the *cis/trans* conformation of the amide. b) CD spectra of poly-(*R*)-**1** at different THF/$CHCl_3$ ratios showing the *c*-t (2/1, *cis*-amide) to *c*-c (3/1, *trans*-amide) tuning of the helix. c) DLS traces showing 50/50 (v/v) poly-(*R*)-**1**/poly-(*S*)-**1** at different $CHCl_3$/THF ratios. Stereocomplexation does not take place when $CHCl_3$ amount exceeds 50%.

11. Poly-(*R*)-1 + Poly-(*S*)-1 aggregation studies by Transmission Electron Microscopy (TEM) in THF

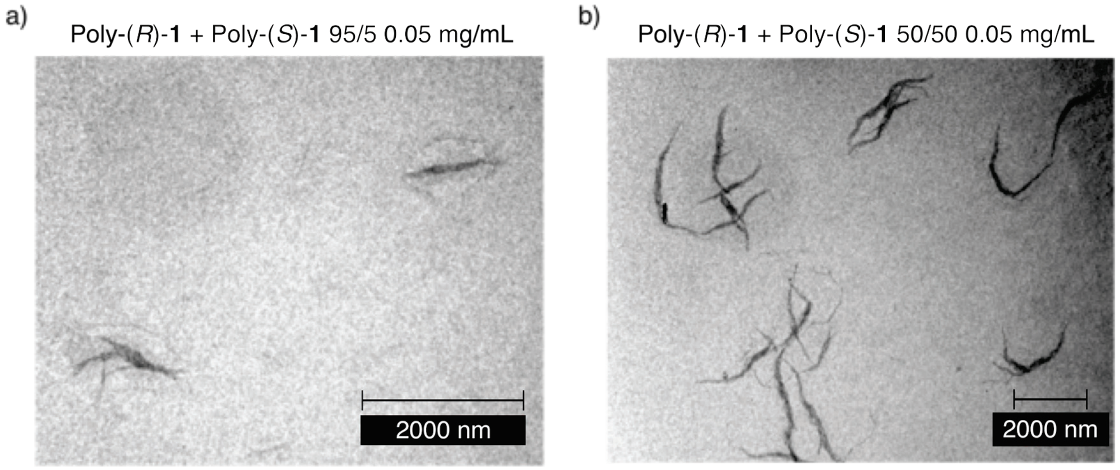

Figure S34: TEM images of stereocomplex formation at 0.05 mg/mL of different poly-(*R*)-**1** / poly-(*S*)-**1** ratios.

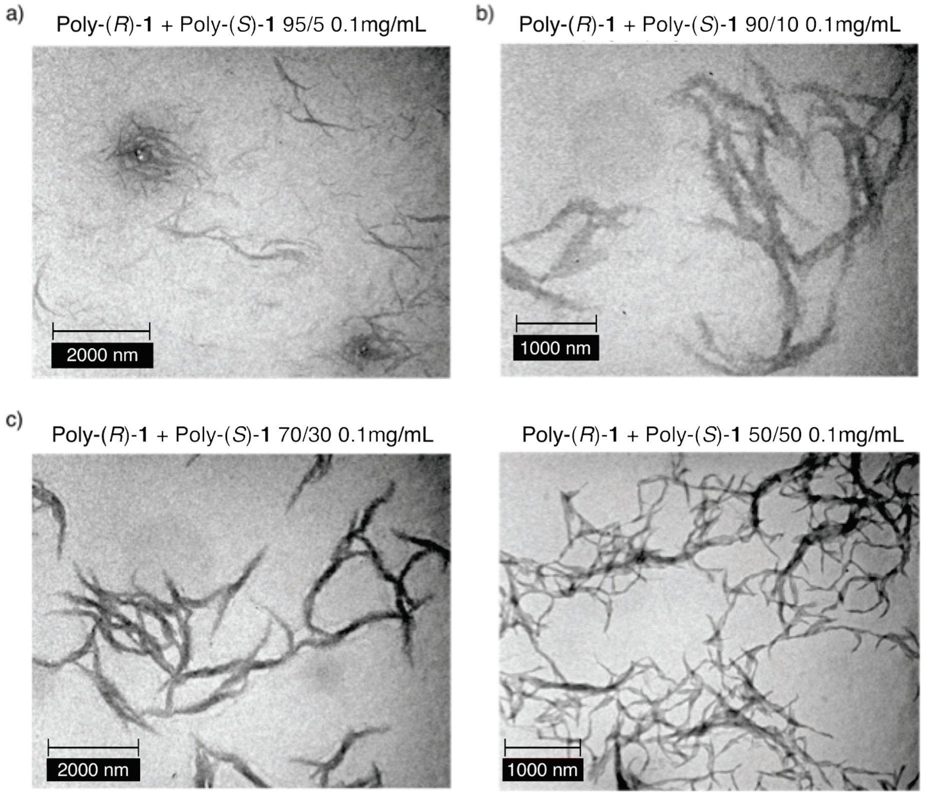

Figure S35: TEM images of stereocomplex formation at 0.1 mg/mL of different poly-(*R*)-**1** / poly-(*S*)-**1** ratios.

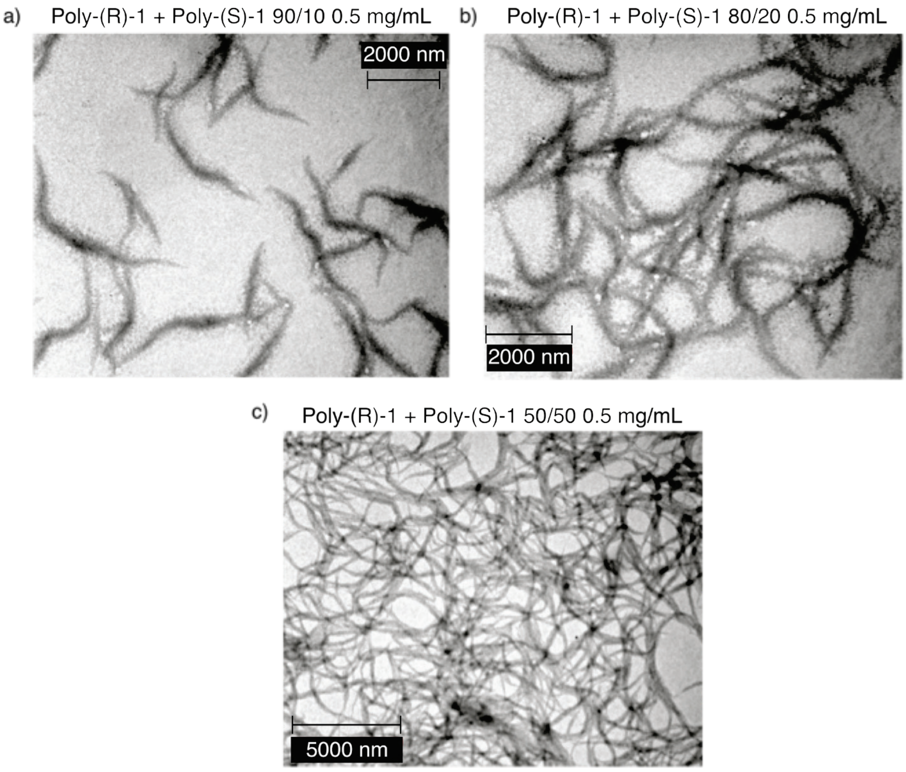

Figure S36: TEM images of stereocomplex formation at 0.5 mm/mL of different poly-(*R*)-**1** / poly-(*S*)-**1** ratios.

12. Poly-(*R*)-1 + Poly-(*S*)-1 aggregation studies by Scanning Electron Microscopy (SEM) in THF

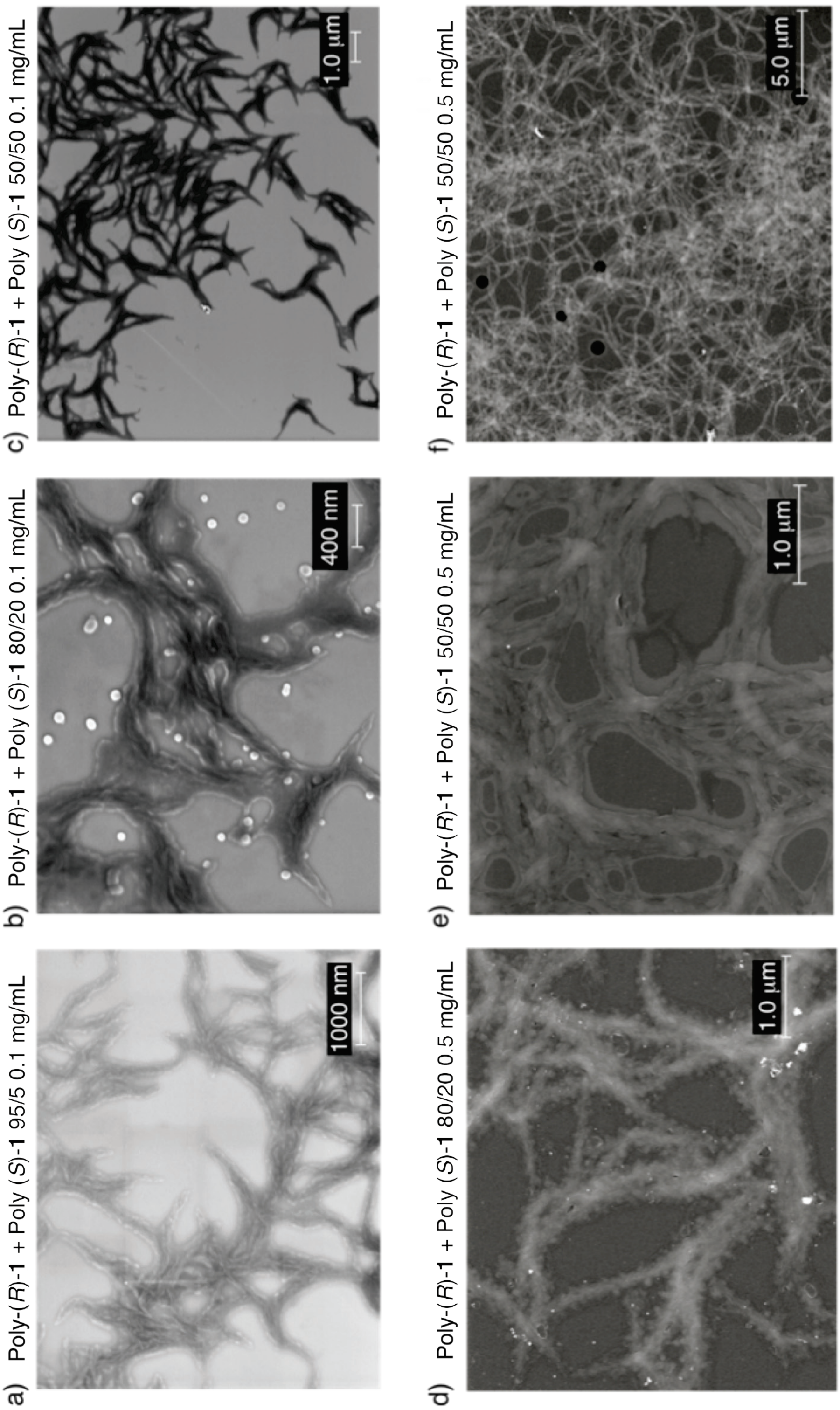

Figure S37: SEM images of stereocomplex formation at different concentrations and poly-(*R*)-**1** / poly-(*S*)-**1** ratios.

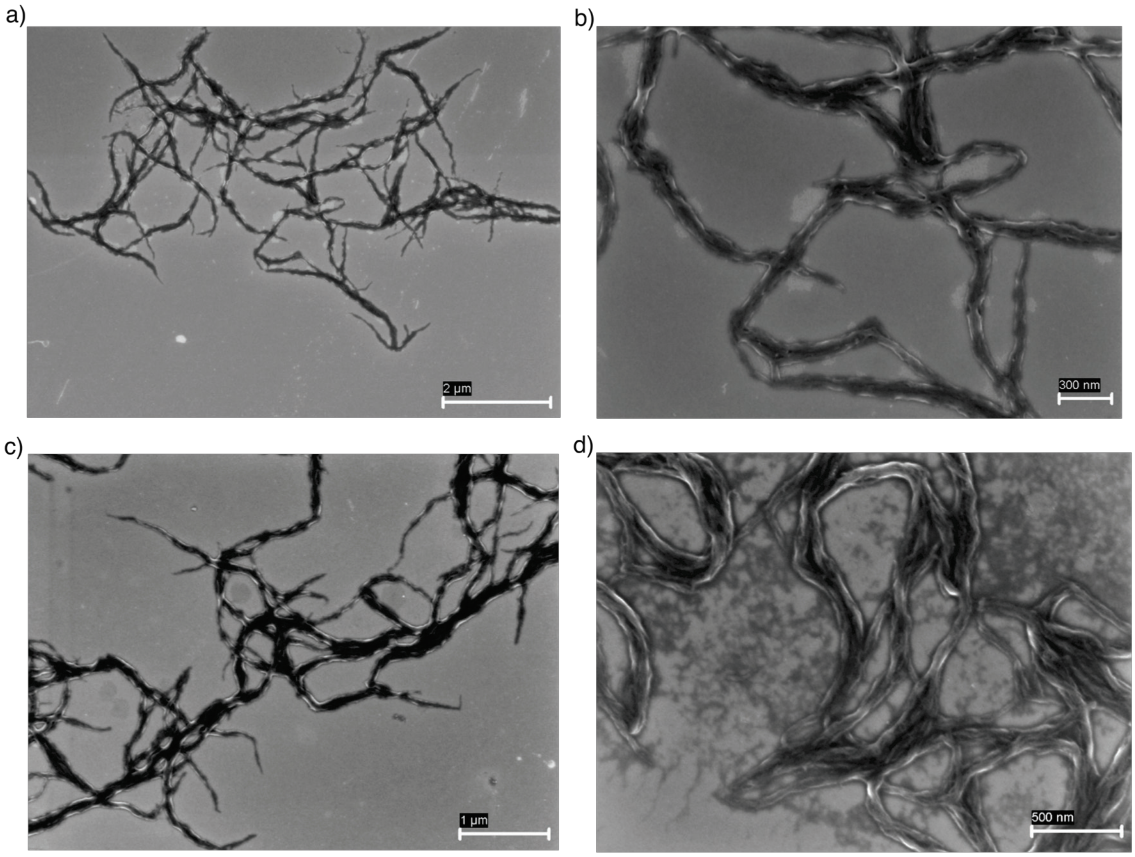

Figure S38: SEM images of stereocomplex formation at 0.01 mg/mL of poly-(*R*)-**1** / poly-(*S*)-**1** 70 /30 ratio.

13. Poly-(*R*)-1 + Poly-(*S*)-1 aggregation studies by Scanning Electron Microscopy (SEM) in $CHCl_3$ and DMF.

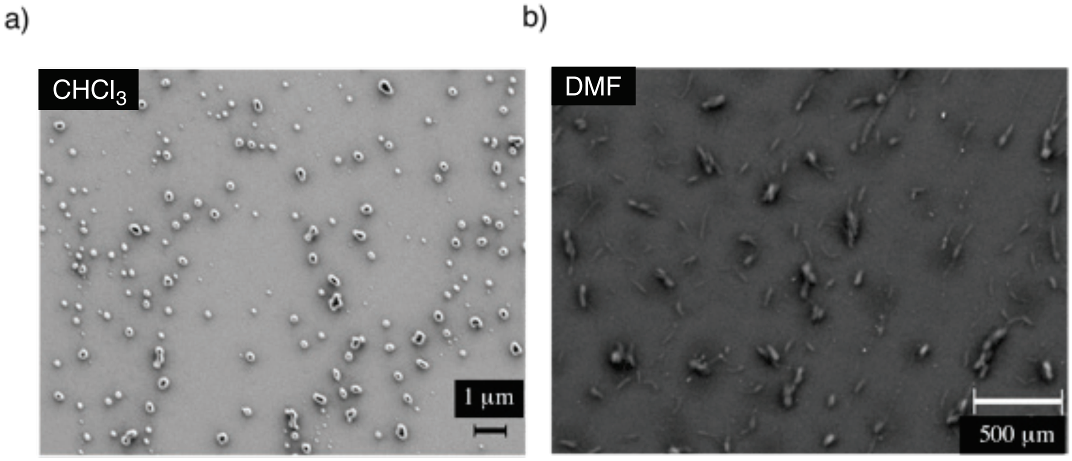

Figure S39: SEM images of poly-(*R*)-**1** / poly-(*S*)-**1** 50 /50 ratio aggregation at 0.06 mM in DMF and $CHCl_3$.

14. Poly-(*R*)-1 + Poly-(*S*)-1 aggregation studies by Atomic Force Microscopy (AFM) in THF

In order to have further information about the aggregation mechanism AFM were performed. For dis purpose, a dilute solution of [poly-(*R*)-**1**/poly-(*S*)-**1** 50/50 v/v, 0.01 mg/mL] was prepared in THF, and 10 ml were casted and spin coated onto a highly oriented polygraphite (HOPG) matrix. The results obtained revealed areas where a random mixture of the isolated right-handed or left-handed helices was found.

In addition, fibers of different size were present, corresponding to different aggregation states.

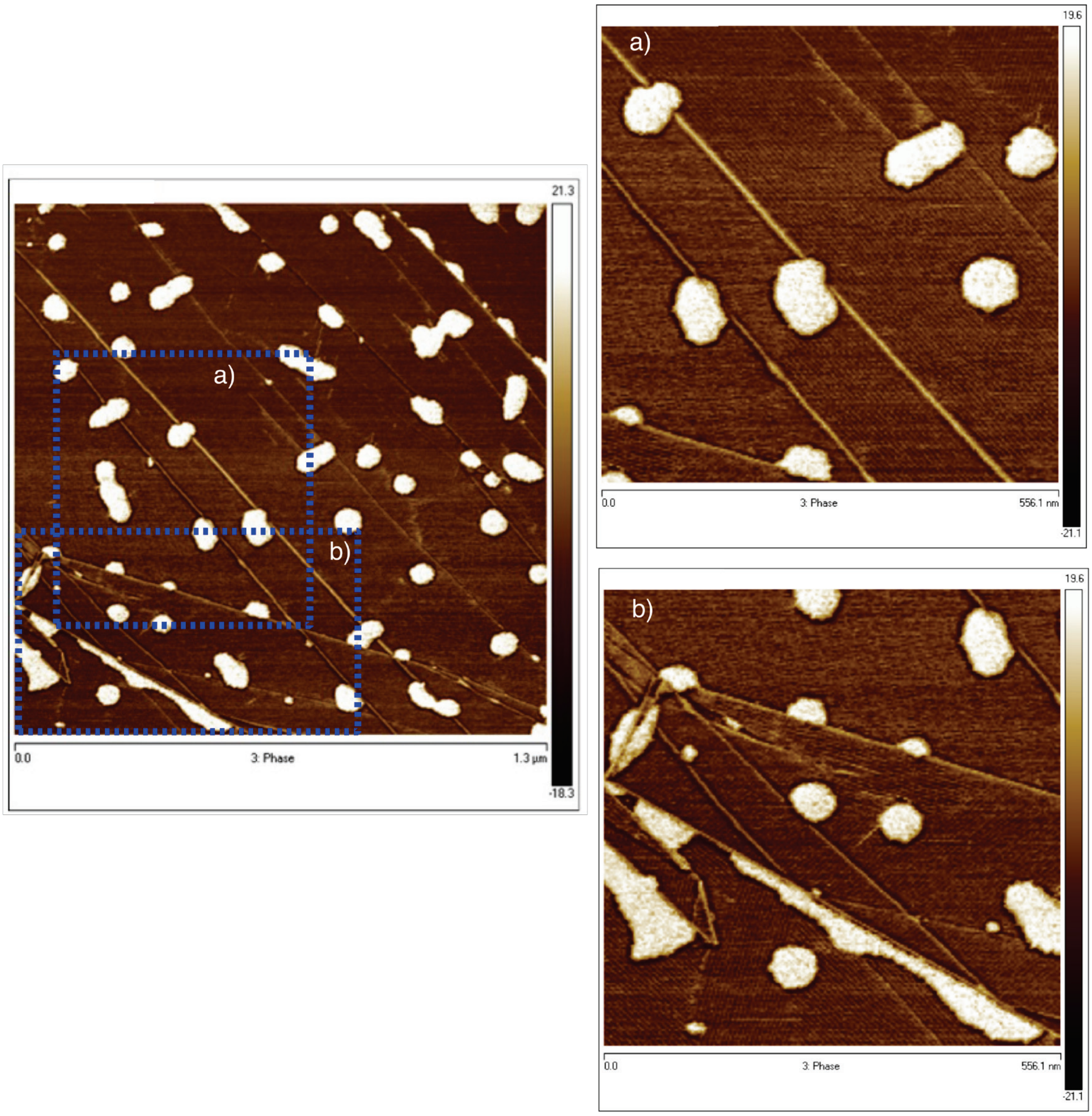

Figure S40: AFM images of 0.01 mg/mL of 50 /50 ratio poly-(*R*)-**1** / poly-(*S*)-**1** sample spin coated onto a HOPG surface, where it is possible to observe single helices areas and fibers with different sizes.

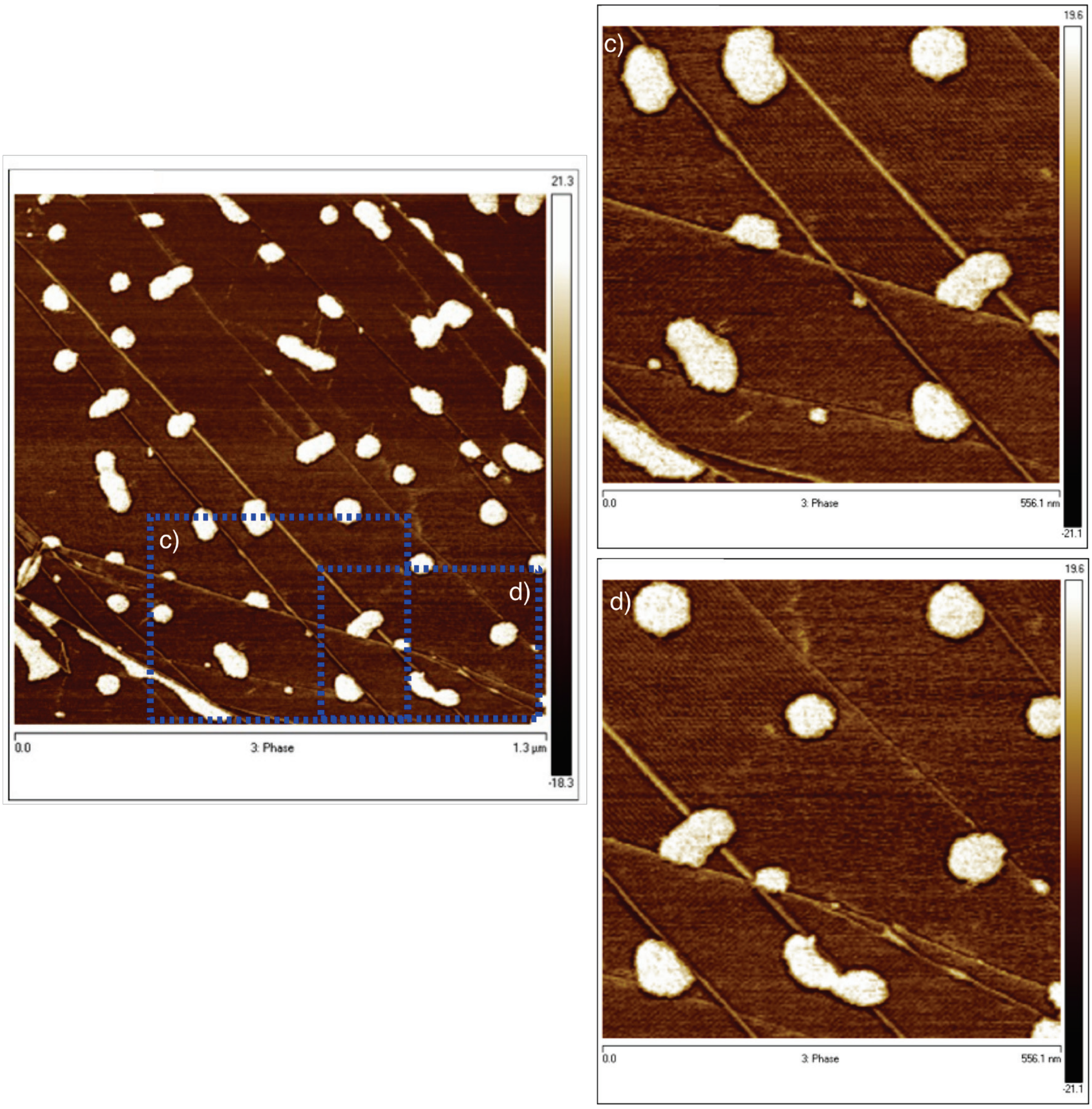

Figure S41: AFM images of 0.01 mg/mL of 50 /50 ratio poly-(*R*)-**1** / poly-(*S*)-**1** sample spin coated onto a HOPG surface, where it is possible to observe single helices areas and fibers with different sizes.

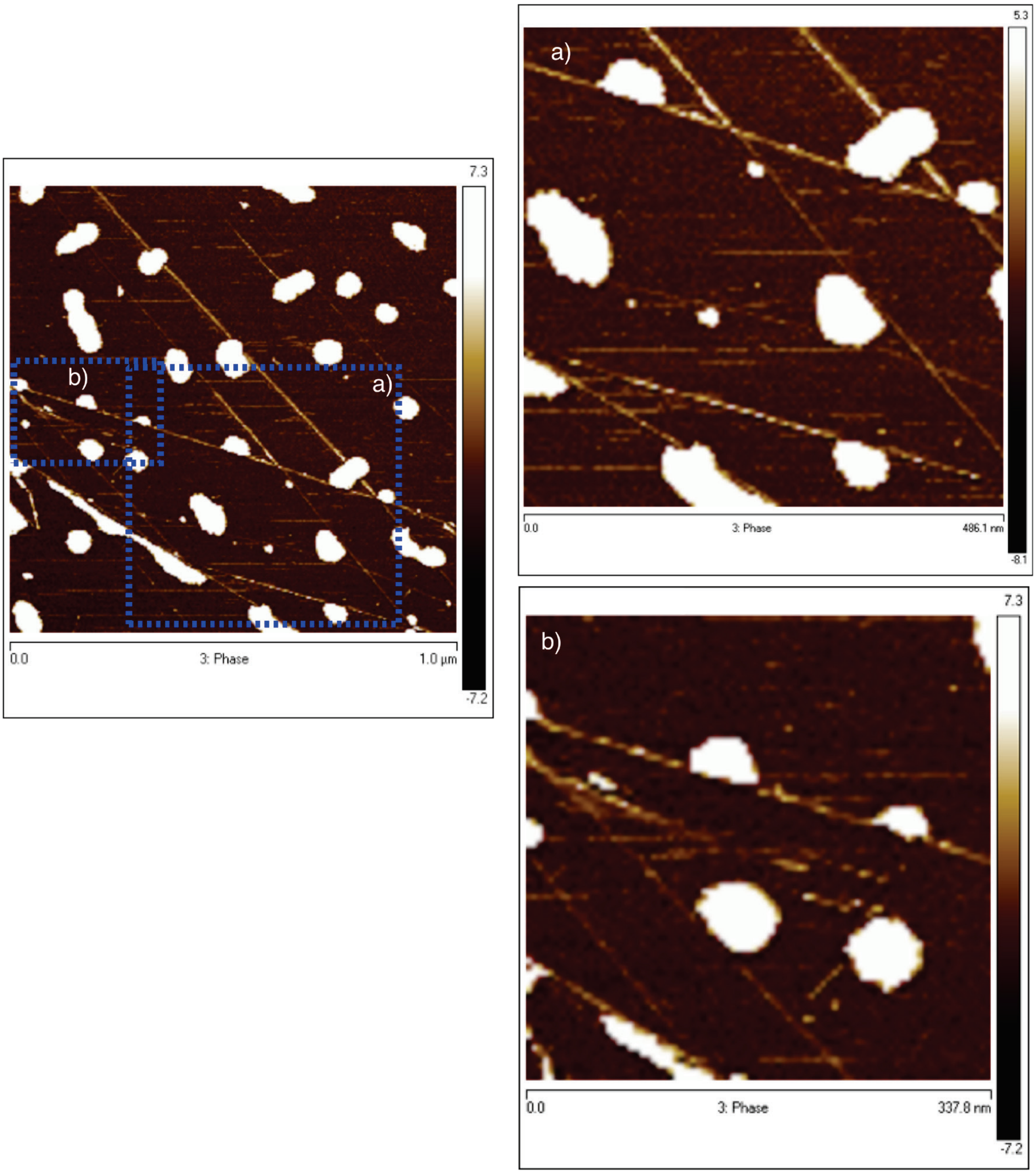

Figure S42: AFM images of 0.01 mg/mL of 50 /50 ratio poly-(*R*)-**1** / poly-(*S*)-**1** sample spin coated onto a HOPG surface, where it is possible to observe single helices areas and fibers with different sizes.

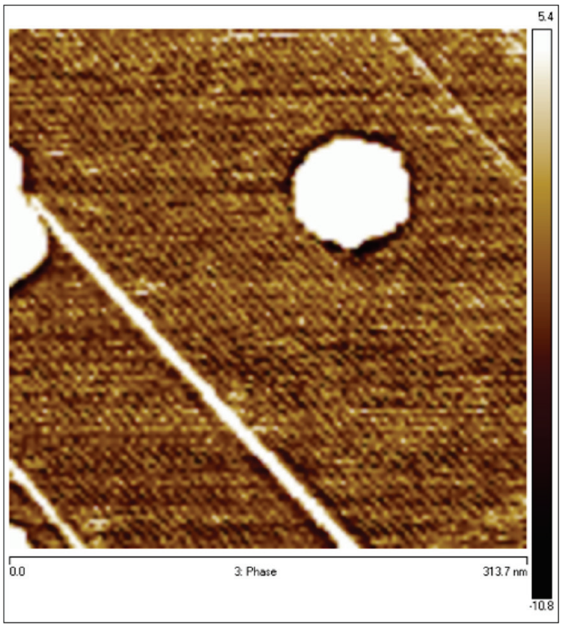

Figure S43: AFM image of 0.01 mg/mL of 50 /50 ratio poly-(*R*)-**1** / poly-(*S*)-**1** sample spin coated onto a HOPG surface, where it is possible to observe single helices areas and fibers with different sizes.

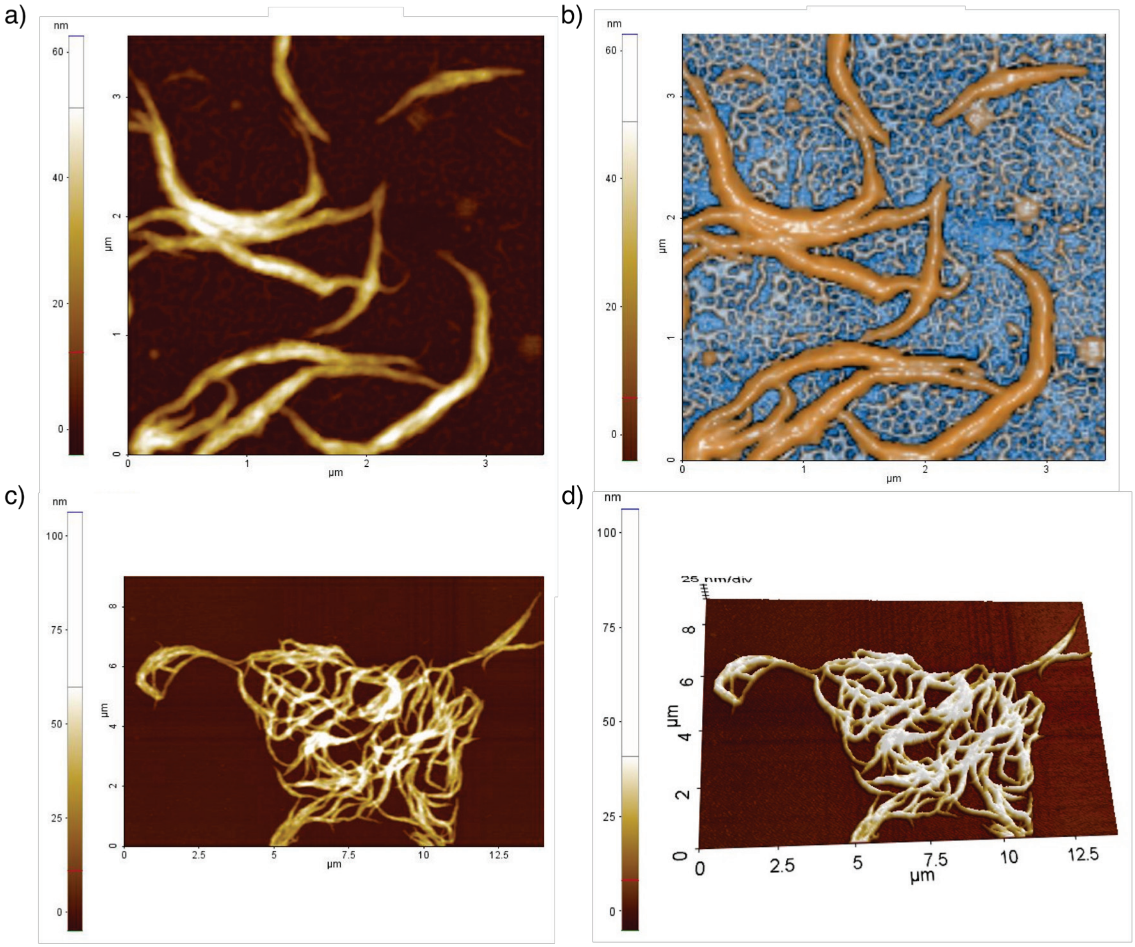

Figure S44: AFM images of 0.01 mg/mL of 50 /50 ratio poly-(*R*)-**1** / poly-(*S*)-**1** sample spin coated onto a silicon wafer surface, where it is possible to observe single helices areas and fibers with different sizes.

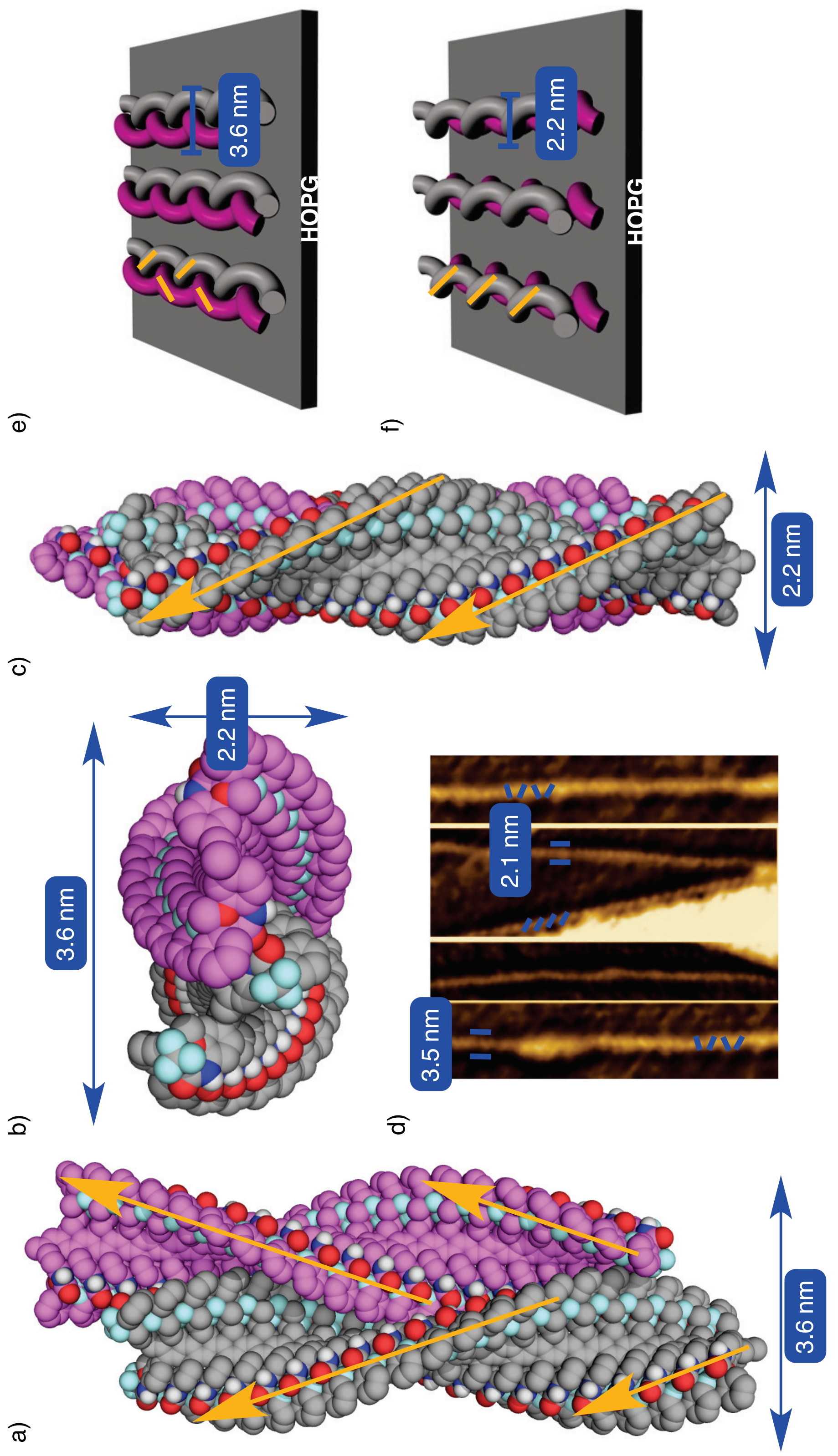

Figure S45: Geometrical matching of poly-(*R*)-**1** / poly-(*S*)-**1** duplex by computer modeling a) front view, b) top view and c) side view. d) AFM images corresponding to a poly-(*R*)-**1** / poly-(*S*)-**1** duplex aggregation state. e) and f) schematic representation of the poly-(*R*)-**1** / poly-(*S*)-**1** helices disposition on the HOPG surface.

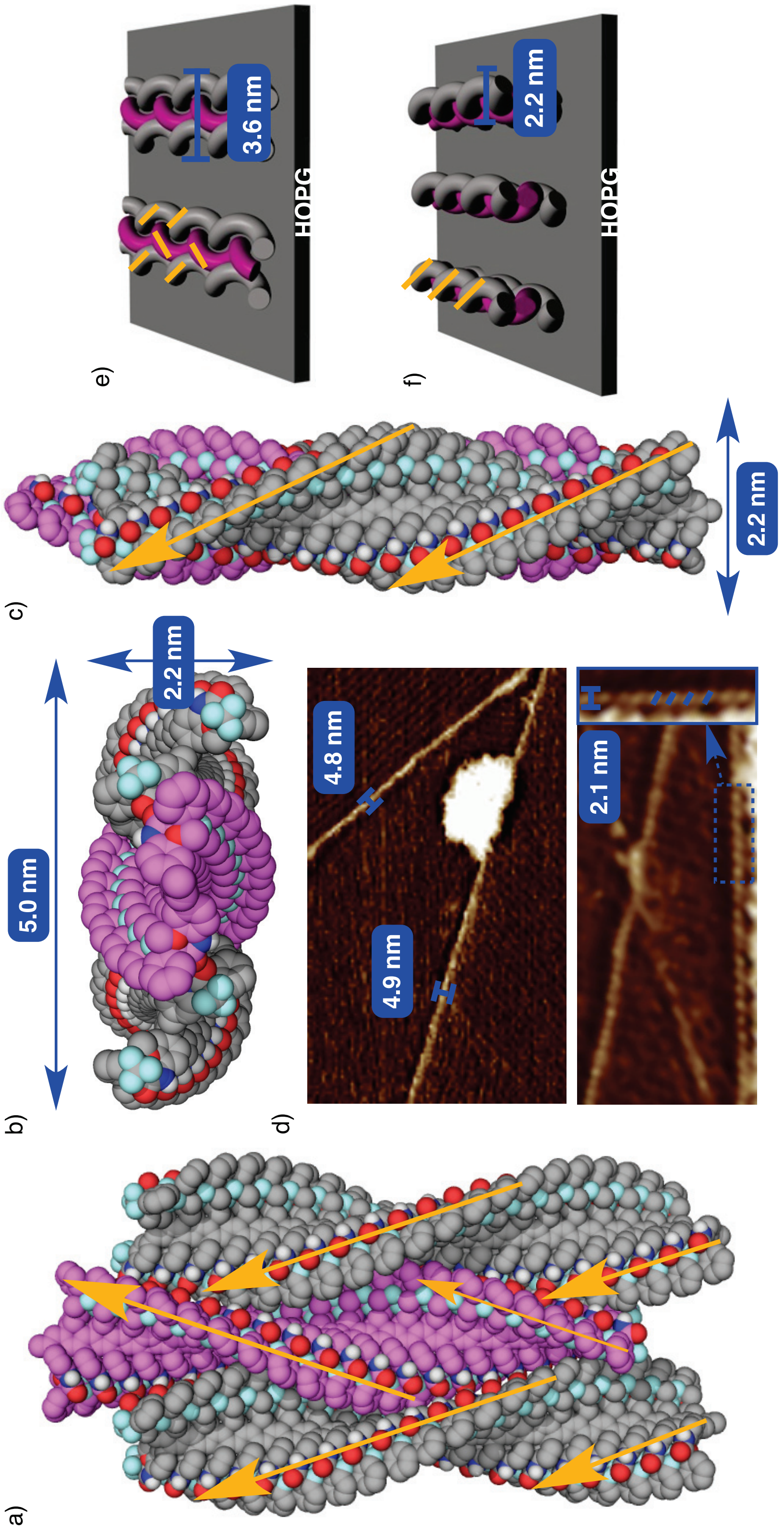

Figure S46: Geometrical matching of poly-(*R*)-**1** / poly-(*S*)-**1** triplex by computer modeling a) front view, b) top view and c) side view. d) AFM images corresponding to a poly-(*R*)-**1** / poly-(*S*)-**1** triplex aggregation state. e) and f) schematic representation of the poly-(*R*)-**1** / poly-(*S*)-**1** helices disposition on the HOPG surface.

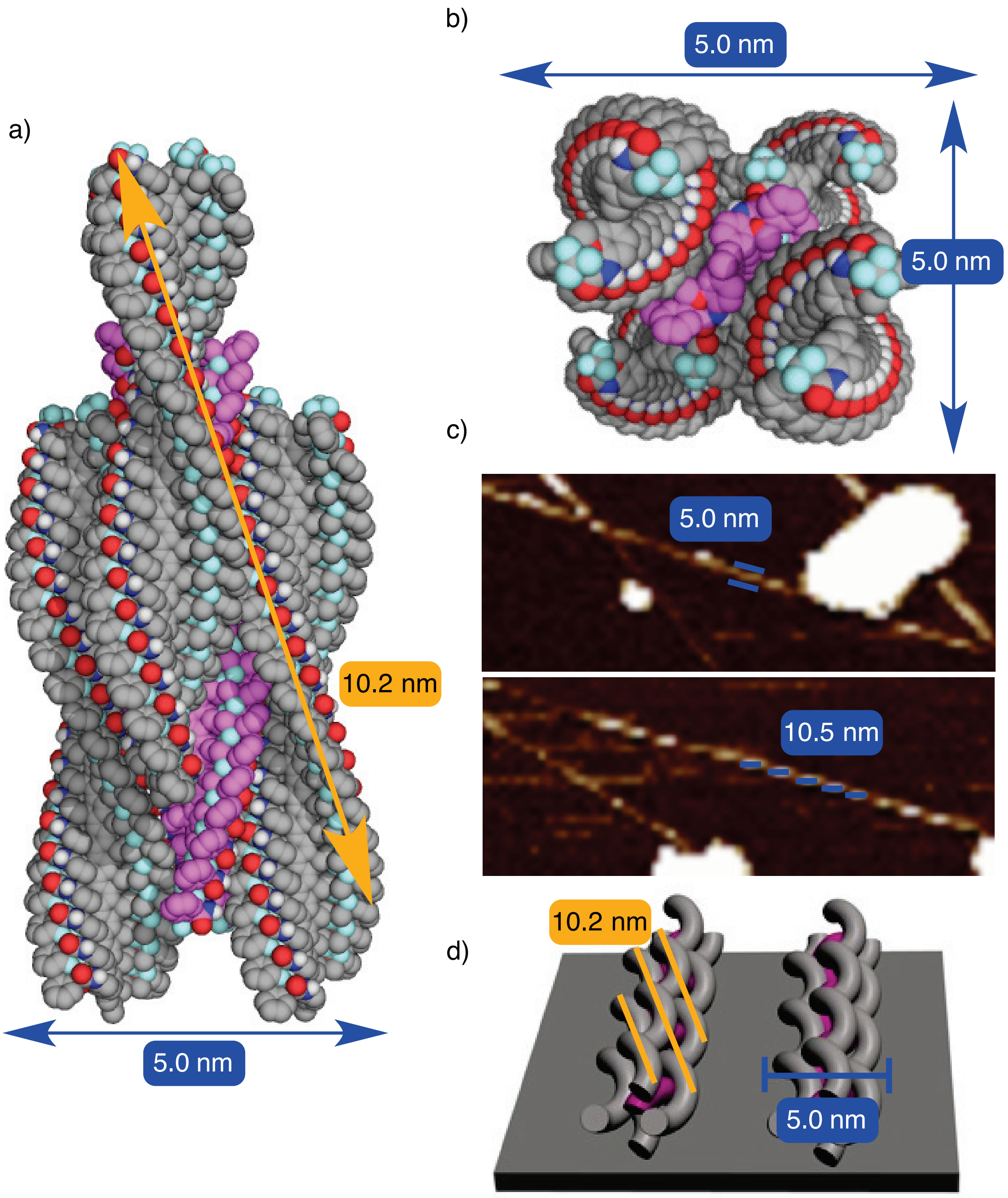

Figure S47: Geometrical matching of poly-(*R*)-**1** / poly-(*S*)-**1** quintuplet by computer modeling a) front view and b) top view. c) AFM images corresponding to a poly-(*R*)-**1** / poly-(*S*)-**1** quintuplet aggregation state. d) schematic representation of the poly-(*R*)-**1** / poly-(*S*)-**1** helices disposition on the HOPG surface.

15. Rheology studies

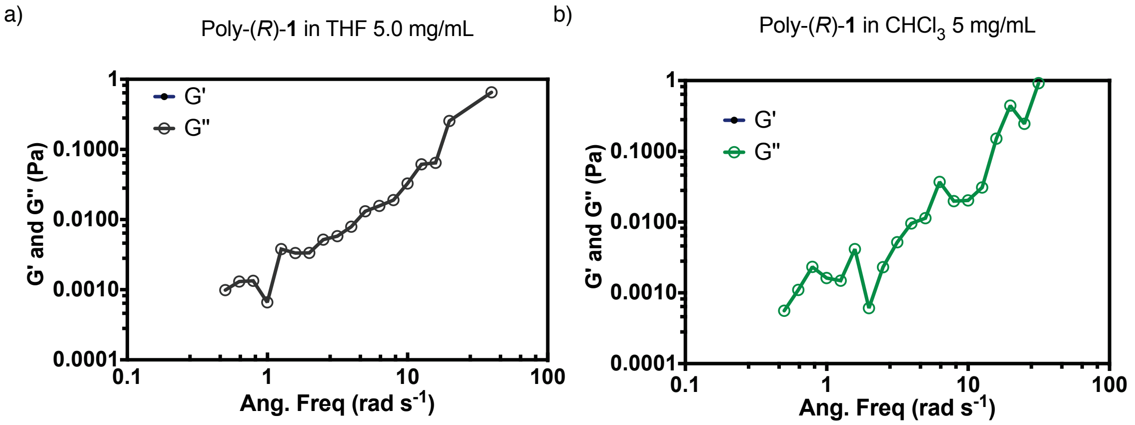

Figure S48: Viscoelastic measurements of poly-(*R*)-**1** solutions in a) THF and b) $CHCl_3$.

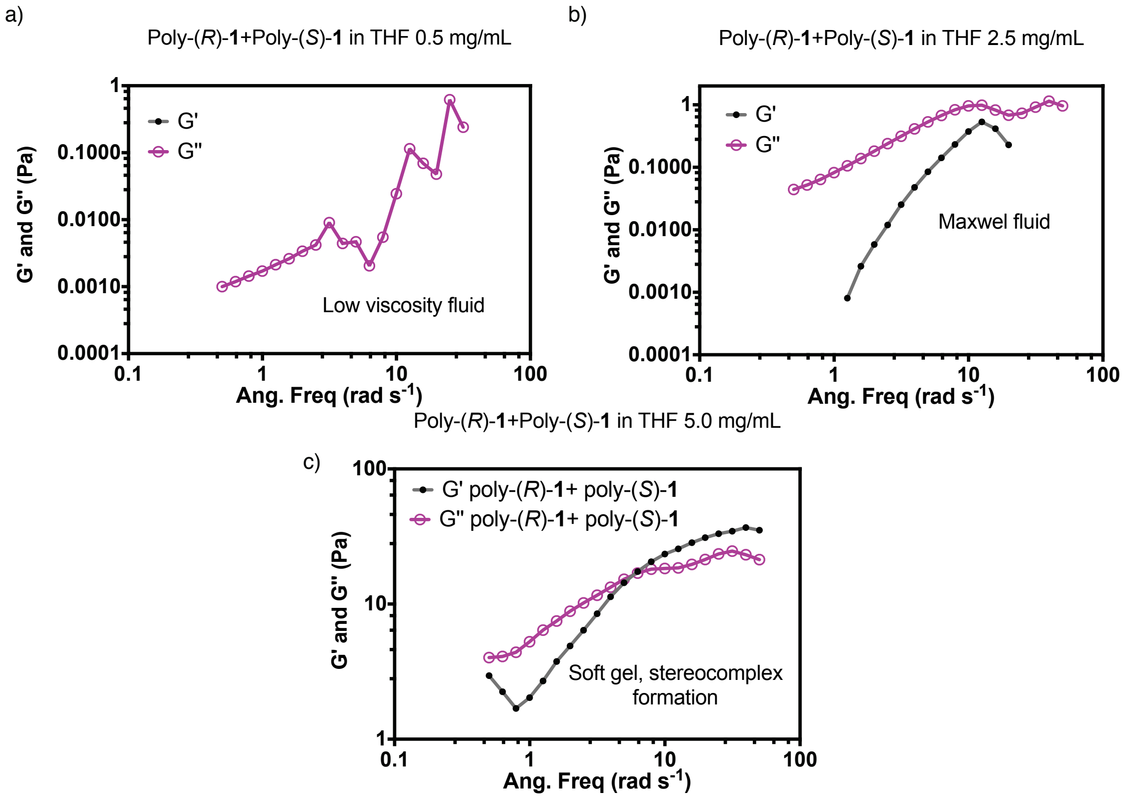

Figure S49: Viscoelastic measurements of poly-(*R*)-**1** / poly-(*S*)-**1** 50 /50 ratio solutions in THF at different concentrations.

16. Poly-[(*R*)-1-co-2] and Poly-[(*S*)-1-co-2] aggregation studies by Dynamic Light Scattering (DLS) in THF

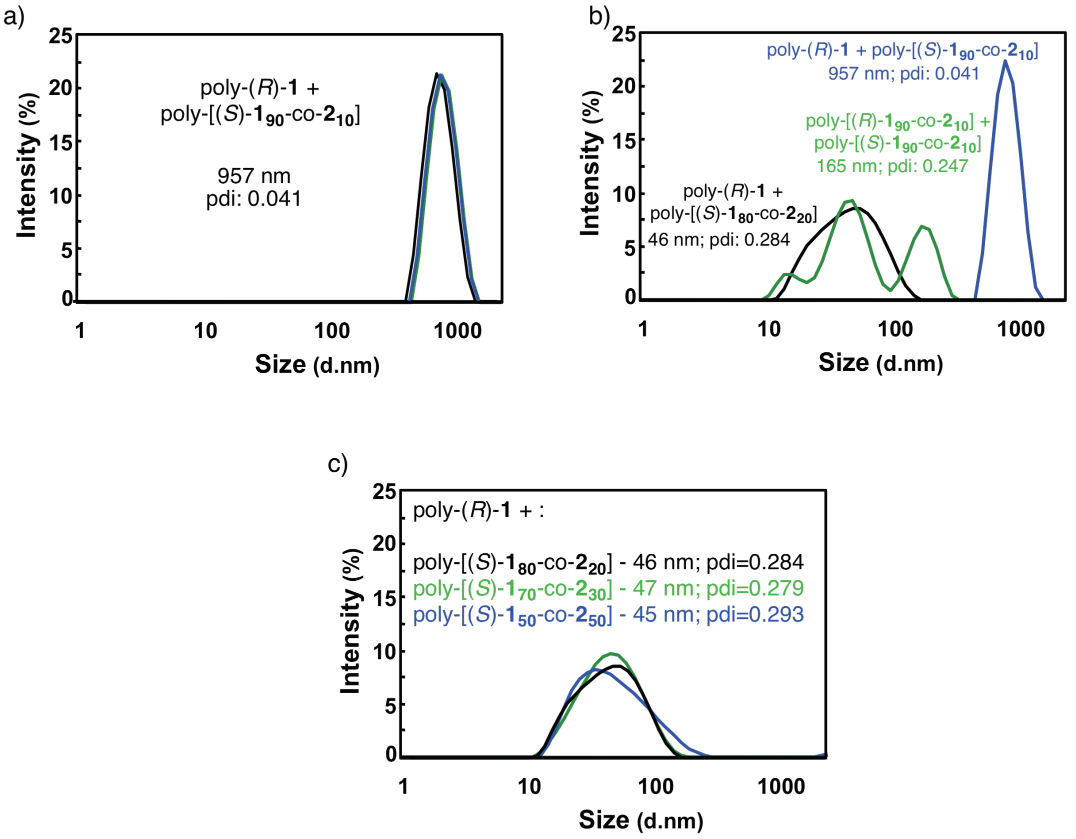

Figure S50: DLS studies of poly-[(*R*)-**1**$_x$-co-**2**$_{1-x}$] and poly-[(*S*)-**1**$_x$-co-**2**$_{1-x}$] aggregation, 0.1 mg/mL in THF, 50 / 50 ratio.

17. Poly-[(*R*)-1-co-2] and Poly-[(*S*)-1-co-2] aggregation studies by Scanning Electron Microscopy (SEM) in THF

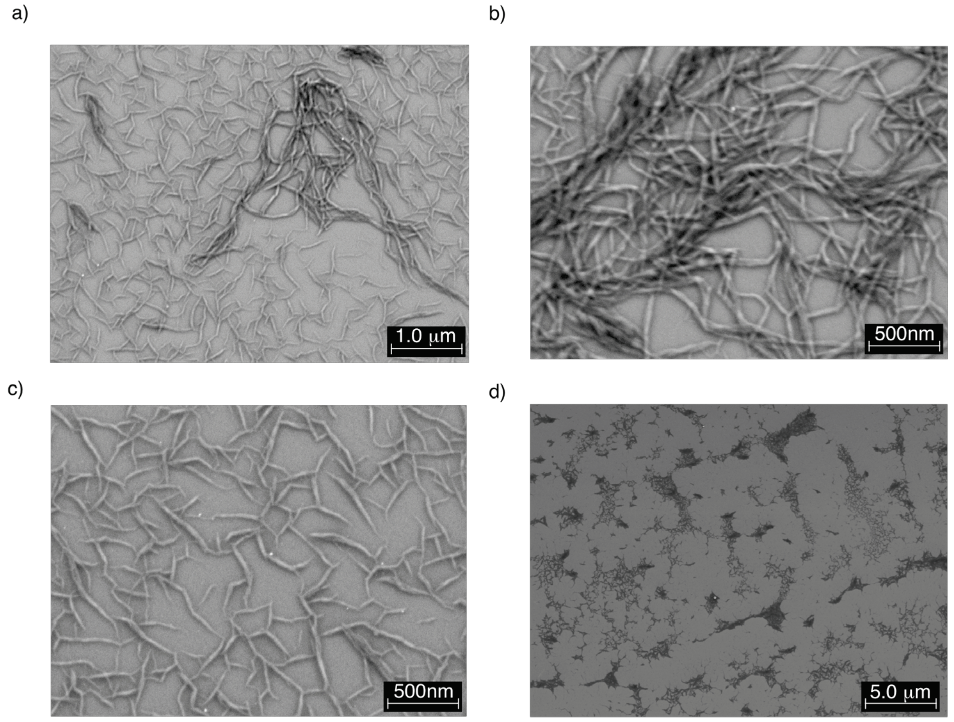

Figure S51: SEM images of poly-[(*R*)-**1**$_{90}$-co-**2**$_{10}$] and poly-(*S*)-**1** aggregation in THF at 0.1 mg/mL.

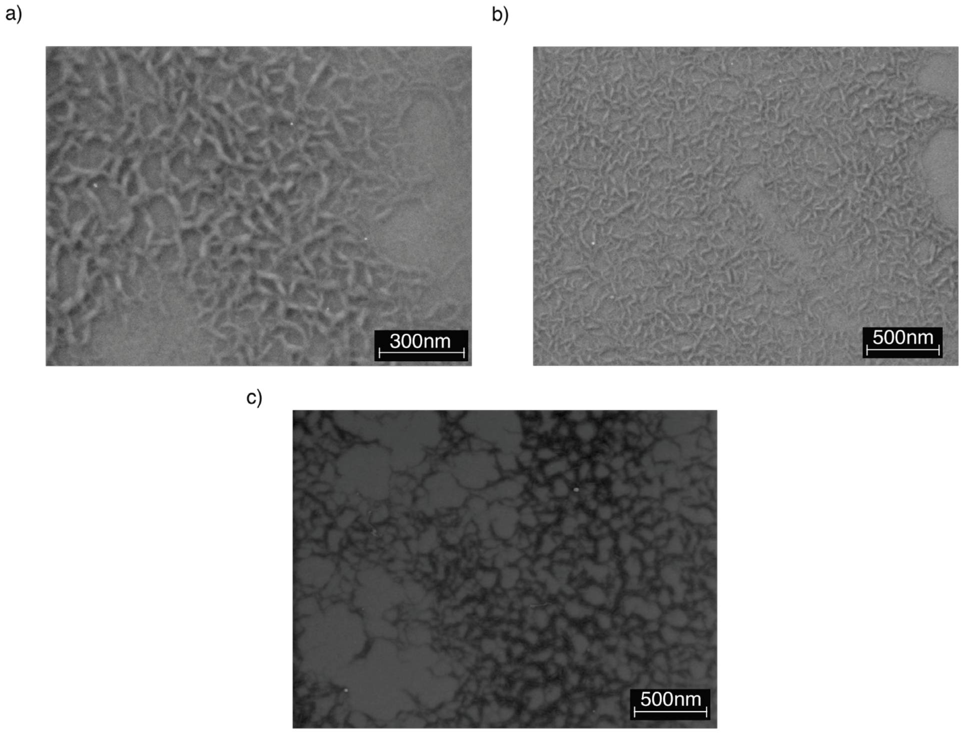

Figure S52: SEM images of poly-[(*R*)-**1**$_{90}$-co-**2**$_{10}$] and poly-[(*S*)-**1**$_{90}$-co-**2**$_{10}$] aggregation in THF at 0.1 mg/mL concentration.

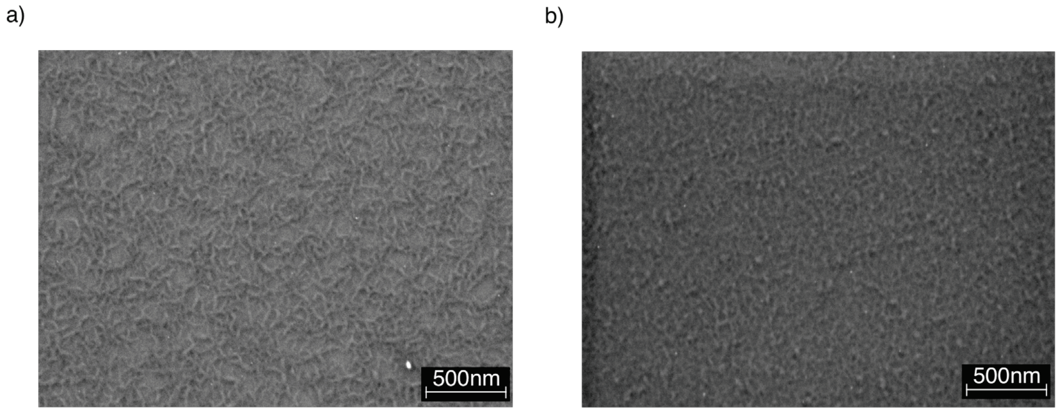

Figure S53: SEM images of poly-[(*R*)-$\mathbf{1}_{80}$-co-$\mathbf{2}_{20}$] and poly-(*S*)-**1** aggregation in THF at 0.1 mg/mL concentration.

18. Differencial Scaning Calorimetry (DSC) studies of Poly-(*R*)-1 and [Poly-(*R*)-1 + Poly-(*S*)-1] films

18.1. Poly-(*R*)-1 film prepared in $CHCl_3$ and THF:

DSC experiments were performed for poly-(*R*)-**1** films prepared in THF and $CHCl_3$. The DSC heating traces (300ºC, 10ºC/min) obtained for both films reveal different conformations: *c-t* for THF and *c-c* for $CHCl_3$ (See Figure S54).

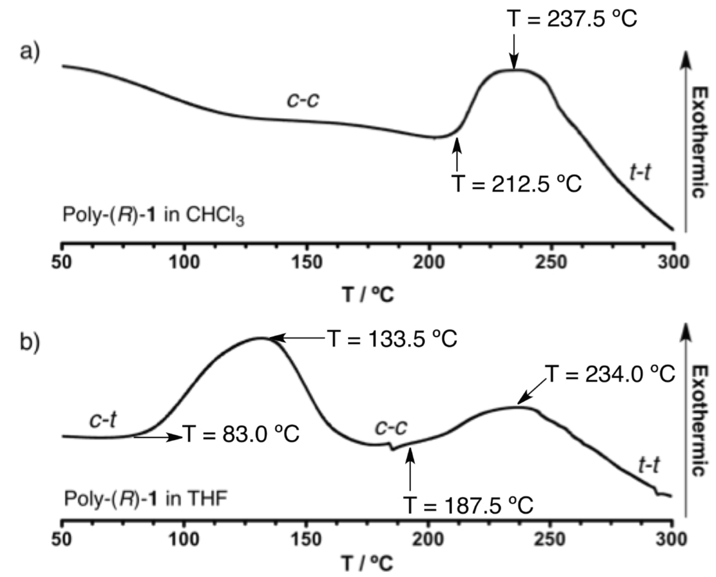

Figure S54: DSC traces of poly-(*R*)-**1** film prepared in $CHCl_3$ and THF

Both samples were allowed to cool down to 20 ºC at 10ºC/min where no transition peaks were observed (Figure S55).

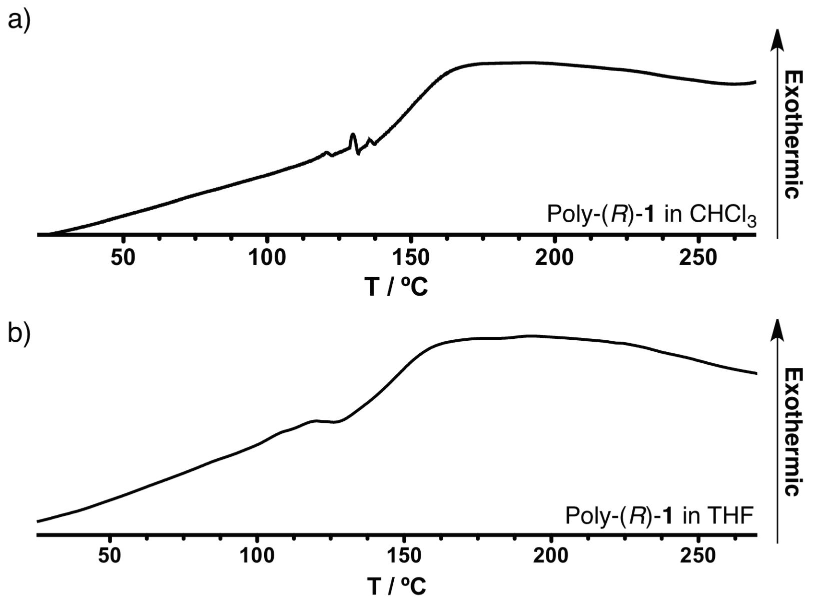

Figure S55: DSC cooling traces of poly-(*R*)-**1** films prepared in a) $CHCl_3$ and b) THF.

Finally a second heating was done in order to check the reversibility of the process (300ºC, 10ºC/min). Identical DSC traces were obtained for both films, showing the DSC pattern of a *t-t* isomer corresponding to the last transition observed during the first heating in both cases (Figure S56). This fact indicates that the process is not reversible.

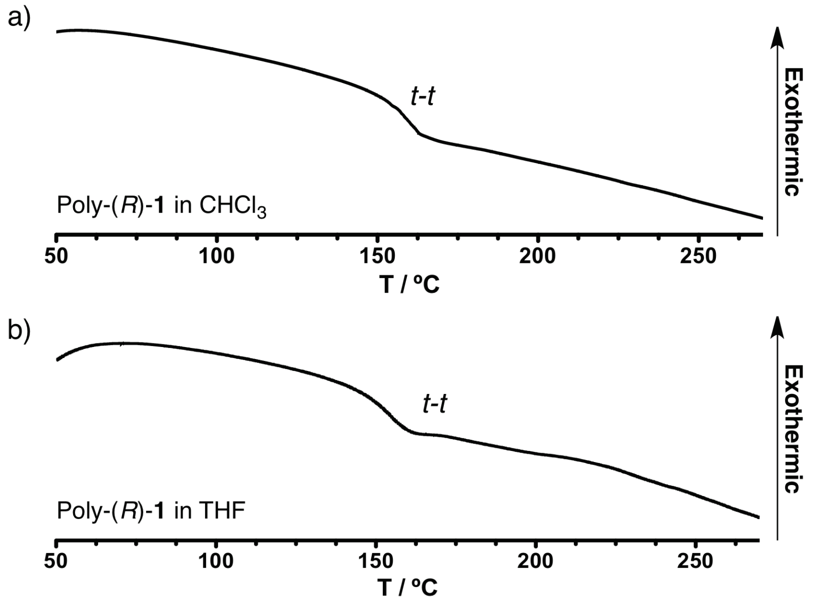

Figure S56: DSC second heating traces of poly-(*R*)-**1** films prepared in a) $CHCl_3$ and b) THF.

18.2. [Poly-(*R*)-1 + Poly-(*S*)-1] film prepared in $CHCl_3$ and THF

DSC experiments were performed for [poly-(*R*)-**1** + poly-(*S*)-**1**] films prepared in THF and $CHCl_3$. In order to favour a good annealing for the polymers, poly-(*R*)-**1** + poly-(*S*)-**1** 50/50 films were prepared at 40 ºC from the corresponding starting solutions of 30 mg/mL. The polymer mix was kept at 50ºC for 15 min. and then it was allowed to cool down at 5ºC/min to room temperature. Finally, 100 μL of the final solution were slowly evaporated in an aluminum pan. The DSC heating traces were recorded at 10 ºC/min from 0 ºC to 300 ºC. The results show identical signature for poly-(*R*)-**1** and [poly-(*R*)-**1** + poly-(*S*)-**1**] films prepared in $CHCl_3$, this fact confirms the non-sterecomplexation process (see Figure S57). However, [poly-(*R*)-**1** + poly-(*S*)-**1**] film prepared in THF present a different DSC thermogram from its parent polymer: the beginning of the *c-t* to *c-c* isomerization transition suffers a delay of 30 ºC approximately respect the poly-(*R*)-**1** film, indicating a higher stability for the new material (see Figure S58).

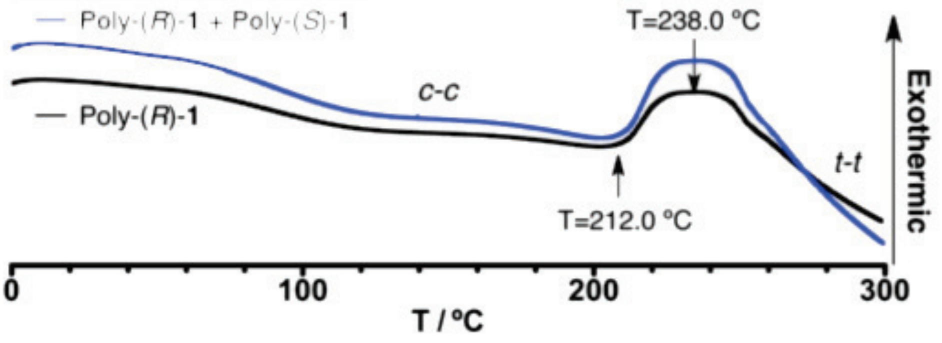

Figure S57:DSC traces of poly-(*R*)-**1** and [poly-(*R*)-**1** + poly-(*S*)-**1**] film prepared in $CHCl_3$.

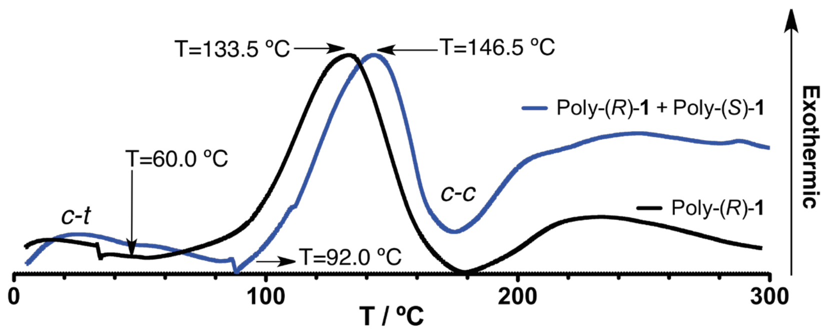

Figure S58: DSC traces of poly-(*R*)-**1** and poly-(*R*)-**1** + poly-(*S*)-**1** film prepared in THF.

In order to check the reversibility of the process, the sterecomplex sample was allowed to cool down to 20 ºC at 10 ºC/min and a second heating was done at 10ºC/min to 300ºC (see Figure S59). The cooling process does not reveal any transition peak and furthermore the second heating process shows the classical patter for a t-t isomer, indicating that the transitions observed during the first heating process are not reversible.

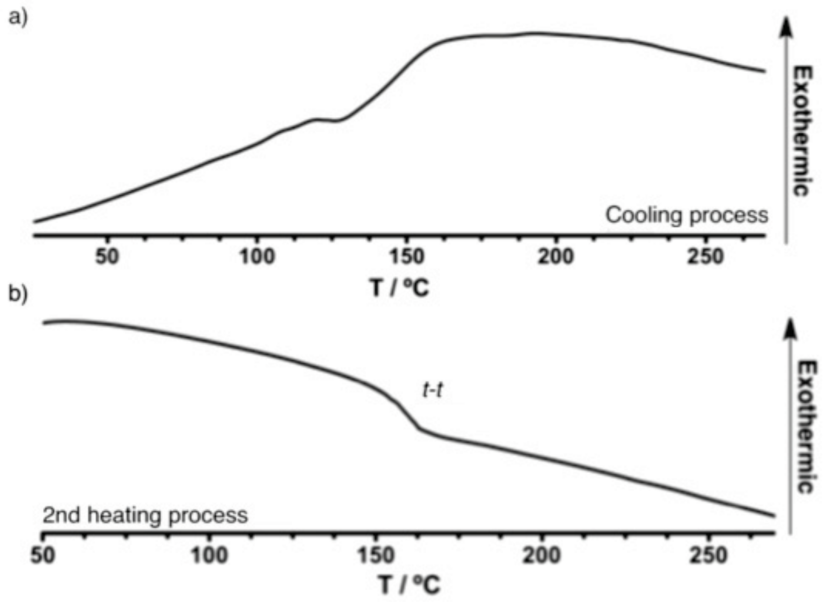

Figure S59: DSC cooling a) and second heating b) traces of stereocomplex films prepared in THF.

19.Gel-state on/off switching

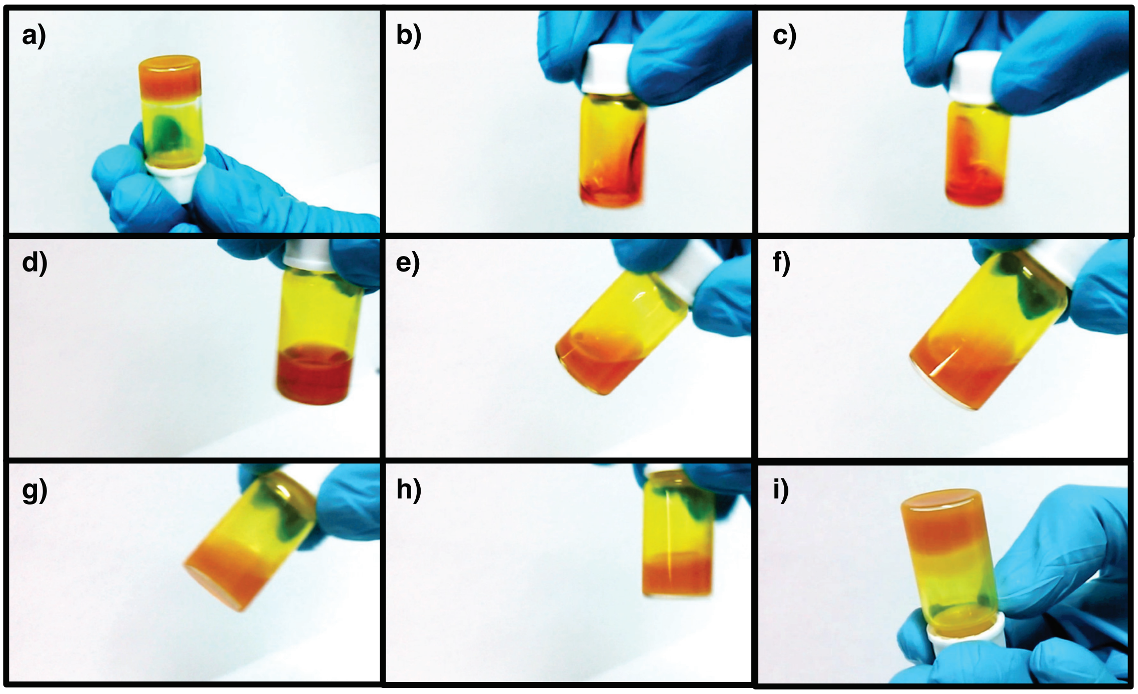

Figure S60: ON/OFF switching of the stereocomplex gel-state through temperature control: a) Gel-state stereocomplex at rt (concn=60 mg/mL THF). b) and c) Stereocomplex at gel-state disrupted after heating up to 60 ºC. d) to i) Stereocomplexation and gel-state recovery after cooling down from 60 ºC to rt.

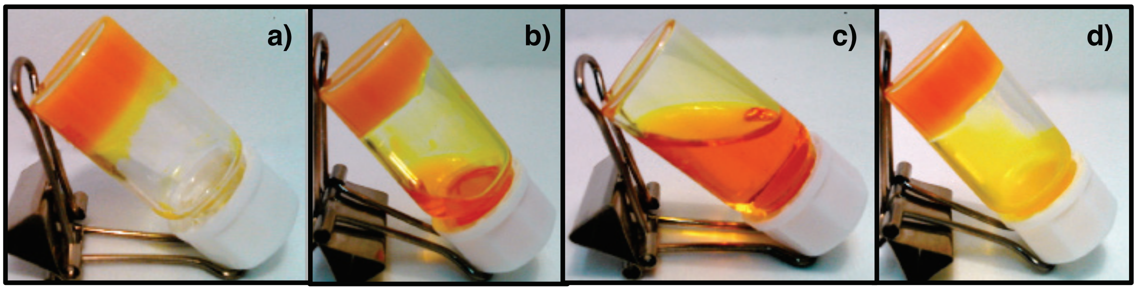

Figure S61: ON/OFF switching of the stereocomplex gel-state by addition of a non-donor solvent (CH_2Cl_2): a) Gel-state stereocomplex (concn=60 mg/mL THF). b) Gel-state stereocomplex and disrupted stereocomplex after the addition of 0.3 mL of CH_2Cl_2. c) Disrupted stereocomplex after the addition of 0.8 mL of CH_2Cl_2. d)Stereocomplexation and gel-state recovery after CH_2Cl_2 slow evaporation.

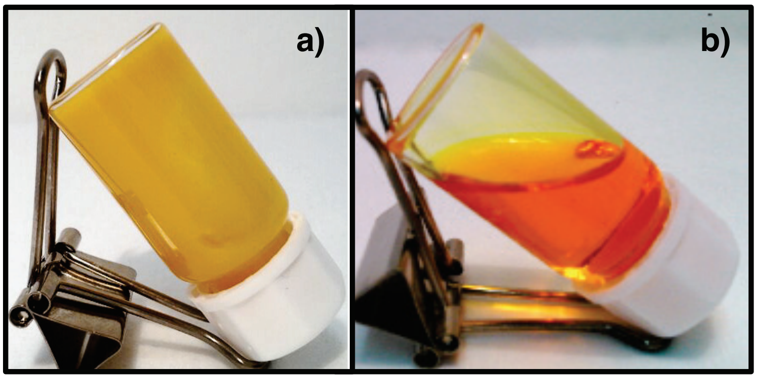

Figure S62: a) Stereocomplex gel-state (concn=30 mg/mL THF). b) Disrupted stereocomplex (concn=30 mg/mL THF/CH_2Cl_2 50/50). These pictures demonstrate how the addition of THF (1 mL) to the stereocomplex gel-state (concn=60 mg/mL THF) does not disrupt the aggregation, while the addition of the same amount of CH_2Cl_2 does.
